# Binding affinities for histamine receptors 1 to 4: Systematic comparison of ligands from the Psychoactive Drug Screening Program K_i_ database and International Union of Basic and Clinical Pharmacology/British Pharmacological Society Guide to Pharmacology

**DOI:** 10.1016/j.molpha.2026.100139

**Published:** 2026-06-12

**Authors:** Lilly Josephine Bindel, Roland Seifert

**Affiliations:** Institute of Pharmacology, Hannover Medical School, Hannover, Germany

**Keywords:** Histamine, G protein coupled receptors, Ligand, Binding affinity, Receptor, IUPHAR

## Abstract

Histamine receptors (H_1_R–H_4_R) are G protein-coupled receptors with large physiological and therapeutic relevance. Despite extensive research, a systematic and cross-database overview of ligands is missing. This review integrates the Psychoactive Drug Screening Program (PDSP) K_i_ database and the International Union of Basic and Clinical Pharmacology/British Pharmacological Society (IUPHAR/BPS) Guide to Pharmacology, both having different scopes and curation principles, and evaluates concordance of H_x_R ligands across databases and experimental systems. Binding affinities (K_i_) for 302 ligands targeting recombinant human H_x_R were extracted from the PDSP K_i_ database (accessed January 2026) and converted to pK_i_. A total of 302 ligands with relevant binding affinities were identified, including approved drugs and experimental compounds. Functional annotations and further binding affinities were retrieved from the IUPHAR/BPS Guide to Pharmacology and complementary literature, revealing missing or incomplete functional characterization for 69 ligands. Although most ligands were selective for a single H_x_R, others displayed multireceptor affinity. Cross-database comparisons found limited overlap in ligands, differences in primary literature, and pK_i_ deviations. Comparisons with native human tissues and nonhuman species demonstrated substantial variability, with deviations of up to pK_i_ 4.2 in human brain tissues and up to pK_i_ 3.2 across species. This database-driven analysis highlights strengths and limitations of the PDSP K_i_ database (broad data aggregation) and the IUPHAR/BPS Guide to Pharmacology (curated representative selection). Although together they provide extensive coverage of H_x_R ligands, incomplete overlap and selective data inclusion limit direct comparability and may give the impression of missing data despite substantial experimental data. Systematic integration is essential to improve data reliability, summarize knowledge, and support future drug development.

**Significance Statement:**

This review provides a systematic overview of ligand binding affinities and functional profiles across all 4 H_x_R. It integrates data from the PDSP K_i_ database and the IUPHAR/ BPS Guide to Pharmacology and compares recombinant human H_x_R with native tissues and several species. Substantial data gaps, inconsistencies, and context-dependent variability are revealed. The PDSP K_i_ database has large potential for use in scientific research and in the support of drug development.

## 1 Introduction

Histamine receptors belong to the G protein-coupled receptor (GPCR) family and comprise 4 subtypes (H_1_R–H_4_R).^1^^,^^2^ H_x_R are expressed across many species and in a wide range of human organs^3^^,^^4^ Individual receptor subtypes mediate distinct physiological and pathological effects, including allergic and inflammatory reactions (hH_1_R and hH_4_R), gastrointestinal acid secretion (hH_2_R), and functions within the central nervous system (CNS; hH_3_R).^1^^,^^5^ Although histamine (**182**) is the endogenous ligand capable of activating all four human H_x_R (hH_x_R), it does not interact equally with all receptor subtypes, and differences in binding affinity have been reported across hH_1_R–hH_4_R,^1^^,^^6^^,^^7^ which may reflect structural variations in the ligand binding pockets of the receptor subtypes.^8^ Furthermore, numerous other ligands have been identified that act as antagonists, agonists, or inverse agonists.^2^^,^^9^^,^^10^

Despite the extensive research on histamine receptors and their therapeutic relevance, a systematic and cross-database overview of ligand binding affinities and functional profiles across all four hH_x_R is still lacking. This minireview provides a selection and comparison of binding affinity data and functional annotations for hH_x_R, based on ligands reported in the Psychoactive Drug Screening Program (PDSP) K_i_ database and the International Union of Basic and Clinical Pharmacology/British Pharmacological Society (IUPHAR/BPS) Guide to Pharmacology. Both resources differ fundamentally in their scope, curation strategy, and intended use. The IUPHAR/BPS Guide to Pharmacology provides expert-curated, representative ligand information, whereas the PDSP K_i_ database aims to capture a broad collection of binding affinity data, including large numbers of compounds with variable levels of experimental validation.

**Fig. 1:**
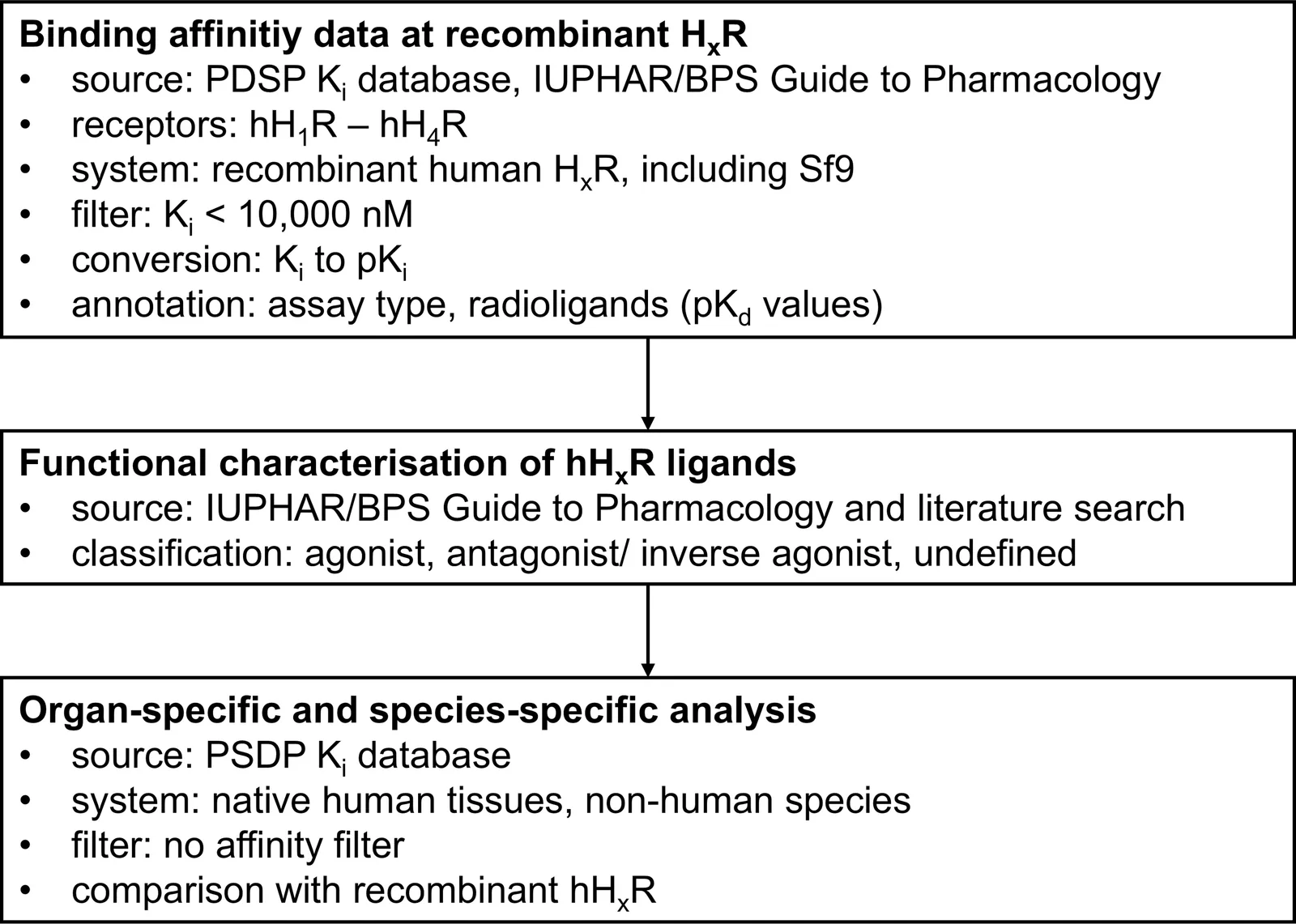
Flowchart of the methodological approach for the systematic analysis of H_x_R ligands.

In this analysis, it is specifically addressed how ligand binding affinities are distributed across the 4 hH_x_R, to what extent ligands are selective versus multireceptor active, how complete and consistent current pharmacological databases are with respect to hH_x_R ligands, and how binding affinities differ between recombinant human H_x_R (hH_x_R), native human tissues, and different species. Therefore, binding affinity data for ligands retrieved from recombinant cloned human and Sf9 H_x_R systems were extracted from the PDSP K_i_ database and complemented by available data from the IUPHAR/BPS Guide to Pharmacology (accessed January 2026), being transformed to pK_i_ values, and functional annotations were retrieved from the IUPHAR/BPS Guide to Pharmacology and complemented by literature search where necessary. Incomplete receptor coverage for many ligands reflects common experimental screening strategies, where compounds are often tested only on selected receptor subtypes, rather than inherent limitations of the databases themselves. Binding affinities obtained in native human tissues and in nonhuman species were compared with those derived from recombinant hH_x_R to assess context-dependent variability. In Fig. 1, a flowchart visualizes methodological considerations.

Using histamine receptors as a model system, this minireview illustrates how database-driven analysis can identify patterns, similarities, and knowledge gaps in receptor pharmacology and provides a structured basis for future experimental investigations and drug development.

## 2 Background

Histamine receptors have been investigated for several decades and represent early examples of clinically successful GPCR targets. Initial pharmacological characterization of hH_1_R and hH_2_R established histamine receptors as major therapeutic targets for allergic diseases and gastric acid-related disorders.^1^^,^^11^^,^^12^ Over time, molecular pharmacological studies expanded this framework by describing histamine receptor signaling properties, constitutive receptor activity, and functional behavior (including context-dependent ligand efficacy, including partial agonism and receptor state–dependent ligand behavior).^6^^,^^13^, ^14^, ^15^, ^16^

The subsequent identification and characterization of hH_3_R and hH_4_R broadened histamine receptor research toward CNS functions and immunological processes.^1^^,^^5^ The availability of subtype-selective ligands enabled more detailed investigation of receptor-specific pharmacology, whereas recombinant expression systems facilitated systematic profiling of ligand binding affinities and functional behavior across hH_x_R.^14^^,^^17^ More recently, hH_3_R antagonists have reached clinical application for narcolepsy, whereas hH_4_R ligands have primarily been explored in preclinical and clinical studies targeting inflammatory and immune-mediated diseases.^18^, ^19^, ^20^

In parallel with experimental developments, pharmacological databases have become increasingly important for organizing and integrating ligand binding data and functional characterization. Resources such as the PDSP K_i_ database and the IUPHAR/BPS Guide to Pharmacology provide curated and large-scale collections of receptor–ligand interactions that support comparative analyses across receptor families and ligand classes. These databases differ in scope, curation strategies and inclusion criteria, resulting in variable coverage of ligands and receptor targets.

Across the H_x_R family, ligand profiling has been performed unevenly. For many compounds, binding affinity data are available for only a subset, and functional characterization is not uniformly reported. In addition, binding affinities have been determined in different experimental systems, including recombinant systems, native human tissues, and nonhuman species, which provides heterogeneous and fragmented datasets of diverse experimental contexts.

## 3 Binding affinities and radioligands of hH_1_R–hH_4_R, for ligands retrieved from the PDSP K_i_ database

For the hH_x_R family (hH_1_R, hH_2_R, hH_3_R, and hH_4_R), 302 ligands with diverse functional behavior (agonists, antagonists, and inverse agonists) were extracted from the PDSP K_i_ database with binding affinities above pK_i_ 5 (K_i_ below 10,000 nM). Importantly, many of these ligands are not primarily developed as H_x_R–targeting drugs but have their main pharmacological activity at other targets. The included ligands comprise approved drugs, endogenous ligands, as well as experimental compounds. In Table 1,^6^^,^^21^ binding affinities are provided for the for ligands curated in the IUPHAR/BPS Guide to Pharmacology with an H_x_R target, whereas in Supplemental Table 1, a comprehensive overview about binding affinities for all ligands and recombinant hH_x_R are presented.

**Table 1:** Overview of ligands curated with an H_x_R target in the IUPHAR/BPS Guide of Pharmacology^6^ and their binding affinities (pK_i_) at hH_1_R–hH_4_R, retrieved from the PDSP K_i_ database^21^

Ligand Name	Ligand No.	Ligand Target (GPCRs)^6^	hH_1_R^21^		hH_2_R^21^		hH_3_R^21^		hH_4_R^21^	
			pK_i_ (Single Listing or Median)	pK_i_ Range	pK_i_ (Single Listing or Median)	pK_i_ Range	pK_i_ (Single Listing or Median)	pK_i_ Range	pK_i_ (Single Listing or Median)	pK_i_ Range
Cyproheptadine	**154**	**H_1_R**, *α*_1B_-AR, *α*_1A_-AR, 5-HT_7_R, *α*_1D_-AR, 5-HT_6_R	10.2		No data		No data		6.7	
ORG-5222	**233**	**5-HT_2A_R,** H_1_R, **D_2_R**, 5-HT_1D_R, 5-HT_1A_R, 5-HT_1B_R, 5-HT_1E_R	9.8		8.6		No data		No data	
Doxepin	**164**	H_1_R	9.8	0.6	0.0		Inactive		7.0	
Chlorpromazine	**135**	H_1_R, **5-HT_2A_R**, 5-HT_2C_R, D_4_R, 5-HT_6_R, 5-HT_7_R, *α*_2B_-AR, D_3_R, D_2_R, D_1_R, D_5_R, *α*_2C_-AR	9.7	1.5	6.8		Inactive		5.6	2.0
Promethazine	**242**	**H_1_R**	9.6		No data		No data		6.8	
Amitriptyline	**116**	**H_1_R**, *α*_1A_-AR, **M_4_R, M_2_R, M_3_R, M_1_R, M_5_R**, 5-HT_6_R, LPA_1_R	9.1	0.3	No data		Inactive		7.5	
Astemizole	**122**	**H_1_R**	9.1		No data		No data		No data	
Mizolastine	**215**	**H_1_R**	9.1		No data		No data		No data	
Triprolidine	**272**	**H_1_R**	9.0	0.6	No data		No data		No data	
Olanzapine	**232**	H_1_R, **5-HT_2A_R, D_2_R, 5-HT_2c_R**, 5-HT_6_R, 5-HT_1F_R, 5-HT_7_R, 5-HT_1B_R, 5-HT_1D_R, 5-HT_1A_R, 5-HT_1E_R	8.8	0.9	7.4		Inactive		Inactive	
Clozapine	**152**	H_1_R, 5-HT_2B_R, **5-HT_2A_R**, *α*_1A_-AR, 5-HT_2C_R, 5-HT_6_R, *α*_1B_-AR, *α*_1D_-AR, 5-HT_7_R, D_4_R, 5-HT_1F_R, D_1_R, 5-HT_1A_R, D_5_R, H_4_R, 5-HT_1D_R, 5-HT_1E_R, **D_2_R**, 5-HT_5A_R, 5-HT_1B_R, D_3_R, M_1_R	8.8	2.9	6.8		Inactive		6.3	0.6
Mepyramine	**207**	**H_1_R**	8.8	0.7	No data		No data		No data	
Trans-H2-PAT (−)	**267**	H_1_R	8.7	0.2	No data		No data		No data	
Chlorpheniramine	**134**	**H_1_R**	8.6	0.0	No data		No data		5.5	
Terfenadine	**261**	**H_1_R**	8.6	0.2	No data		No data		No data	
Levocetirizine	**198**	**H_1_R**	8.5		No data		No data		No data	
Trans-H2-PAT (±)	**269**	H_1_R	8.5	0.2	No data		No data		No data	
Loxapine	**201**	H_1_R, **D_2_R, D_4_R, 5-HT_2A_R, 5-HT_2C_R, D_3_R**, 5-HT_6_R	8.2	3.2	6.7		Inactive		5.3	
Cetirizine	**130**	**H_1_R**	8.2	0.2	No data		No data		No data	
Thiothixene	**265**	H_1_R, **5-HT_2A_R**	8.1	0.5	6.4		No data		No data	
Perphenazine	**237**	**D_2_R**, 5-HT_2A_R, H_1_R, 5-HT_7_R, 5-HT_6_R, 5-HT_2C_R	8.1		No data		inactive		Inactive	
Quetiapine	**244**	H_1_R, **D_2_R, 5-HT_2A_R,** 5-HT_1A_R, 5-HT_6_R, 5-HT_1E_R, 5-HT_1D_R, 5-HT_1F_R	8.1	0.7	No data		No data		No data	
Fexofenadine	**170**	**H_1_R**	8.0	0.0	No data		No data		No data	
Diphenhydramine	**162**	**H_1_R**	7.9	0.1	No data		No data		No data	
Fluspirilene	**174**	5-HT_2A_R, H_1_R, 5-HT_1A_R, 5-HT_1D_R, 5-HT_1E_R	7.9		No data		No data		No data	
Thioridazine	**264**	**5-HT_2A_R**, H_1_R, **5-HT_2C_R**, 5-HT_6_R, 5-HT_1A_R, 5-HT_7_R, D_1_R, D_5_R	7.8	0.1	6.9		Inactive		5.6	
9-OH-risperidone	**106**	5-HT_2A_R, 5-HT_1D_R, H_1_R, 5-HT_1B_R, 5-HT_1A_R, 5-HT_1E_R	7.7	0.8	6.9		No data		5.1	
Fluphenazine	**173**	**D_2_R**, 5-HT_7_R, D_5_R, D_1_R, H_1_R, 5-HT_2A_R, 5-HT_6_R	7.7	1.0	6.3		Inactive		Inactive	
Risperidone	**250**	**5-HT_2A_R, D_2_R**, *α*_1A_-AR, 5-HT_7_R, **5-HT_1D_R**, *α*_1B_-AR, H_1_R, 5-HT_2C_R, *α*_1D_-AR, *α*_2A_-AR, D_3_R, **5-HT_1B_R,** 5-HT_1A_R, 5-HT_1E_R, 5-HT_1F_R, 5-HT_6_R	7.6	0.7	6.9		Inactive		Inactive	
Aripiprazole	**120**	**D_2_R**, 5-HT_1A_R, 5-HT_2A_R, 5-HT_2C_R, H_1_R, 5-HT_1D_R, 5-HT_1B_R	7.5	0.1	No data		No data		No data	
*Trans*-H2-PAT (+)	**268**	H_1_R	7.4	0.2	No data		No data		No data	
Ziprasidone	**303**	**5-HT_2A_R,** 5-HT_1D_R**, D_2_R, 5-HT_2C_R,** 5-HT_1A_R, 5-HT_7_R, 5-HT_1B_R, H_1_R, 5-HT_1E_R	7.3	0.9	5.5		No data		No data	
Trifluoperazine	**271**	**D_2_R**, 5-HT_2A_R, D_4_R, H_1_R, 5-HT_2C_R	7.2		No data		No data		No data	
*Cis*-H2-PAT (+)	**145**	H_1_R	6.9	0.0	No data		No data		No data	
Sertindole	**254**	**5-HT_2A_R, 5-HT_2C_R, D_2_R, D_4_R, D_3_R,** 5-HT_1D_R, 5-HT_1B_R, H_1_R, 5-HT_1A_R, 5-HT_1E_R, 5-HT_1F_R	6.9	0.0	No data		No data		No data	
Loratidine	**200**	**H_1_R**	6.8		No data		No data		No data	
BU-E 47	**126**	H_1_R	6.6		No data		No data		No data	
Arpromidine	**121**	H_2_R, H_1_R	6.5		No data		No data		No data	
Dimethylhistaprodifen	**161**	H_1_R	6.4		No data		No data		No data	
Methylhistaprodifen	**209**	H_1_R	6.4		No data		No data		No data	
Pimozide	**239**	**D_2_R,** D_3_R**, 5-HT_2A_R,** 5-HT_1A_R, H_1_R, 5-HT_7_R, D_2_R, D_3_R, 5-HT_6_R	6.2		No data		No data		No data	
2-Methylhistamine	**81**	H_4_R	6.1		No data		No data		No data	
SR 147778	**260**	**CB_1_R,** CB_2_R	6.0		6.0		6.0		No data	
Histamine	**182**	**H_3_R, H_4_R, H_1_R, H_2_R**	5.9	2.6	7.8		8.3	0.7	7.8	3.1
Haloperidol	**180**	**D_4_R, D_2_R, D_3_R,** D_1_R, **5-HT_2A_R**, 5-HT_1D_R, 5-HT_7_R, D_5_R, 5-HT_1B_R, H_1_R, 5-HT_1A_R, *TASR10*	5.9	1.3	6.0		1.0		Inactive	
ABT-239	**112**	H_3_R, H_1_R, H_2_R	5.8	0.0	5.2	0.0	9.3	0.1	No data	
Ciproxifan	**143**	H_3_R, H_4_R, H_1_R, H_2_R	5.7		No data		7.2	0.0	5.7	0.0
Molindone	**216**	**5-HT_2A_R**, H_1_R	5.7		No data		No data		No data	
Clobenpropit	**151**	H_3_R, H_4_R, H_1_R, H_2_R	5.6		5.2		7.2	1.4	7.9	0.7
A-349821	**110**	H_3_R, H_1_R	5.6	0.0	No data		9.4		No data	
Pipamperone	**240**	5-HT_2A_R, 5-HT_1D_R, 5-HT_1B_R, 5-HT_1A_R, H_1_R	5.6		No data		No data		No data	
A-331440	**109**	H_3_R	5.5		No data		No data		No data	
Haldol	**179**	**D_4_R, D_2_R, D_3_R,** D_1_R, **5-HT_2A_R**, 5-HT_1D_R, 5-HT_7_R, D_5_R, 5-HT_1B_R, H_1_R, 5-HT_1A_R, *TASR10*	5.5		No data		No data		No data	
A-317920	**108**	H_3_R, H_1_R	5.4		No data		7.0		No data	
Proxyfan	**243**	H_3_R	No data		7.9		8.3		7.3	
Famotidine	**168**	**H_2_R**, *TAS2R31*	No data		7.8		No data		No data	
Tiotidine	**266**	H_2_R	No data		7.8		No data		No data	
Ranitidine	**248**	**H_2_R**	No data		7.1		No data		No data	
Impromidine	**191**	H_4_R, H_2_R, H_3_R, H_1_R	No data		6.6	1.3	8.3	0.4	7.7	0.3
Dimaprit	**159**	H_3_R, H_4_R	No data		6.5		6.1		6.4	
Cimetidine	**140**	**H_2_R**	No data		6.2		No data		No data	
Burimamide	**127**	H_3_R, H_4_R, H_2_R	No data		5.7	2.0	8.3	0.8	7.0	0.7
4-Methylhistamine	**96**	H_4_R	No data		5.4	2.2	No data		7.4	0.3
Amthamine	**119**	H_2_R	No data		5.2	0.1	No data		No data	
Iodoproxyfan	**193**	H_3_R	No data		No data		9.2		7.9	
Immepip	**188**	H_3_R, H_4_R	No data		No data		9.2	0.6	7.7	0.3
JNJ-5207852	**195**	H_3_R	No data		No data		9.2		No data	
Immethridine	**189**	H_3_R	No data		No data		9.1		6.6	
Thioperamide	**263**	H_3_R, H_4_R	No data		No data		9.0	0.5	7.3	0.7
N-methylhistamine	**228**	H_3_R, H_4_R	No data		No data		8.8	0.7	7.6	
R-*α*-methylhistamine	**246**	H_3_R, H_4_R	No data		No data		8.6	1.1	6.5	0.2
Imbutamine	**185**	H_3_R	No data		No data		8.4		8.0	
N-*α*-methylhistamine	**222**	H_4_R	No data		No data		8.4		6.5	
Imetit	**186**	H_3_R, H_4_R	No data		No data		8.3	0.8	8.2	3.1
Cipralisant	**142**	H_3_R	No data		No data		8.3		7.3	0.0
Impentamine	**190**	H_3_R	No data		No data		8.3	0.0	6.6	
Iodophenpropit	**192**	H_3_R, H_4_R	No data		No data		6.9	0.1	7.8	0.2
A-304121	**107**	H_3_R	No data		No data		6.1		No data	
JNJ 7777120	**194**	H_4_R	No data		No data		5.3		7.8	0.0

If duplicate values are available, the median is given and the range provided. The label “inactive” is assigned to ligands where available data report a binding affinity (K_i_) above 10,000 nM, whereas the label “no data” highlights that no binding affinity is available in the PDSP K_i_ database. Furthermore, all important targets, assigned by the IUPHAR/BPS Guide to Pharmacology for the human species and sorted by descending affinity, are provided, highlighted in bold if considered as the primary target of the ligand. Ligands (with the ligand no. in bold) are sorted in descending order from the highest binding affinity for the hH_1_R. A detailed overview about the binding affinity of hH_x_R for all analyzed ligands can be found in Supplemental Table 1.

Most K_i_ values included in this analysis are derived from radioligand binding assays, based on competitive displacement of radioactively labeled ligands by the test compound. Consistent with this, the compiled dataset shows that almost all annotated “hot ligands” correspond to tritiated [^3^H] or iodinated radioligands. Frequently used radioligands include [^3^H]mepyramine (synonym [^3^H]pyrilamine) for the hH_1_R, [^3^H]tiotidine for the hH_2_R, [^3^H]NAMH ([^3^H]-N-*α*-methylhistamine) as well as [^3^H]RAMH ([^3^H]-R-*α*-methylhistamine) for the hH_3_R, and [^3^H]histamine for the hH_4_R. [^3^H]NAMH, [^3^H]RAMH and [^3^H]histamine represent agonistic radioligands, whereas the remaining commonly used radioligands are antagonists/inverse agonists.^6^ In the absence of guanosine triphosphate (GTP), agonists generally display a higher affinity state, whereas the presence of GTP shifts receptors toward lower-affinity conformations.^22^ In contrast, antagonist binding is GTP-insensitive. Radioligand binding assays are typically performed using washed membrane preparations with little or no residual GTP.^23^ However, a small subset of entries is based on functional assays or lacks explicit methodological annotation (“undefined”). In Supplemental Table 2, an overview of the methodological procedure is provided for each ligand and binding affinity derived from the PDSP K_i_ database. In Supplemental Table 3, frequently used radioligands are characterized for hH_x_R.^6^

Notably, pK_d_ values, defined as the negative decadic logarithm of the equilibrium dissociation constant calculated from radioligand binding assays, can differ strongly between radioligands and hH_x_R subtypes.^14^ These values are highly relevant, because the K_d_ value of the radioligand directly reflects its affinity to the receptor and therefore its suitability for competition binding assays. Radioligands with low K_d_ values, corresponding to high pK_d_ values, are generally more suitable, as they provide a higher fraction of specific binding, a better signal-to-noise ratio and consequently a more precise determination of pK_i_ values for test compounds. In contrast, radioligands with high K_d_ values and high nonspecific binding are less suitable, because they require higher radioligand concentrations and lead to a less robust experimental assay. This is illustrated by [^3^H]tiotidine for the hH_2_R, which shows a comparably low affinity with a pK_d_ range between 7.7 and 8.7 and high nonspecific binding of approximately 5% to 60%,^6^^,^^14^ This limits assay precision and may explain its relatively infrequent use. In contrast, [^3^H]mepyramine for the hH_1_R shows higher affinity, with pK_d_ values of 8.4–9.1 and only 10%–15% nonspecific binding,^6^^,^^14^ supporting more reliable determination of pK_i_ values and explaining its frequent use in radioligand binding assays. Similar considerations apply to hH_3_R and hH_4_R radioligands, where [^3^H]NAMH, [^3^H]RAMH, and [^3^H]histamine are frequently used but differ in subtype selectivity and affinity, with reports of approximately 20%–30% nonspecific binding.^14^ In addition, receptor cooperativity may further influence measured affinities, since ligand binding can exhibit positive or negative cooperative interactions depending on receptor conformation, G protein coupling, and experimental system.^24^

Most ligands (*n* = 231) are only listed for one hH_x_R. However, this apparent selectivity must be interpreted with caution. For several ligands, particularly those with high affinity at hH_1_R, the binding to hH_x_R represents only a secondary pharmacological target (Table 1). For example, pleiotropic ligands such as multiple G protein coupled receptors (mGPCR)-antagonists, including chlorpromazine (**135**), fluphenazine (**173**), and fluspirilene (**174**), as well as nonselective monoamine reuptake inhibitors (NSMRI) such as amitriptyline (**116**) exhibit high affinity at hH_1_R but often display pleiotropic ligand binding behavior at a variety of GPCRs. Consequently, high affinity at a given hH_x_R does not necessarily imply pharmacological selectivity or therapeutic relevance for histamine receptor modulation, as seen for doxepin (**164**). Rather, these interactions may contribute to off-target effects, such as sedation or weight gain, for example, commonly associated with hH_1_R antagonism.^25^

There are multiple ligands that display binding affinities for several hH_x_R, for two receptors (*n* = 52), 3 receptors (*n* = 17), or all four hH_x_R (*n* = 2). Regarding the availability of binding affinities in ligands, the highest share is observed for the hH_1_R (46.8%), followed by hH_4_R (19.2%), hH_3_R (17.7%), and hH_2_R (16.2%) (Supplemental Table 4).

Regarding the distribution of the binding affinities, the highest average (median) pK_i_ is observed for the hH_3_R (7.7), followed by the hH_2_R (6.8), hH_1_R (6.6), and hH_4_R (6.7). A wide range of pK_i_ values is observed for the hH_1_R and hH_3_R, from 5.0–10.2 and 5.1–10.3, respectively. A comparable narrow range, although with a lower maximum binding affinity, is observed for the hH_2_R and hH_4_R, with 5.1–8.6 and 5.0–8.4, respectively (Supplemental Table 5). The similarities in median pK_i_ values across hH_1_R, hH_2_R and hH_4_R suggest a partially overlapping affinity, whereas the broader range observed for hH_1_R and hH_3_R indicates a greater pharmacological diversity of ligands. Importantly, the included ligands originate from different periods of experimental research, which may introduce bias, as the observed affinity distributions can reflect historical and methodological differences in ligand investigation and development.

**Fig. 2:**
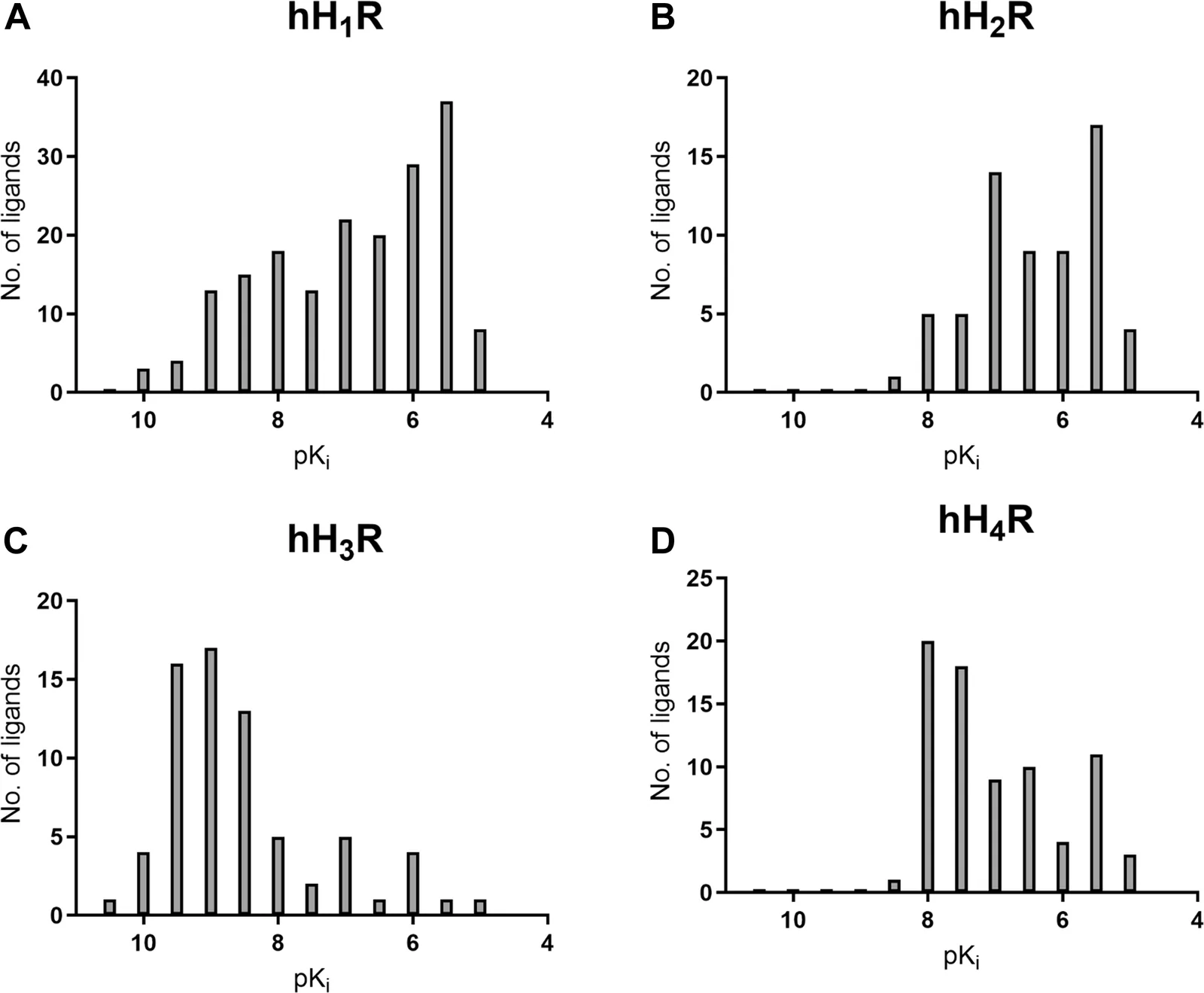
Distribution of binding affinities (pK_i_) of ligands at hH_x_R, retrieved from the PDSP K_i_ database.^21^ Separate panels are shown for (a) hH_1_R, (b) hH_2_R, (c) hH_3_R, and (d) hH_4_R. Gray-colored bars represent the absolute number of ligands per binding affinity value (pK_i_). In Fig. 3, a differentiated overview for agonists and antagonists/inverse agonists can be found. The visualized pK_i_ values can be found in Table 1 and Supplemental Table 1.

**Fig. 3:**
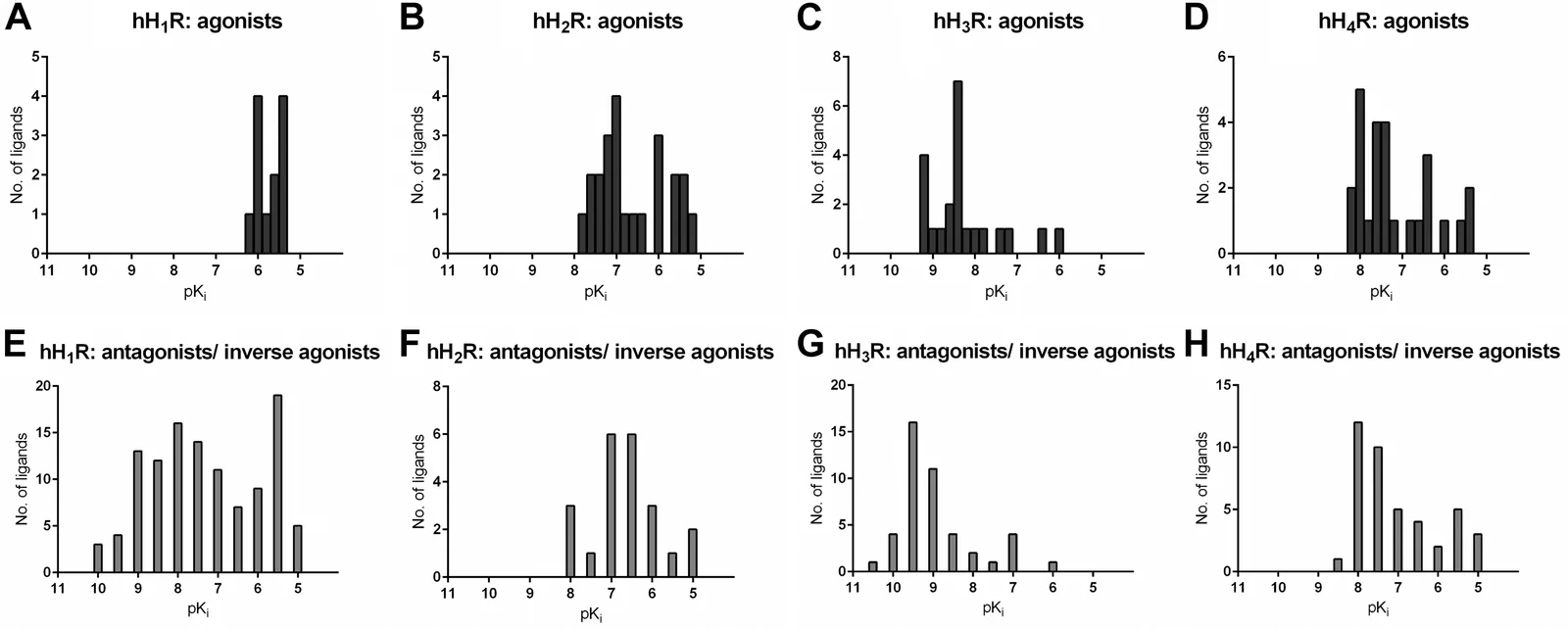
Distribution of binding affinities (pK_i_) of ligands at hH_x_R, retrieved from the PDSP K_i_ database,^21^ stratified by functional labels. The absolute number of ligands are depicted for the distribution of pK_i_ values for agonists (a–d) and antagonists/inverse agonists (e–h) at hH_1_R, hH_2_R, hH_3_R, and hH_4_R. Compared with Fig. 2, differences in affinity distributions between agonists and antagonists/inverse agonists across receptor subtypes are illustrated. Importantly, ligands that lack functional classification were excluded. The visualized pK_i_ values can be found in Table 1 and Supplemental Table 1.

The distribution of ligand binding affinities across the four hH_x_R is summarized in Fig. 2 and Supplemental Fig. 1. To account for substantial differences in data availability between receptor subtypes, binding affinities are shown both as absolute ligand counts and as relative percentage distributions. The broader distributions for hH_1_R and hH_3_R and the more confined distributions for hH_2_R and hH_4_R directly reflect the differences in pK_i_ ranges and median values. A more detailed visualization of the distribution of binding affinities is provided in Fig. 3, in which ligands are allocated by functional class (agonists vs antagonists/inverse agonists). Across all hH_x_R, agonists display narrower pK_i_ distributions, whereas antagonists and inverse agonists show broader and more heterogeneous distributions. This is particularly pronounced for the hH_1_R and hH_3_R, where antagonists/inverse agonists span a wide pK_i_ range, whereas agonists are more clustered within a narrowed range. Importantly, noncharacterized ligands are excluded, which may introduce some bias, resulting the need for a cautious interpretation of the pK_i_ distributions.

## 4 Characterization of ligands as agonists, antagonists, and inverse agonists at hH_x_R

For each ligand, a functional label was assigned if available. This includes a characterization of receptor-specific agonist, antagonist, and inverse agonist behavior at hH_x_R. Therefore, databases and corresponding literature were searched. Preferably, the annotation provided by the IUPHAR/BPS Guide to Pharmacology was referred to. If no functional label had been assigned to a specific ligand, the primary literature linked in the PDSP K_i_ database was analyzed. If binding behavior was not characterized there either, an additional literature search in PubMed and PubChem was conducted.

Although it was possible to assign agonistic or antagonistic behavior to many ligands, for 69 ligands, no information was available about their functional behavior on hH_x_R and therefore no label could be assigned. For 10 ligands, at least one hH_x_R could not be characterized, whereas information was found for other hH_x_R, allowing partial characterization of the respective ligand. For all remaining 223 ligands, it was possible to assign a functional label for all derived binding affinities.

Table 2^2^^,^^6^^,^^9^^,^^11^^,^^13^^,^^14^^,^^26^, ^27^, ^28^, ^29^, ^30^, ^31^, ^32^, ^33^, ^34^, ^35^, ^36^, ^37^, ^38^, ^39^, ^40^, ^41^, ^42^, ^43^, ^44^, ^45^, ^46^, ^47^, ^48^, ^49^, ^50^, ^51^, ^52^, ^53^, ^54^, ^55^, ^56^, ^57^, ^58^, ^59^, ^60^, ^61^, ^62^, ^63^, ^64^, ^65^, ^66^, ^67^, ^68^, ^69^, ^70^, ^71^, ^72^, ^73^, ^74^ presents the functional characterization of the selected ligands and hH_x_R. The distinction between antagonistic and inverse agonistic behavior is not always consistent. Many ligands historically assigned as antagonists may act as inverse agonists under experimental conditions with measurable constitutive receptor activity. Since the differentiation between antagonists and inverse agonists is assay-dependent and may not have been part of the original experimental design, this introduces some ambiguity into the functional classification. A similar limitation applies to the distinction between neutral antagonists and weak partial agonists. Depending on experimental conditions, ligands with low intrinsic efficacy may produce no detectable functional response and therefore appear neutral despite retaining weak agonistic activity.^75^ In general, the assignment of agonist properties is context-dependent and can vary with receptor expression level, signaling pathway, assay system, and species. As a result, functional labels should be interpreted as conditional descriptions within a specific experimental context rather than as static properties of a ligand.^2^

**Table 2:** Functional characterization of ligands as agonists, antagonist, and inverse agonists at the hH_1_R–hH_4_R for “active” binding

Ligand Name	Ligand No.	hH_1_R (Type/Action)	hH_2_R (Type/Action)	hH_3_R (Type/Action)	hH_4_R (Type/Action)
Histamine	**182**	Agonist/full agonist^6^	Agonist^26^	Agonist/full agonist^6^	Agonist/full agonist^6^
HIS	**181**	Agonist^27^	Agonist^27^		
UR-AK24	**276**	Agonist/partial agonist^27^	Agonist^27^		
UR-AK57	**282**	Agonist/partial agonist^27^	Agonist^27^		
UR-AK59	**283**	Agonist/partial agonist^27^	Agonist^27^		
UR-AK64	**285**	Agonist/partial agonist^27^	Agonist^27^		
UR-AK68	**287**	Agonist/partial agonist^27^	Agonist^27^		
2-{4-[4-(7-Methoxy-naphthalen-1-yl)piperazin-1-yl]-butyl}-4-methyl-2H-[12,4]triazine-3,5-dione	**79**	Agonist^26^			Agonist/full agonist^6^
8-Chloro-11-(4-isopropylpiperazin-1-yl)-5H-dibenzo[b,e][1,4]diazepine	**103**	Agonist^26^			
2-(2-Thiazolyl)ethanamine	**71**	Agonist^28^			
2-(3-Chlorophenyl)histamine	**72**	Agonist^28^			
2-(3-Iodophenyl)histamine	**73**	Agonist^28^			
Dimethylhistaprodifen	**161**	Agonist/full agonist^6^			
Methylhistaprodifen	**209**	Agonist/full agonist^6^			
Impromidine	**191**	Antagonist^6^	Agonist/full agonist^6^	Agonist/partial agonist^6^	Agonist/partial agonist^6^
Amthamine	**119**	Antagonist^6^	Agonist/full agonist^6^		
Amoxapine	**117**	Antagonist^26^	Agonist/full agonist^6^		
UR-AK22	**275**	Antagonist^27^	Agonist^27^		
UR-AK62	**284**	Antagonist^27^	Agonist^27^		
UR-AK67	**286**	Antagonist^27^	Agonist^27^		
Clobenpropit	**151**	Antagonist^6^	Antagonist^6^	Antagonist^6^	Agonist/partial agonist^6^
Ciproxifan	**143**	Antagonist^6^	Antagonist^6^	Antagonist^6^	Antagonist^6^
A-349821	**110**	Antagonist/inverse agonist^6^	Antagonist/inverse agonist^6^	Antagonist/inverse agonist^6^	
Loxapine	**201**	Antagonist^6^	Antagonist/inverse agonist^14^		Agonist^26^
N-desmethylclozapine	**226**	Antagonist/inverse agonist^14^	Antagonist/inverse agonist^14^		Agonist/partial agonist^14^
8-Chloro-11-[4-(1,1-dideutrioethyl)piperazin-1-yl]-5H-dibenzo[b,e][1,4]diazepine	**104**	Antagonist^6^	Antagonist^14^		Antagonist^14^
Chlorpromazine	**135**	Antagonist^6^	Antagonist/inverse agonist^14^		Inverse agonist^14^
Clozapine	**152**	Antagonist^6^	Antagonist/inverse agonist^14^		Antagonist^6^
Thioridazine	**264**	Antagonist^6^	Antagonist/inverse agonist^14^		Inverse agonist^14^
N-desalkylquetiapine	**225**	Antagonist^29^	Antagonist^29^		Antagonist^29^
Fluphenazine	**173**	Antagonist^6^	Antagonist/inverse agonist^14^		
Haloperidol	**180**	Antagonist^6^	Antagonist^14^		
Olanzapine	**232**	Antagonist^6^	Antagonist/inverse agonist^14^		
Risperidone	**250**	Antagonist^6^	Inverse agonist^14^		
Thiothixene	**265**	Antagonist^6^	Antagonist/inverse agonist^14^		
Zotepine	**304**	Antagonist^30^	Antagonist^31^		? (PDSP Certified Data)
9-OH-risperidone	**106**	Antagonist^6^		Antagonist^6^	
A-317920	**108**	Antagonist/inverse agonist^6^		Antagonist/inverse agonist^6^	
S-*α*-Methylhistamine	**114**	Antagonist^6^			Inverse agonist^14^
Promethazine	**242**	Antagonist^6^			Inverse agonist^14^
Mianserin	**212**	Inverse agonist^14^			Inverse agonist^14^
SYA 09 (+)	**13**	Antagonist^32^			
GBR-12909	**175**	Antagonist^33^			
cis-H2-PAT (±)	**146**	Antagonist^13^			
d-chlorpheniramine	**155**	Antagonist^13^			
Duloxetine	**165**	Antagonist^34^			
Burimamide	**127**	Antagonist^35^			
Nortryptyline	**229**	Antagonist^36^			
Trazodone	**270**	Antagonist^36^	? (PDSP Certified Data)		
(−)-(2RS,3aRS,12bSR)-2-N,N-Dimethylaminomethyl-11-fluoro-2,3,3a,12b-tetrahydrodibenzo[b,f]furo[2,3-d]tiepin	**1**	Antagonist^37^			
(+)-(2RS,3aRS,12bSR)-2-N,N-Dimethylaminomethyl-11-fluoro-2,3,3a,12b-tetrahydrodibenzo[b,f]furo[2,3-d]tiepin	**6**	Antagonist^37^			
(+)-7,8-Dimethoxy-3-[4-(2-methyl-3-phenyl-2(E)-propen-1-yl)piperazin-1-ylmethyl]-3a,4-dihydro-3H-[1]benzopyrano[4,3-c]isoxazole	**7**	Antagonist^37^			
(+)-7,8-Dimethoxy-3-[4-(3-methyl-3-phenyl-2(E)-propen-1-yl)piperazin-1-ylmethyl]-3a,4-dihydro-3H-[1]benzopyrano[4,3-c]isoxazole	**8**	Antagonist^37^			
(+)-7,8-Dimethoxy-3-[4-(3-phenyl-2(E)-propen-1-yl)piperazin-1-ylmethyl]-3a,4-dihydro-3H-[1]benzopyrano[4,3-c]isoxazole	**9**	Antagonist^37^			
(+)-7,8-Dimethoxy-3-[4-(naphthalen-2-ylmethyl)piperazin-1-ylmethyl]-3a,4-dihydro-3H-[1]benzopyrano[4,3-c]isoxazole	**10**	Antagonist^37^			
(2RS,3aRS,12bSR)-2-N,N-Dimethylaminomethyl-11-bromo-2,3,3a,12b-tetrahydrodibenzo[b,f]furo[2,3-d]oxepin	**18**	Antagonist^37^			
(2RS,3aRS,12bSR)-2-N,N-Dimethylaminomethyl-11-chloro-2,3,3a,12b-tetrahydrodibenzo[b,f]furo[2,3-d]oxepin	**19**	Antagonist^37^			
(2RS,3aRS,12bSR)-2-N,N-Dimethylaminomethyl-11-fluoro-2,3,3a,12b-tetrahydrodibenzo[b,f]furo[2,3-d]oxepin	**20**	Antagonist^37^			
(2RS,3aRS,12bSR)-2-N,N-Dimethylaminomethyl-11-fluoro-2,3,3a,12b-tetrahydrodibenzo[b,f]furo[2,3-d]tiepin	**21**	Antagonist^37^			
(2SR,3aRS,12bSR)-2-N,N-Dimethylaminomethyl-11-chloro-2,3,3a,12b-tetrahydrodibenzo[b,f]furo[2,3-d]oxepin	**22**	Antagonist^37^			
(2SR,3aRS,12bSR)-2-N,N-Dimethylaminomethyl-11-fluoro-2,3,3a,12b-tetrahydrodibenzo[b,f]furo[2,3-d]oxepin	**23**	Antagonist^37^			
(2SR,3aRS,12bSR)-2-N,N-Dimethylaminomethyl-11-fluoro-2,3,3a,12b-tetrahydrodibenzo[b,f]furo[2,3-d]tiepin	**24**	Antagonist^37^			
4-Methoxy-diphenidine	**95**	Antagonist^38^			
5-APT	**97**	Antagonist^38^			
Atomoxetine	**123**	Antagonist^38^			
Cetirizine	**130**	Antagonist^38^			
Cetirizine (+) isomer	**131**	Antagonist^38^			
CF3-5-APT	**132**	Antagonist^38^			
CF3-6-APT	**133**	Antagonist^38^			
CL-5-APT	**148**	Antagonist^38^			
CL-6-APT	**149**	Antagonist^38^			
OCH3-5-APT	**230**	Antagonist^38^			
OCH3-6-APT	**231**	Antagonist^38^			
Amselamine	**118**	Antagonist^6^			
Aripiprazole	**120**	Antagonist^6^			
ATR	**124**	Antagonist^6^			
Butaclamol (+)	**128**	Antagonist^6^			
Chlorpheniramine	**134**	Antagonist^6^			?^39^
*Cis*-H2-PAT (+)	**145**	Antagonist^6^			
Cyproheptadine	**154**	Antagonist^6^			?^39^
Diphenhydramine	**162**	Antagonist^6^			
Doxepin	**164**	Antagonist^6^			?^39^
Fexofenadine	**170**	Antagonist^6^			
Fluspirilene	**174**	Antagonist^6^			
Haldol	**179**	Antagonist^6^			
loratidine	**200**	Antagonist^6^			
Mizolastine	**215**	Antagonist^6^			
Molindone	**216**	Antagonist^6^			
ORG-5222	**233**	Antagonist^6^	?^40^		
Perphenazine	**237**	Antagonist^6^			
Pimozide	**239**	Antagonist^6^			
Pipamperone	**240**	Antagonist^6^			
Quetiapine	**244**	Antagonist^6^			
Sertindole	**254**	Antagonist^6^			
SR 147778	**260**	Antagonist^6^	?^41^	?^41^	
Terfenadine	**261**	Antagonist^6^			
*Trans*-H2-PAT(−)	**267**	Antagonist^6^			
*Trans*-H2-PAT(+)	**268**	Antagonist^6^			
*Trans*-H2-PAT(±)	**269**	Antagonist^6^			
Triflouperazine	**271**	Antagonist^6^			
Triprolidine	**272**	Antagonist^6^			
Ziprasidone	**303**	Antagonist^6^	? (PDSP Certified Data)		
Cimbi-772 (−)	**2**	Antagonist^42^			
Cimbi-775 (−)	**3**	Antagonist^42^			
Cimbi-772 (+)	**11**	Antagonist^42^			
Cimbi-775 (+)	**12**	Antagonist^42^			
Aminopotentidine	**115**	Antagonist^26^			
Ebastine	**166**	Antagonist^26^			
Terfenadine, R(+)	**262**	Antagonist^26^			
Raclopride	**245**	Antagonist^43^			
Ketanserin	**196**	Antagonist^44^			
Spiperone	**259**	Antagonist^45^			
Citalopram	**147**	Antagonist^46^			
Nefazodone	**227**	Antagonist^46^			
Sertraline	**255**	Antagonist^46^			
Escitalopram	**167**	Antagonist^47^			
Mirtazapine	**213**	Antagonist^48^			
cis-H2-PAT (−)	**144**	Antagonist/inverse agonist^13^			
NANM (−)	**4**	Antagonist/inverse agonist^49^			
Levocetirizine	**198**	Antagonist/inverse agonist^6^			
Mepyramide	**207**	Antagonist/inverse agonist^6^			
Desipramine	**156**	Inverse agonist^14^			
Imipramine	**187**	Inverse agonist^14^			
Cariprazine	**129**	Inverse agonist^50^			
Hydroxyzine, (R)	**184**	Inverse agonist^50^			
l-chlorpheniramine	**197**	Inverse agonist^50^			
ucb 29992, (R)	**273**	Inverse agonist^50^			
ucb 29993, (S)	**274**	Inverse agonist^50^			
Amitriptylin	**116**	Inverse agonist^11^	Agonist^26^		
Dimaprit	**159**		Agonist^26^	Agonist/full agonist^6^	Agonist/full agonist^6^
Br-6-APT	**125**		Agonist/full agonist^6^	Antagonist^6^	Antagonist^6^
4-{2-[2-(2-(R)-Methylpyrrolidin-1-yl)ethyl]benzofuran-5-ylamino}benzonitrile	**94**		Agonist^26^		Agonist/full agonist^6^
Sopromidine R (−)	**257**		Agonist^26^		
Sopromidine S (+)	**258**		Agonist^26^		
UR-AK26	**277**		Agonist^27^		
UR-AK41	**278**		Agonist^27^		
UR-AK46	**279**		Agonist^27^		
UR-AK49	**280**		Agonist^27^		
UR-AK51	**281**		Agonist^27^		
VUF4617	**290**		Antagonist^26^		Antagonist^26^
VUF4742	**296**		Antagonist^26^		Inverse agonist^26^
R-*α*-methylhistamine	**113**		Antagonist^26^		
Cimetidine	**140**		Antagonist^6^		
Famotidine	**168**		Antagonist^6^		
Ranitidine	**248**		Antagonist^6^		
Tiotidine	**266**		Antagonist^6^		
A-80426	**111**			Agonist^26^	Agonist^26^
ABT-239	**112**			Agonist^26^	Agonist^26^
Homohistamine	**183**			Agonist^26^	Agonist^26^
Imbutamine	**185**			Agonist/full agonist^6^	Agonist^26^
Imetit	**186**			Agonist/full agonist^6^	Agonist/full agonist^6^
Immepip	**188**			Agonist/full agonist^6^	Agonist/full agonist^6^
immethridine	**189**			Agonist/full agonist^6^	Agonist^26^
Iodoproxyfan	**193**			Agonist/full agonist^6^	Agonist/partial agonist^26^
Methimmepip	**208**			Agonist^9^	Agonist^26^
N-*α*-methylhistamine	**222**			Agonist^26^	Agonist/full agonist^6^
N-methylhistamine	**228**			Agonist/full agonist^6^	Agonist/full agonist
R-*α*-methylhistamine	**246**			Agonist/full agonist^6^	Agonist/full agonist
VUF8328	**302**			Agonist^26^	Agonist/partial agonist^26^
Cipralisant	**142**			Agonist^6^	Antagonist^51^
Impentamine	**190**			agonist^26^	Antagonist, agonist/full agonist^6^
GT 2331	**178**			Agonist^52^	
RAMH	**247**			Agonist^9^	
VUF5207	**300**			Agonist^9^	
VUF5300	**301**			Agonist^9^	
Proxyfan	**243**		?^52^	Antagonist/inverse agonist^6^	Agonist/partial agonist^26^
Iodophenpropit	**192**			Antagonist^6^	Antagonist^6^
Thioperamide	**263**			Antagonist^6^	Antagonist^6^
(3,5-Dinitropyridin-2-yl){2-[2-(2-(R)-methylpyrrolidin-1-yl)ethyl]benzofuran-5-ylmethyl}amine	**25**			Antagonist^53^	
(5-Bromopyrimidin-2-yl){2-[2-(2-(R)-methylpyrrolidin-1-yl)ethyl]benzofuran-5-ylmethyl}amine	**36**			Antagonist^53^	
(5-Ethylpyrimidin-2-yl){2-[2-(2-(R)-methylpyrrolidin-1-yl)ethyl]benzofuran-5-yl}amine	**38**			Antagonist^53^	
(5-Ethylpyrimidin-2-yl){2-[2-(2-(R)-methylpyrrolidin-1-yl)ethyl]benzofuran-5-ylmethyl}amine	**39**			Antagonist^53^	
(6-Chloropyridazin-3-yl){2-[2-(2-methylpyrrolidin-1-yl)ethyl]benzofuran-5-ylmethyl}amine	**45**			Antagonist^53^	
{2-[2-(2-(R)-Methylpyrrolidin-1-yl)ethyl]benzofuran-5-yl}(2-nitrophenyl)amine	**52**			antagonist^53^	
{2-[2-(2-(R)-Methylpyrrolidin-1-yl)ethyl]benzofuran-5-yl}(3-nitropyridin-2-yl)amine	**53**			Antagonist^53^	
{2-[2-(2-(R)-Methylpyrrolidin-1-yl)ethyl]benzofuran-5-yl}(5-nitropyridin-2-yl)amine	**54**			Antagonist^53^	
{2-[2-(2-(R)-Methylpyrrolidin-1-yl)ethyl]benzofuran-5-yl}(5-trifluoromethylpyridin-2-yl)amine	**55**			Antagonist^53^	
{2-[2-(2-(R)-Methylpyrrolidin-1-yl)ethyl]benzofuran-5-yl}pyrazin-2-ylamine	**56**			Antagonist^53^	
{2-[2-(2-(R)-Methylpyrrolidin-1-yl)ethyl]benzofuran-5-yl}pyrimidin-2-ylamine	**57**			Antagonist^53^	
{2-[2-(2-(R)-Methylpyrrolidin-1-yl)ethyl]benzofuran-5-yl}pyrimidin-5-ylamine	**58**			Antagonist^53^	
{2-[2-(2-(R)-Methylpyrrolidin-1-yl)ethyl]benzofuran-5-ylmethyl}(2-nitrophenyl)amine	**59**			Antagonist^53^	
{2-[2-(2-(R)-Methylpyrrolidin-1-yl)ethyl]benzofuran-5-ylmethyl}(3-nitropyridin-2-yl)amine	**60**			Antagonist^53^	
{2-[2-(2-(R)-Methylpyrrolidin-1-yl)ethyl]benzofuran-5-ylmethyl}(5-nitropyridin-2-yl)amine	**61**			Antagonist^53^	
{2-[2-(2-(R)-Methylpyrrolidin-1-yl)ethyl]benzofuran-5-ylmethyl}(5-nitrothiazol-2-yl)amine	**62**			Antagonist^53^	
{2-[2-(2-(R)-Methylpyrrolidin-1-yl)ethyl]benzofuran-5-ylmethyl}pyrazin-2-ylamine	**63**			Antagonist^53^	
{2-[2-(2-(R)-Methylpyrrolidin-1-yl)ethyl]benzofuran-5-ylmethyl}pyrimidin-2-ylamine	**64**			Antagonist^53^	
{2-[2-(2-(R)-Methylpyrrolidin-1-yl)ethyl]benzofuran-5-ylmethyl}pyrimidin-5-ylamine	**65**			Antagonist^53^	
{2-[2-(2-Methylpyrrolidin-1-yl)ethyl]benzofuran-5-ylmethyl}(4-nitrophenyl)amine	**66**			Antagonist^53^	
2-({2-[2-(2-(R)-Methylpyrrolidin-1-yl)ethyl]benzofuran-5-ylmethyl}amino)nicotinonitrile	**70**			Antagonist^53^	
2,2,2-Trifluoro-1-[4-({2-[2-(2-methylpyrrolidin-1-yl)ethyl]benzofuran-5-ylmethyl}amino)phenyl]ethanone	**75**			Antagonist^53^	
2-Methoxy-diphenidine	**80**			Antagonist^53^	
2-Methylhistamine	**81**			Antagonist^53^	
3-({2-[2-(2-(R)-Methylpyrrolidin-1-yl)ethyl]benzofuran-5-ylmethyl}amino)pyrazine-2-carbonitrile	**83**			Antagonist^53^	
3-Aminofluoranthene	**86**			Antagonist^53^	
3-Methoxy-diphenidine	**87**			Antagonist^54^	
4-[3-(4-Chlorophenyl)-3-hydroxy-8-azabicyclo[3.2.1]oct-8-yl]-1-(4-fluorophenyl)-1-butanone	**92**			Antagonist^53^	
4-methylhistamine	**96**			Antagonist^53^	
6-({2-[2-(2-(R)-Methylpyrrolidin-1-yl)ethyl]benzofuran-5-ylmethyl}amino)nicotinonitrile	**98**			Antagonist^53^	
8R-lisuride	**105**			Antagonist^6^	
A-304121	**107**			Antagonist^6^	
JNJ-5207852	**195**			Antagonist^6^	
VUF4903	**297**			Antagonist^9^	
VUF4904	**298**			Antagonist^9^	
VUF5202	**299**			Antagonist^9^	
VUF4681	**291**				Agonist/partial agonist^26^
VUF4682	**292**				Agonist/partial agonist^26^
VUF4683	**293**				Agonist/partial agonist^26^
VUF4686	**294**				Agonist/partial agonist^26^
VUF4687	**295**				Agonist/partial agonist^26^
(1H-Indol-2-yl)-(4-methyl-piperazin-1-yl)-methanone	**15**				Antagonist^55^
(1H-Indol-2-yl)-(4-phenethyl-piperazin-1-yl)-methanone	**16**				Antagonist^55^
(1H-Indol-2-yl)-(piperazin-1-yl)-methanone	**17**				Antagonist^55^
(4-Bromo-1H-indol-2-yl)-(4-methyl-piperazin-1-yl)-methanone	**29**				Antagonist^55^
(4-Ethyl-piperazin-1-yl)-(1H-indol-2-yl)-methanone	**30**				Antagonist^55^
(5,7-Dichloro-1H-indol-2-yl)-(4-methyl-piperazin-1-yl)-methanone	**31**				Antagonist^55^
(5,7-Difluoro-1H-indol-2-yl)-(4-methyl-piperazin-1-yl)-methanone	**32**				Antagonist^55^
(5,7-Dimethyl-1H-indol-2-yl)-(4-methyl-piperazin-1-yl)-methanone	**33**				Antagonist^55^
(5-Amino-1H-indol-2-yl)-(4-methyl-piperazin-1-yl)-methanone	**34**				Antagonist^55^
(5-Bromo-1H-indol-2-yl)-(4-methyl-piperazin-1-yl)-methanone	**35**				Antagonist^55^
(5-Chloro-1H-indol-2-yl)-(4-methyl-piperazin-1-yl)-methanone	**37**				Antagonist^55^
(5-Fluoro-1H-indol-2-yl)-(4-methyl-piperazin-1-yl)-methanone	**40**				Antagonist^55^
(5-Hydroxy-1H-indol-2-yl)-(4-methyl-piperazin-1-yl)-methanone	**41**				Antagonist^55^
(5-Methoxy-1H-indol-2-yl)-(4-methyl-piperazin-1-yl)-methanone	**42**				Antagonist^55^
(5-Methyl-1H-indol-2-yl)-(4-methyl-piperazin-1-yl)-methanone	**43**				Antagonist^55^
(6-Bromo-1H-indol-2-yl)-(4-methyl-piperazin-1-yl)-methanone	**44**				Antagonist^55^
(7-Amino-1H-indol-2-yl)-(4-methyl-piperazin-1-yl)-methanone	**46**				Antagonist^55^
(7-Bromo-1H-indol-2-yl)-(4-methyl-piperazin-1-yl)-methanone	**47**				Antagonist^55^
(7-Chloro-1H-indol-2-yl)-(4-methyl-piperazin-1-yl)-methanone	**48**				Antagonist^55^
(7-Methyl-1H-indol-2-yl)-(4-methyl-piperazin-1-yl)-methanone	**49**				Antagonist^55^
+-(1H-Indol-2-yl)-(3-methyl-piperazin-1-yl)-methanone	**67**				Antagonist^55^
JNJ 7777120	**194**			?^26^	Antagonist^6^
MRS5698	**217**				Antagonist^56^
Pheniramine	**238**				Inverse agonist^57^
VUF4614	**288**				Antagonist^26^
VUF4616	**289**				Antagonist^26^
SYA 09 (−)	**5**	?^32^			
4-(8-Chloro-5H-dibenzo[b,e][1,4]diazepin-11-yl)-1-ethylpiperazine N-oxide	**90**	?^32^			
4-(8-Chloro-5H-dibenzo[b,e][1,4]diazepin-11-yl)-1-isopropylpiperazine N-oxide	**91**	?^32^			
A-331440	**109**	?^58^			
S-34324	**252**	?^58^			
[5-Fluoro-2-methyl-1-(4-methanesulfonyl-benzenesulfonyl)-1H-indol-3-yl]-acetic acid	**51**	?^59^	?^59^	?^59^	
Arpromidine	**121**	?^34^			
11-(Piperazin-1-yl)-5H-dibenzo[b,e][1,4]diazepine	**69**	?^60^			
4-({2-[2-(2-(R)-Methylpyrrolidin-1-yl)ethyl]benzofuran-5-ylmethyl}amino)pyridine-2,6-dicarbonitrile	**88**	?^60^			
4-(3-Piperidin-1-yl-propoxy)-benzonitrile	**89**	?^60^			
6-Methyl-2-{2-[2-(2-(R)-methylpyrrolidin-1-yl)ethyl]benzofuran-5-ylamino}nicotinonitrile	**100**	?^60^			
7-OH-DPAT	**101**	?^60^			
8-Chloro-11-(4-ethylpiperazin-1-yl)-5H-dibenzo[b,e][1,4]diazepine	**102**	?^60^			
Desmethylcitalopram	**157**	?^61^			
Desmethyldesipramine	**158**	?^61^	?^61^		
(±)-BMY-1402	**14**	?^38^			
(R)-Chlopheniramine	**50**	?^38^			
2-(Dimethylamino)tetralin	**74**	?^38^			
3-(Dimethylamino)fluoranthene	**84**	?^38^			
6-APT	**99**	?^38^			
Astemizole	**122**	?^38^			
BU-E 47	**126**	?^38^			
Flupenthixol, *α*	**171**	?^38^			
Flupenthixol, *β*	**172**	?^38^			
Loperamide	**199**	?^38^			
Mazindol	**202**	?^38^			
Methysergide	**210**	?^38^			
Rimcazole	**249**	?^38^			
SCH 23390	**253**	?^38^			
SKF 38393	**256**	?^38^			
GT 2016	**176**	?^62^	?^62^		?^62^
Cimbi-712	**136**	?^42^			
Cimbi-717	**137**	?^42^			
Cimbi-772	**138**	?^42^			
Cimbi-775	**139**	?^42^			
Dimethylamylamine	**160**	?^63^			
Naphyrone	**224**	?^63^	?^63^		
mCPP	**203**	?^64^			
Cocaine	**153**	? (PDSP Certified Data)			
pdspTestCompound2	**234**	? (PDSP Certified Data)			
pdspTestCompound2	**235**	? (PDSP Certified Data)			
pdspTestCompound3	**236**	? (PDSP Certified Data)			
RWAY (−)	**251**	? (PDSP Certified Data)	? (PDSP Certified Data)		
MDL 100907	**204**	?^65^			
MDL 11,939	**205**	?^65^			
2-{4-[4-(3-Methoxyphenyl)-1-piperazinyl]butyl}-4-methyl-1,2,4-triazine-3,5(2H,4H)-dione	**76**	?^66^	?^66^		
2-{4-[4-(3-Methoxyphenyl)-1-piperazinyl]butyl}-4-methyl-124-triazine-35(2H4H)-dione	**77**	?^66^	? 66^2^		
FECIMBI-36	**169**	?^66^	?^66^		
MDMA	**206**	?^67^			
1-(3-Methoxy-7,12-dihydro-12-tetraphenyl)methanamine	**68**	?^68^			
N-(9,10-Dihydro-9-anthracenylmethyl)-2-phenylethanamine	**219**	?^68^			
N-(9,10-Dihydro-9-anthracenylmethyl)-3-phenyl-1-propanamine	**220**	?^68^			
N-(9,10-Dihydro-9-anthracenylmethyl)-4-phenyl-1-butanamine	**221**	?^68^			
MRS5979	**218**	?^69^			
2-{4-[4-(7-Methoxy-naphthalen-1-yl)piperazin-1-yl]-butyl}-4-methyl-2H-[1,2,4]triazine-3,5-dione	**78**	?^70^			
3-({2-[2-(2-(R)-Methylpyrrolidin-1-yl)ethyl]benzofuran-5-ylmethyl}amino)benzonitrile	**82**	?^70^			
3-{2-[2-(2-(R)-Methylpyrrolidin-1-yl)ethyl]benzofuran-5-ylamino}benzonitrile	**85**	?^70^			
4-[5-trans-hydroxy-5-cis-(4′-chlorophenyl)-2-azabicyclo[3.2.1]octanyl]-4″-fluorobutyrophenone	**93**	?^70^			
Diphenidine	**163**	?^70^			
(3S,4S,6aS,9S,10S,12aS)-4,10-bis(4-(3-(dimethylamino)prop-1-ynyl)phenyl)-3,9-dimethyloctahydrodipyrido[1,2-a:1′,2′-d]pyrazine-6,12(6aH,12aH)-dione	**26**				?^71^
(3S4S6aS9S10S12aS)-4-(2-bromophenyl)-10-(4-(3-(dimethylamino)prop-1-ynyl)phenyl)-39-dimethyloctahydrodipyrido[12-a:1′2′-d]pyrazine-612(6aH12aH)-dione	**27**		?^71^		
(3S4S6aS9S10S12aS)-410-bis(4-(3-(dimethylamino)prop-1-ynyl)phenyl)-39-dimethyloctahydrodipyrido[12-a:1′2′-d]pyrazine-612(6aH12aH)-dione	**28**		?^71^		
Cinnarizine	**141**				?^39^
Clemizole	**150**				?^39^
GT 2227	**177**		?^72^		
MH.MZ	**211**		?^73^		
Mitragynine	**214**			? (PDSP Certified Data)	
Nantenin	**223**		?^74^		
Pramipexole	**241**		? (PDSP Certified Data)		

“?” stands for noncharacterized and therefore uncertain behavior in the literature. Agonists are green-colored, and antagonists and inverse agonists are orange-colored. Ligand no. are highlighted in bold. Ligands are sorted by agonistic behavior at the hH_1_R.

## 5 Variation of recombinant hH_x_R binding affinities: Deviation in replicate values and comparison with the IUPHAR/BPS Guide to Pharmacology

When comparing the listed ligands in the PDSP K_i_ database with the ligand search in the IUPHAR/BPS Guide to Pharmacology, the IUPHAR/BPS database appears incomplete, since only selective ligands and hH_x_R targets are included. Of the analyzed ligands of the PDSP K_i_ database with binding affinities (K_i_) below 10,000 nM, only 48 ligands are listed with all hH_x_R in the IUPHAR/BPS Guide to Pharmacology. From the total number of 302 extracted ligands, 254 ligands are not fully represented across all hH_x_R in the IUPHAR/BPS Guide to Pharmacology. This includes the listing of some, but not all, hH_x_R (*n* = 31), the respective ligand being listed but without a hH_x_R target (*n* = 40), or no listing of the ligand at all (*n* = 183) (Supplemental Table 5).

Regarding the included primary literature, there are both similarities and differences (Supplemental Table 6). For 15 publications, both databases include binding affinity from similar literature.^9^^,^^13^^,^^25^, ^26^, ^27^, ^28^^,^^30^^,^^38^^,^^51^^,^^54^^,^^62^^,^^76^, ^77^, ^78^, ^79^ However, 45 primary sources are only included in the PDSP K_i_ database,^21^^,^^29^^,^^32^^,^^34^, ^35^, ^36^^,^^39^^,^^41^, ^42^, ^43^^,^^45^^,^^46^^,^^48^^,^^51^, ^52^, ^53^^,^^55^^,^^58^, ^59^, ^60^, ^61^^,^^63^^,^^66^, ^67^, ^68^, ^69^, ^70^, ^71^, ^72^, ^73^, ^74^^,^^80^, ^81^, ^82^, ^83^, ^84^, ^85^, ^86^, ^87^, ^88^, ^89^, ^90^, ^91^ whereas 25 sources are only cited in the IUPHAR/BPS Guide to Pharmacology^1^^,^^51^^,^^92^, ^93^, ^94^, ^95^, ^96^, ^97^, ^98^, ^99^, ^100^, ^101^, ^102^, ^103^, ^104^, ^105^, ^106^, ^107^, ^108^, ^109^, ^110^, ^111^, ^112^, ^113^ but are not included in the selected ligand binding affinities of the PDSP K_i_ database. This may be because of that the literature solely included in the PDSP K_i_ database partly results from the inclusion of additional ligands not listed as hH_x_R targets in the IUPHAR/BPS Guide to Pharmacology, whereas some literature solely available in the IUPHAR/BPS Guide to Pharmacology may have been excluded during ligand selection from the PDSP K_i_ database (eg, K_i_ > 10,000 nM, no recombinant human system or undefined).

Notably, in both databases, most binding affinities were retrieved from published literature, with only 49 binding affinities (6.6% of inclusions) in the PDSP K_i_ database and one binding affinity (0.7% of inclusions) in the IUPHAR/BPS Guide to Pharmacology lacking a publication. The IUPHAR/BPS Guide to Pharmacology often considers only one reference for the characterization and definition of pK_i_ values (eg, iodoproxyfan (**193**) and cetirizine (**130**)), whereas the PDSP K_i_ database includes several sources for binding affinities, whenever available. However, no apparent bias was found in the selection of references in the IUPHAR/BPS Guide to Pharmacology. Examining the predominantly referenced literature in the IUPHAR/BPS Guide to Pharmacology shows that the same publication also contributes a high number of binding affinity entries in the PDSP K_i_ database.^13^^,^^25^, ^26^, ^27^^,^^54^^,^^76^^,^^78^

To assess the completeness of both databases, a representative literature search on PubMed was performed and the listed results were compared with the primary literature included in the respective databases (Supplemental Table 7). For this, important ligands for the hH_x_R were included: loratadine (**200**) for hH_1_R, ranitidine (**248**) for hH_2_R, thioperamide (**263**) for hH_3_R, and JNJ 7777120 (**194**) for hH_4_R. For loratadine (**200**) at the hH_1_R, one similar result is included, although 2 different references are cited that refer to the same experiment,^26^^,^^114^ whereas one additional publication^96^ is only included in the IUPHAR/BPS Guide to Pharmacology and not in the PDSP K_i_ database. For ranitidine (**248**) at the hH_2_R, one experiment (reported in 2 references)^26^^,^^113^ is captured in both databases, whereas one publication^115^ is not included in either database. For thioperamide (**263**) at the hH_3_R, three publications^9^^,^^76^^,^^79^ are included in both databases, whereas 3 publications are not captured by either database,^116^, ^117^, ^118^ and four publications each are uniquely represented in the PDSP K_i_ database^5^^,^^26^^,^^55^ or the IUPHAR/BPS Guide to Pharmacology.^51^^,^^105^, ^106^, ^107^ For JNJ 7777120 (**194**) at the hH_4_R, one publication^26^ is included in both databases, whereas 5 publications^119^, ^120^, ^121^, ^122^, ^123^ identified in the literature search are not represented in either database, and 2 publications are exclusively included in the IUPHAR/BPS Guide to Pharmacology.^102^^,^^104^ Overall, this comparison indicates that neither database captures completely the available literature, and that some published binding affinity data is not represented in either resource. Importantly, the overlap between databases is limited, with each database containing unique references that are absent in the other.

Regarding pK_i_ values stated by the IUPHAR/BPS Guide to Pharmacology, only the range for thioperamide (**263**) on hH_3_R is fully captured (7.1–7.7), whereas narrower ranges are reported compared with the literature for JNJ 7777120 (**194**) at the hH_4_R (7.8–8.3 vs 7.5–8.4 in the literature). For loratadine (**200**) at the hH_1_R and ranitidine (**248**) at the hH_2_R, only a single value is provided in the IUPHAR/BPS Guide to Pharmacology, although a broader range is observed in the literature (loratadine 6.8–7.5 vs 7.4; ranitidine 6.7–7.1 vs 7.1). These findings suggest that database-reported affinity values may not fully reflect the variability observed across experimental studies, and instead represent a subset or consensus selection of available data. Consequently, apparent “missing data” may in part reflect incomplete database coverage rather than a lack of experimental evidence.

These differences can be partly explained by the scope of the databases. The IUPHAR/BPS Guide to Pharmacology “is an open-access database of expert-curated, high-quality pharmacological data […] for pharmacological targets and their recommended experimental ligands” and includes 13,260 ligands.^6^ The scope of the database is “to include major areas of interest to pharmacology” and “to provide information on all the targets of currently licensed drugs as well as other potential targets of interest.”^6^

The PDSP K_i_ database “serves as a data warehouse for published and internally derived K_i_, or affinity, values for a large number of drugs and drug candidates at […] G protein coupled receptors”^21^ and includes 220,000 ligands.^124^ In contrast to the expert-curated IUPHAR/BPS Guide to Pharmacology, the PDSP K_i_ database aims to provide a more complete overview of recently available receptor affinities. The relevance of the PDSP K_i_ database for pharmacological research is further supported by its use in diverse scientific research (Table 3).^17^^,^^124^, ^125^, ^126^, ^127^, ^128^ Previous studies have used PDSP K_i_ data for large-scale ligand profiling and database integration,^124^^,^^125^ receptor-based reclassification of clinically used drugs,^17^ prediction of adverse-effect profiles and drug repurposing opportunities,^126^ as well as quantitative structure-activity relationship modeling and virtual screening approaches for ligand discovery.^127^^,^^128^ These studies illustrate that PDSP K_i_ data are valuable for both investigative and comparative analyses that integrate binding affinities across multiple receptors and ligand classes.

**Table 3:** Overview about available literature about the use of the PDSP K_i_ database in scientific research

Reference	Study Scope	Use of PDSP K_i_ Database	Key Information/Relevance
Herrera et al^124^	Release of new functions in the GPCRdb platform	Integration of PDSP K_i_ ligand data into GPCRdb ligand search and analysis tools	GPCRdb provides access to ∼220,000 ligands aggregated from multiple sources, including PDSP K_i_, enabling large-scale ligand profiling and receptor comparison
Pándy-Szekeres et al^125^	Major update of PDSP K_i_ database (2023 release)	Compilation of consistently determined binding affinities for psychoactive, endogenous, tool, and drug ligands	Highlights the importance of PDSP K_i_ as a curated resource for quantitative affinity data across GPCR targets
Salvi et al^17^	Receptor binding profiles of antidepressants (H_1_R, 5-HT_2c_R M_3_R, *α*_1A_R)	Systematic extraction of receptor affinities to evaluate pharmacodynamic effects	Demonstrated significant associations between antidepressant H_1_R affinity and weight gain, supporting receptor-based reclassification of drugs
Baker et al^126^	Identification of serotonin 5-HT_6_R binders and nonbindres	Use of PDSP K_i_ data to relate binding affinities to adverse-effect profiles	Adverse-effect patterns can predict unknown receptor activities, supporting drug repurposing opportunities
Luo et al^127^	Virtual screening and binding activity analysis of 5-HT_1A_R ligands	PDSP K_i_ binding data used to validate QSAR-based screening hits	Identification of novel 5-HT_1A_R active compounds with potential as anxiolytic or antipsychotic candidates
Wang et al^128^	Modeling ligand affinity and subtype selectivity for 5-HT_1E_R and 5-HT_1F_R receptors	PDSP K_i_ affinity values applied for QSAR model development	Predictive QSAR models support the design of novel subtype-selective GPCR ligands

QSAR, quantitative structure-activity relationship.

Besides the varying ligand and literature listings, both the PDSP K_i_ database and the IUPHAR/BPS Guide to Pharmacology contain a range of binding affinities in duplicates, as well as deviations between the pK_i_ values listed in these 2 databases. Here, “deviation in pK_i_” refers to the absolute numerical difference between 2 compared binding affinity values (|pK_i1_ − pK_i2_|). The range of pK_i_ values is for the hH_1_R 0.0–3.15, for the hH_2_R 0.0–2.2, for the hH_3_R 0.0–1.4, and for the hH_4_R 0.0–3.4. Comparing the pK_i_ values between the 2 databases, the variation is for the hH_1_R 0.0–3.4, for the hH_2_R 0.0–1.2, and for the hH_3_R and hH_4_R 0.0–1.7 (Supplemental Table 8).

The observed strong variations in binding affinities within the same receptor type and ligand (Supplemental Table 1) have already been reported previously. This can be explained by methodological variations and technical limitations, which complicate the determination and comparison of exact binding affinities.^2^^,^^11^ These sources of variability may include the use of different radiolabeled ligands for competitive binding assays, differences in assay design, and experimental conditions affecting receptor states. In addition, receptor-specific factors such as interactions with G proteins, nucleotide-dependent modulation, and shifts in the equilibrium between active and inactive receptor conformations may influence measured K_i_ values.^8^^,^^129^^,^^130^ Furthermore, receptor oligomerization can introduce cooperative binding effects (positive or negative cooperativity).^131^^,^^132^ Experimental systems also contribute substantially to variability. Differences in membrane composition, including phospholipid environment and cholesterol content between cell types,^133^ can modulate receptor conformation and ligand access. In recombinant expression systems such as Sf9 cells, incomplete glycosylation and other post-translational modifications^134^ may further affect receptor structure and ligand binding behavior. Finally, species-dependent differences in primary amino acid sequence,^107^ as well as the existence of receptor splice variants,^106^ may contribute to variability across datasets and experimental systems.

## 6 Affinity and selectivity patterns of ligands across hH_x_R

Ligands acting on hH_x_R differ in both binding affinity (pK_i_) and functional behavior (agonist, antagonist, and inverse agonist). A distinction can be made between selective and nonselective ligands, as well as between uniform agonist/antagonist behavior and switching behavior across receptor subtypes. This classification represents a simplified categorization of ligand efficacy by binding affinity (K_i_), which does not capture ligand efficacy or potency. In particular, for agonists, binding affinity alone provides only limited insight into pharmacological activity, as highly efficacious agonists may exhibit low affinity, whereas high affinity ligands may display only partial efficacy. The patterns described here primarily emerge from the integration of PDSP K_i_ binding data with IUPHAR/BPS functional annotations (Table 2).

Selective ligands have a high affinity at one particular hH_x_R but low affinity at others. However, selectivity within hH_x_R does not necessarily correspond to overall target selectivity. Many ligands with high affinity at hH_x_R also interact with other GPCRs, often with equal or higher affinity at further targets (Table 1).

For hH_1_R, both selective agonists as well as antagonists/inverse agonists are observed. Examples of selective agonists include methylhistaprodifen (**161**)^135^ and 8R-lisuride (**105**).^10^ Notably, there is a high number of selective antagonists/inverse agonists.^1^ Many hH_1_R antagonists are long-term established drugs, such as diphenhydramine (**162**), particularly used for allergic conditions,^12^ whereas hH_1_R antagonism contributes to weight gain.^25^

For hH_2_R, selective agonists as well as antagonists occur, for example, amthamine (**119**) as a selective agonist and famotidine (**168**) as an antagonist/inverse agonist.^115^^,^^136^ hH_2_R antagonists are clinically used for gastric acid regulation, whereas hH_2_R agonists may play a role in cardiac stimulation.^137^^,^^138^

For hH_3_R, selective agonists as well as antagonists are observed, although antagonists are more common. Examples include R-*α*-methylhistamine (**246**) as a selective agonist and A-331440 (**109**) as a selective antagonist].^1^^,^^9^ hH_3_R antagonists are of relevance for CNS-related conditions, with approval for the treatment of narcolepsy^19^ and potential in other CNS diseases.^139^ hH_3_R agonists influence energy homeostasis with a potential role in obesity and diabetes treatment.^140^

For hH_4_R, only a limited number of selective agonists or antagonists are identified, such as VUF4681 (**291**) as an agonist and VUF4616 (**289**) as an antagonist.^26^ The hH_4_R receptor remains under investigation, particularly for its role in inflammatory conditions.^1^^,^^141^ However, clinical development has so far not resulted in approved therapeutic applications.^18^^,^^20^

Although it is likely that some ligands appear to be highly selective, this interpretation has to be made cautiously. For many ligands, binding affinities are only available for 1 or 2 hH_x_R, whereas data for the remaining receptors are often missing. Consequently, the absence of reported binding does not allow discrimination between true selectivity or incomplete experimental characterization.

Multireceptor affinity is also common. Although many ligands act on at least 2 hH_x_R, some ligands have affinity at all 4 hH_x_R. These ligands can further be distinguished by uniform receptor activity (either agonism or antagonism across subtypes) or by switching behavior, meaning agonism at 1 receptor, whereas antagonism at another.

**Fig. 4:**
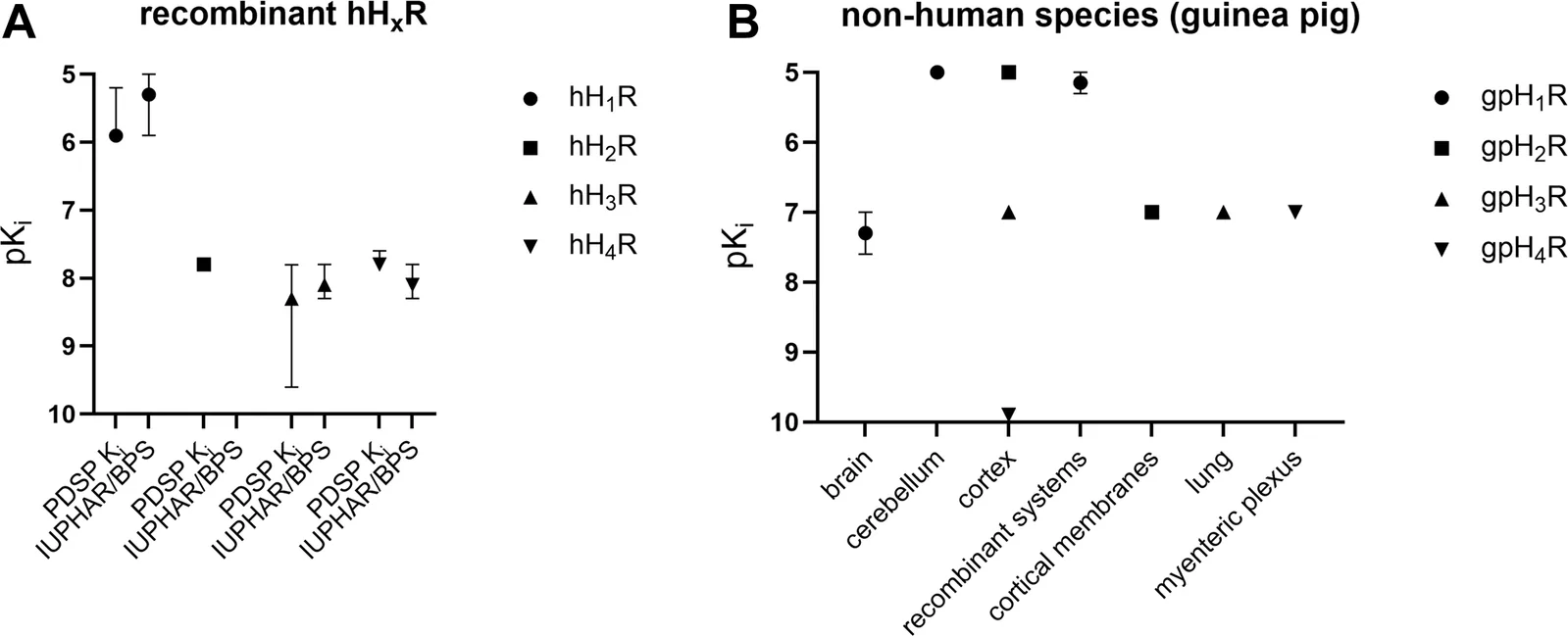
Binding affinities of histamine (**182**) throughout H_x_R systems. pK_i_ values across recombinant hH_x_R and gpH_x_R tissues were retrieved from the PDSP K_i_ database^21^ and the IUPHAR/BPS Guide to Pharmacology.^6^ Binding affinities are depicted for (A) recombinant hH_x_R, and (B) gpH_x_R, including native tissues and recombinant gpH_x_R. Histamine (**182**) shows pronounced receptor- and tissue-dependent variability, particularly for hH_1_R, hH_3_R, gpH_1_R, and gpH_4_R. Data points represent the median pK_i_ values, and error bars indicate variability where available.

Histamine (**182**) is the only ligand displaying full agonism at all 4 hH_x_R, reflecting its role as an endogenous ligand.^142^ Notably, the binding affinity profile of histamine (**182**) across hH_x_R is not uniform, with the lowest affinity at hH_1_R in recombinant systems (Table 1). This receptor-dependent pattern is particularly relevant, because histamine (**182**) considered as the endogenous agonist of all H_x_R, whereas its binding affinities clearly differ between receptor subtypes. To further illustrate this, histamine (**182**) is separately depicted across recombinant hH_x_R and nonhuman H_x_R (Fig. 4).

**Fig. 5:**
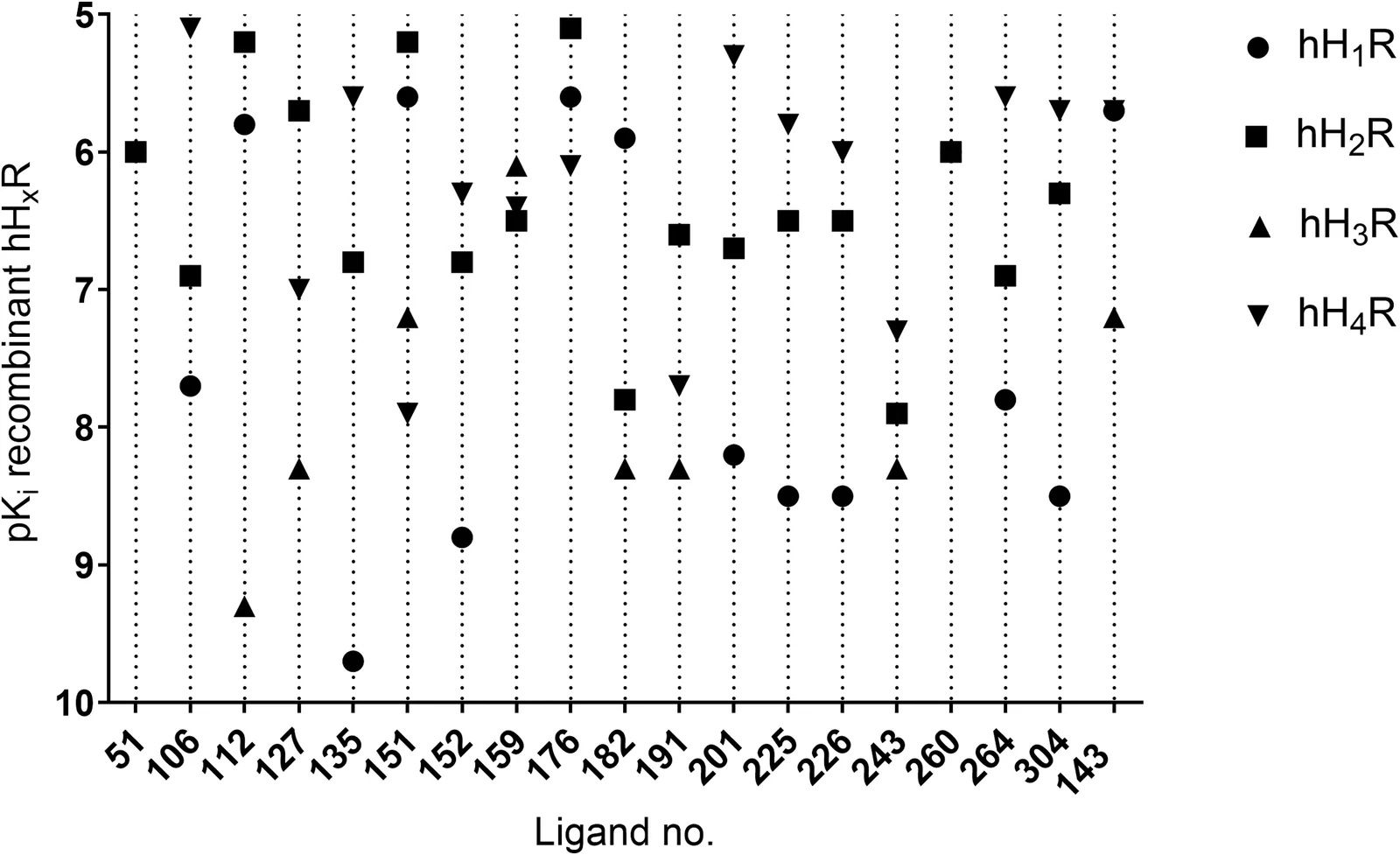
Ligands with a coverage of binding affinity data for multiple hH_x_R. Binding affinities (pK_i_) of selected ligands are shown for hH_1_R–hH_4_R. Each ligand is represented by its assigned ligand number (*x*-axis), and pK_i_ values are plotted on the *y*-axis. Only ligands with experimentally determined binding affinities for at least 3 histamine receptor subtypes are included, allowing comparison of binding profiles on different human histamine receptors. Ligands are sorted from the left to the right in descending order of the highest binding affinity on the hH_1_R: chlorpromazine (**135**), clozapine (**152**), N-desmethylclozapine (**226**), N-desalkylquetiapine (**225**), zotepine (**304**), loxapine (**201**), thioridazine (**264**), 9-OH-risperidone (**106**), SR 147778 (**260**), [5-Fluoro-2-methyl-1-(4-methanesulfonyl-benzenesulfonyl)-1H-indol-3-yl]-acetic acid (**51**), histamine (**182**), ABT-239 (**112**), ciproxifan (**143**), clobenpropit (**151**), GT 2016 (**176**), proxyfan (**243**), impromidine (**191**), dimaprit (**159**), and burimamide (**127**). The according pK_i_ values can be found in Table 1 and Supplemental Table 1.

Conversely, broad antagonism is observed for ciproxifan (**143**).^1^ Ligands with switching behavior across several histamine receptors include impromidine (**191**), arpromidine (**121**), proxyfan (**243**), and clobenpropit (**151**). Figure 5 depicts ligands that were found to have multiple binding affinities across several hH_x_R, visualizing their relative pK_i_ values. Notably, ligands are heterogeneous, ranging from relatively balanced multireceptor affinities, as seen for proxyfan (**243**) and dimaprit (**159**), to pronounced receptor-specific profiles, for example, in chlorpromazine (**135**) or burimamide (**127**). Although broad receptor affinity may have beneficial effects in some medical contexts such as a combination of hH_1_R and hH_2_R antagonism in allergic conditions,^143^ it may also contribute to adverse effects.

**Fig. 6:**
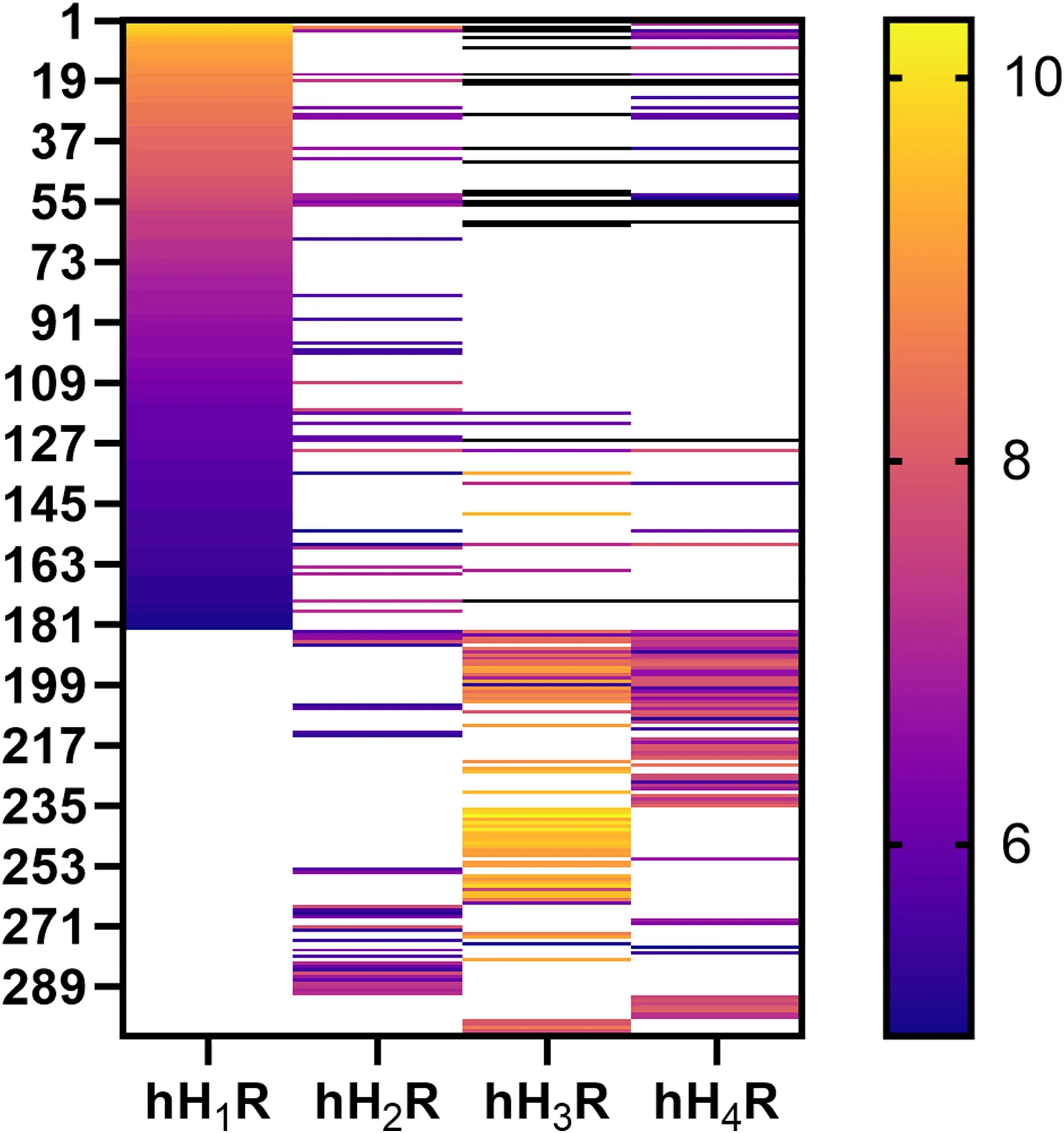
Heat map of binding affinities for all ligands, sorted in descending order of the highest binding affinity on the hH_1_R (followed by the hH_2_R, hH_3_R, and hH_4_R). The according pK_i_ values can be found in Table 1 and Supplemental Table 1, while further heat maps can be found in Supplemental Figs. 2–4. The heat map displays binding affinities (pK_i_) of all ligands identified in the PDSP K_i_ database for hH_1_R–hH_4_R. Each row corresponds to a single ligand, and each column represents one H_x_R. The numerical labels shown on the left side of the heat map indicate the sequential number of ligands displayed and do not correspond to the ligand numbering used in the manuscript or tables (corresponding ligand identifiers can be found in Supplemental Table 12). Binding affinity is visualized by color intensity according to the scale shown on the right, with higher pK_i_ values (stronger binding) indicated by yellow and lower pK_i_ values by blue. White areas show missing binding affinity data, and black areas highlight inactive binding (defined by the binding affinity threshold K_i_ > 10,000 nM). Ligands are vertically ordered based on their highest reported binding affinity at hH_1_R, with ligands showing the strongest hH_1_R binding positioned at the top of the heat map and decreasing affinity toward the bottom. This sorting facilitates direct comparison of ligand binding profiles across receptor subtypes and allows visual identification of ligands with selective, partially overlapping, or broader binding affinity patterns. Differences in color intensity across columns within the same row reflect variations in binding affinity of individual ligands between histamine receptor subtypes. Continuous color gradients indicate receptors with extensive h histamine receptor binding coverage, whereas fragmented or sparse patterns highlight incomplete experimental characterization or selective binding. Overall, the heat map illustrates that many ligands with high hH_1_R affinity also binding actively to other histamine receptor subtypes. At the same time, the presence of rows with strong hH_1_R binding and limited or absent binding at other receptors highlights the occurrence of receptor-selective binding profiles.

Heat maps are presented in Fig. 6 and Supplemental Figs. 2–4, with color intensity reflecting binding strength and ligands ordered by affinity at a given histamine receptor. Overall, hH_1_R and hH_2_R share a moderate degree of similarity, as several ligands with higher affinity at hH_1_R also display measurable binding at hH_2_R, although typically with reduced pK_i_ values. In contrast, hH_3_R shows a more distinct affinity pattern, characterized by clusters of ligands with high hH_3_R selectivity and comparatively low affinity at hH_1_R and hH_2_R. The hH_4_R receptor exhibits the lowest apparent similarity to the other subtypes, with relatively few ligands displaying concurrent high affinity binding across receptors and a generally more heterogeneous and sparse affinity pattern.

## 7 Organ-specific binding affinities for human tissues

Ligands that are listed in the IUPHAR/BPS Guide to Pharmacology with histamine receptors as a target were further examined in the PDSP K_i_ database regarding binding affinities in native human tissues. These compounds and their organ-specific binding affinities are summarized in Supplemental Table 9.

For 37 ligands, binding data for different tissues are listed. Notably, all reported tissues are of cerebral origin, including several anatomical regions of the brain. These include the frontal and cerebral cortex, cortical membranes, as well as brain and cortex in general. The hH_x_R examined in native tissues are restricted to hH_1_R and hH_3_R. Reported pK_i_ values span from <5 to 10.1.

Several tissues were examined for the 11 ligands amitriptyline (**116**), chlorpromazine (**135**), clozapine (**152**), diphenhydramine (**162**), doxepin (**164**), haloperidol (**180**), histamine (**182**), loxapine (**201**), molindone (**216**), perphenazine (**237**), and thioperamide (**263**). However, for most ligands, binding data are available for only a single native human tissue. With regard to receptor subtypes, histamine (**182**) is the only ligand examined for both hH_1_R and hH_3_R.

**Fig. 7:**
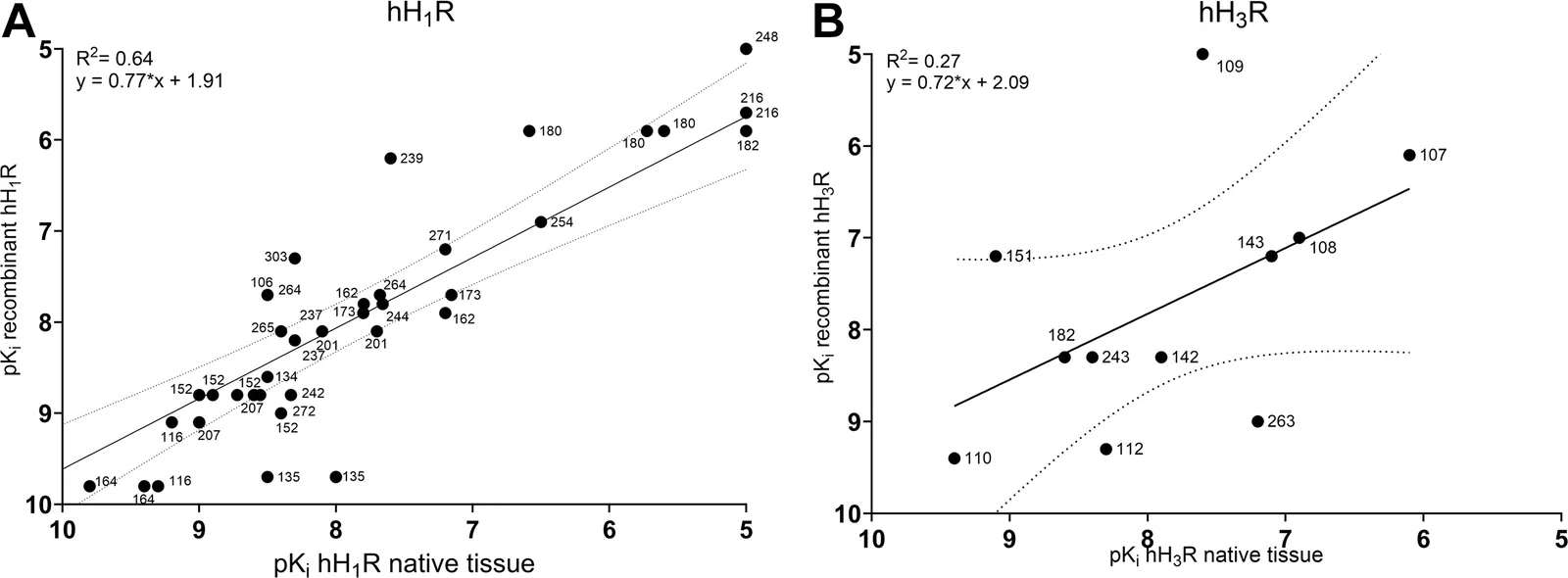
Binding affinities (pK_i_) of human native tissues (*x*-axis) in comparison with recombinant hH_x_R (*y*-axis), a linear regression line, and the 95% CI. Results were available for (A) the hH_1_R and (B) the hH_3_R. For each linear correlation, the coefficient of determination (*R*^2^) and the mathematical equation is provided. Ligands are labeled by their assigned number. The according pK_i_ values can be found in Supplemental Table 9.

For most ligands, binding affinities for both recombinant hH_x_R as well as native human tissues were available. Comparisons between these data sets reveal deviations in pK_i_ values ranging from 0.0 to 4.16. In addition to absolute differences, linear correlations between binding affinities obtained from native versus recombinant hH_x_R were visualized (Fig. 7).

On the hH_1_R receptor, pK_i_ values derived from native tissues show a clear positive linear relationship with those obtained from recombinant hH_x_R, for example, perphenazine, (**237**), loxapine (**201**), amitriptyline (**116**), or chlorpheniramine (**134**), indicating a good overall agreement between experimental models. However, there are also ligands that are displayed outside the 95% CI of the linear regression line, for example, olanzapine (**232**), chlorpromazine (**135**), or pimozide (**239**), and have a considerable deviation between pK_i_ values (Supplemental Table 9).

hH_3_R displays a weaker and more variable correlation, with a broader scatter of data points and reduced correspondence, for example, in thioperamide (**263**), ABT-239 (**112**), and A-331440 (**109**), being displayed outside the 95% confidence interval (CI) of the linear regression line. However, also for hH_3_R, some ligands align very well without a considerable deviation between systems, such as A-304121(**107**), A-317920 (**108**), or A-349821 (**110**).

## 8 Species-specific binding affinities

Analogous to the analysis of human tissues, binding affinities across different species were summarized. Here, all initially listed compounds from the PDSP K_i_ database were analyzed. All derived species-specific binding affinities are presented in Supplemental Table 10 and Supplemental Fig. 5.

For 93 ligands, binding data for different species are available, including dog (d), guinea pig (gp), mouse (m), and rabbit (r). Reported tissues include brain, brain cortex and cerebral cortex, cerebellum, cortical membranes, lung, myenteric plexus, as well as cloned receptors and Sf9 expression systems. Data are available for all H_x_R, with a high proportion of H_1_R-related measurements and comparatively few entries for H_4_R. Reported pK_i_ values span from <5 to 10.1 (Supplemental Fig. 5).

Several species were examined for 28 ligands. A subset of ligands was listed particularly frequently, including histamine (**182**, *n* = 10), triprolidine (**272**, *n* = 5), mepyramine (**207**, *n* = 5), and thioperamide (**263**, *n* = 5). Furthermore, for many ligands, information is available for several tissues and H_x_R. Triprolidine (**272**) was examined in several species (gp, r, and m) and tissues (brain, cerebellum, and cloned), although binding data were restricted to the H_1_R. Similarly, thioperamide (**263**) was investigated across several species (gp and d), tissues (lung, brain cortex, and cloned), and H_x_R subtypes (H_1_R–H_3_R). The most comprehensive data are available for histamine (**182**) in the guinea pig, covering multiple tissues (cerebellum, cerebral cortex, cortical membranes, myenteric plexus, cloned, and Sf9) and all H_x_R (gpH_1_R–gpH_4_R). In guinea pig systems, histamine (**182**) binding affinities vary markedly between gpH_x_R subtypes and tissues (Fig. 4). Particularly, gpH_1_R values differ between brain, cerebellum and recombinant H_x_R, whereas gpH_4_R values show tissue-dependent variability. These findings illustrate that even for the endogenous ligand, H_x_R binding affinities are strongly receptor- and system-dependent.

**Fig. 8:**
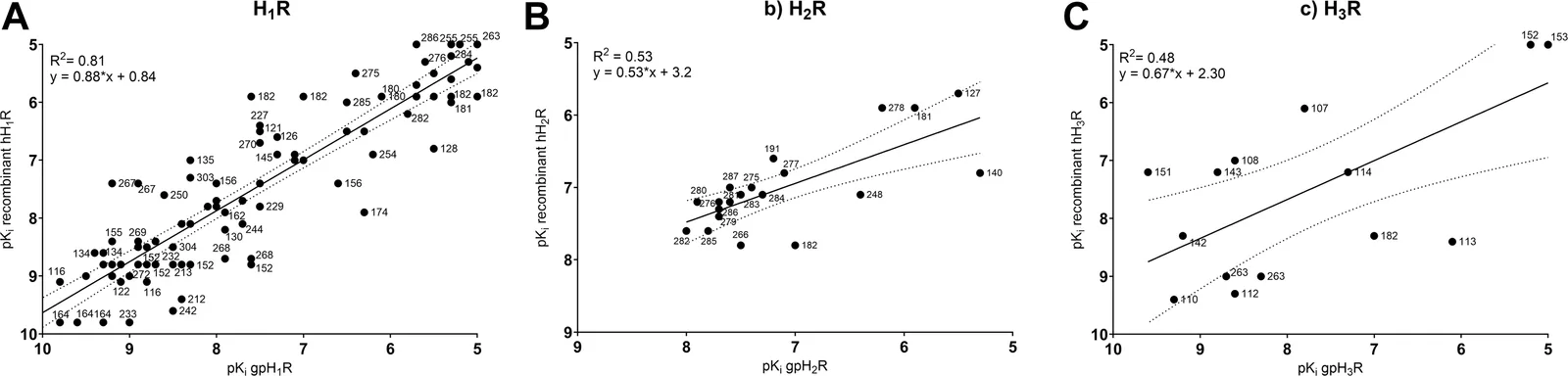
Binding affinities (pK_i_) of the nonhuman species guinea pig (gp) (*x*-axis) in comparison with recombinant hH_x_R (*y*-axis), a linear regression line, and the 95% CI. It was decided to examine the linear correlation for hH_x_R vs gpHxR, since this is the species where most data are available. Results are presented for all receptor subtypes: (A) H_1_R, (B) H_2_R, and (C) H_3_R. The H_4_R was excluded from the correlation analysis because of the very limited number of available data points. In general, correlation analyses were restricted to receptor subtypes with sufficient data density, as statistical evaluation based on very small numbers of data points would not be meaningful. For each linear correlation, the coefficient of determination (*R*^2^) and the mathematical equation is provided. Ligands can be identified by their assigned numbers. The H_2_R and H_3_R are fully labeled, whereas only outliers (95% CI) are labeled for the H_1_R because of the large number of data points. The according pK_i_ values can be found in Supplemental Table 10.

For most examined ligands, binding affinities for both recombinant hH_x_R, as well as for nonhuman species were available (Supplemental Table 10). Figure 8 illustrates the comparison between binding affinities measured in guinea pig versus recombinant hH_x_R across H_1_R-H_3_R. For H_1_R, many data points and a strong linear correlation are observed, indicating a high degree of concordance between guinea pig and recombinant hH_x_R. This is particularly pronounced for astemizole (**122**), escitalopram (**167**), fluphenazine (**173**), or zotepine (**304**). In contrast, several outliers are observable, for example, trans-H2-PAT (−) (**267**), fluspirilene (**174**), or mianserin (**212**), being displayed outside the 95% CI.

In contrast, H_2_R and H_3_R displays a moderate number of data points and a moderate linear correlation. Although for the H_2_R, some ligands are roughly concordant between species, such as UR-AK22 (**275**), UR-AK46 (**279**), UR-AK49 (**280**), or UR-AK57 (**282**), others are strongly deviating and depicted outside the 95% CI, for example, cimetidine (**140**) or UR-AK41 (**278**). A similar situation is observed for the H_3_R, although with a more scattered distribution and a lower number of data points. For example, the ligands S-*α*-methylhistamine (**114**) and cipralisant (**142**) have comparable binding affinities, whereas strong deviations are observed for A-304121 (**107**), R-*α*-methylhistamine (**113**), or clobenpropit (**151**). Only very few binding affinity values are available for gpH_4_R, therefore, no correlation analysis was performed for this receptor subtype.

Overall, these comparisons demonstrate that the degree of concordance between guinea pig and recombinant hH_x_R varies between subtypes, with H_1_R showing the highest similarity. These species-dependent differences likely reflect both biological and methodological aspects. Besides differences in receptor expression systems and tissues, variations in the primary amino acid sequence of H_x_R between species^4^ may influence ligand binding behavior^4^ and ligand association and dissociation characteristics,^23^ resulting in differences in measured pK_i_ values. Supplemental Table 11 provides an overview of key literature about species-dependent differences.

## 9 Variation of binding affinities in human native tissues and different species versus recombinant hH_x_R

When comparing recombinant hH_x_R with native human tissues and across different species, there are strong variations in binding affinities for the same ligand (Figs. 4, 7, and 8). The deviation for brain tissues ranges from 0.0 for perphenazine (**237**) (hH_1_R, frontal cortex) up to 4.16 for olanzapine (**232**) (hH_1_R, brain) (Fig. 5; Supplemental Table 9). Similarly, there are large deviations between recombinant hH_x_R and other species, with ranges of binding affinities (pK_i_) from 0.0 for astemizole (**122**) and escitalopram (**167**) at the gpH_1_R, up to 3.2 for R-*α*-methylhistamine (**246**) at the gpH_4_R (Fig. 8; Supplemental Table 10).

The relationship between native tissues versus recombinant systems differs between hH_x_R (Fig. 7). For hH_1_R, binding affinities obtained from human native tissues show a positive linear correlation with values derived from recombinant hH_x_R, indicating that recombinant expression systems provide a reliable approximation of native hH_1_R binding properties. In contrast, the corresponding comparison for hH_3_R reveals a substantially weaker and more scattered relationship, with limited concordance between native and recombinant binding affinities. This pronounced difference between hH_1_R and hH_3_R likely reflects receptor-specific biological and experimental factors. The hH_1_R is broadly and robustly expressed in human brain tissues and exhibits comparatively stable pharmacological properties across experimental conditions, resulting in a strong agreement between native and recombinant measurements. In contrast, hH_3_R binding appears to be more sensitive to tissue viability and experimental variability. This sensitivity may be exacerbated in postmortem brain samples, where variations in tissue quality can disproportionately affect hH_3_R binding, whereas hH_1_R binding remains more stable. Consequently, recombinant systems are less predictive of native hH_3_R binding than for hH_1_R. Furthermore, numerous splice variants of hH_3_R have been described, which may differ in regional tissue expression and pharmacological properties.^144^ This implies that native tissue preparations may contain heterogeneous receptor populations with variable ligand binding behavior, potentially contributing to the broader variability in hH_3_R pK_i_ values compared with hH_1_R.^106^

Analogous to recombinant hH_x_R, there are also deviations in duplicates within similar native tissues and species. The pK_i_ deviation ranges in human brain tissues from 0.0–3.95, and across species from 0.02–1.04 (Supplemental Tables 9 and 10).

Such strong variations between cloned versus native tissues, as well as between species, have already been observed and reported earlier, likely reflecting a combination of biological and experimental factors (as discussed in section Variation of Recombinant hHxR Binding Affinities: Deviation in Replicate Values and Comparison With the IUPHAR/BPS Guide to Pharmacology). This may explain why certain ligands markedly deviate from the regression line and 95% CI of species- and tissue-comparisons (Figs. 7 and 8). The histamine-specific (**182**) comparison further supports this interpretation (Fig. 4). Although histamine (**182**) activates all H_x_R, its affinity differs substantially between receptor subtypes and experimental systems. Thus, system-dependent variation is also observed for the endogenous ligand itself. This highlights that receptor subtype, species and tissues are important aspects to consider in experimental studies and data interpretation and restrict the comparison of results across experimental systems.^1^^,^^4^^,^^92^^,^^145^

## 10 Current challenges and knowledge gaps

The analysis of ligands and binding affinities is strongly dependent on the PDSP K_i_ database, the experimental data from the summarized primary literature, and the IUPHAR/BPS Guide to Pharmacology. Data restrictions may affect the completeness of the overview. This applies to all information, including binding affinities of H_x_R, native tissues, species, and functional labels. The observed deviations in pK_i_ values result in limited precision, whereas there is also the risk of outdated functional labels, particularly regarding the distinction between antagonists and inverse agonists.

Another important restriction is the initial selection of ligands with binding affinities below 10,000 nM. Although this inclusion criterion is widely applied and necessary to filter relevant data, it may limit the completeness of the characterization of ligands. In combination with incomplete receptor profiling, this threshold further restricts the ability to draw definitive conclusions regarding H_x_R selectivity for many ligands. It is not always possible to clearly identify highly selective behavior if the ligand has not been examined on all hH_x_R.

Despite the large amount of available data, significant information is still missing. Many ligands have not been examined on all 4 H_x_R, and with respect to their functional behavior (agonist, antagonist, and inverse agonist) (Table 2). Furthermore, even reliable and widely used databases remain incomplete. The IUPHAR/BPS Guide to Pharmacology frequently omits hH_x_R targets for compounds that are otherwise available through ligand searches (Supplemental Table 5). Similarly, the PDSP K_i_ database does not provide binding affinity values for all ligands and receptor subtypes in recombinant hH_x_R, likely because the corresponding experimental analyses have not been conducted. The present literature comparison further indicates that a considerable proportion of experimentally determined binding affinity data is not captured both or either database (Supplemental Table 7). Thus, the observed data gaps are likely attributable not only to missing experimental studies, but also to the selective curation strategies and incomplete integration of existing literature into these resources. Importantly, these findings indicate that apparent “missing data” may partly reflect differences in database design and inclusion criteria rather than a true absence of experimental evidence.

## 11 Conclusions

The analysis of H_x_R ligands reveals many compounds with binding affinity on hH_x_R (Table 1; Supplemental Tables 1 and 2). These ligands can be broadly categorized as being either selective or exhibiting broader activity across H_x_R (Fig. 4, Fig. 5, Fig. 6; Supplemental Figs. 2–4). Comparisons of binding affinities across hH_1_R–hH_4_R show that, despite differences in receptor specificity, there are substantial similarities, being reflected by both shared ligands and comparable ranges and distributions of binding affinities across H_x_R. Notably, hH_1_R and hH_2_R exhibit significant overlap in recombinant hH_x_R, and several ligands demonstrate concurrent binding to hH_3_R and, to a lesser extent, hH_4_R (Fig. 6; Supplemental Fig. 2–4). Overall, hH_x_R ligands are highly versatile, and several of the listed compounds are already approved as therapeutic drugs.

Comparisons across different native tissues and species confirm that substantial differences in binding affinities exist (Supplemental Tables 9 and 10; Figs. 4, 7, and 8). This makes it essential to refer consistently to a specific experimental system when investigating and comparing ligand binding. However, even within similar experimental conditions, considerable variation can occur between duplicates and repeated experiments (Table 1; Supplemental Tables 1, 5, and 10). This highlights the need for critical assessment of experimental methodology and data reliability.

The main contribution of this work lies in the systematic integration and comparison of PDSP K_i_ and IUPHAR/BPS data sources. Using H_x_R as a model, this study demonstrates how database-based analyses can uncover similarities, differences, and knowledge gaps in ligand pharmacology in a structured manner. Importantly, the comparison highlights that the PDSP K_i_ database and the IUPHAR/BPS Guide to Pharmacology are not directly equivalent resources, but rather reflect different strategies of data aggregation and curation. The PDSP K_i_ database provides broad coverage of ligand binding affinities, including many compounds with limited receptor profiling, whereas the IUPHAR/BPS Guide to Pharmacology focuses on a curated subset of representative ligands supported by selected references. Consequently, differences between the databases should not be interpreted solely as missing data, but also as a result of selection principles and differing scope.

The inclusion of ligands with targets beyond hH_x_R reflects the integrative approach of the study, which is based on reported binding affinities rather than therapeutic classification. This allows the identification of off-target interactions and broader pharmacological profiles that may not be captured when focusing solely on clinically defined histamine receptor ligands. Importantly, these findings highlight that high affinity at hH_x_R does not necessarily correspond to pharmacological selectivity, but may reflect secondary or off-target interactions.

Overall, this analysis indicates that improving the integration of existing experimental data across databases may be as important as generating new data. A more systematic incorporation of published binding affinity studies into pharmacological databases would enhance data completeness, comparability, and utility for drug development and receptor pharmacology.

## 12 Perspective on future directions

Future research is necessary for a more advanced characterization of hH_x_R ligands and their binding affinities. Many compounds still require classification regarding their functional profile, and binding affinity values remain incomplete for several receptors, particularly hH_4_R, for which binding affinity data and functional characterization remain comparatively sparse. In addition, comparisons between recombinant hH_x_R, native tissues, and different species are of high value. The PDSP K_i_ database offers potential for systematic receptor analyses, including comparative ligand profiling, identification of selectivity patterns, and identification of compounds for experimental and computational follow-up studies. Although this work focuses on H_x_R, comparable state-of-the-art overviews are currently lacking for most GPCR families, underscoring a broader need for comprehensive ligand profiling. The approach presented here is not limited to hH_x_R and can be applied to other GPCR families to support ligand characterization and target evaluation.

## Conflict of interest

The authors declare no conflicts of interest.
