# Supplementary material for: Binding affinities for histamine receptors 1 to 4: Systematic comparison of ligands from the Psychoactive Drug Screening Program Ki database and International Union of Basic and Clinical Pharmacology/British Pharmacological Society Guide to Pharmacology
Source: Mol Pharmacol. 2026 Jun 12;108(7):100139. doi: 10.1016/j.molpha.2026.100139 (PMC13494103; doi:10.1016/j.molpha.2026.100139)
Supplement: Supplementary file 1 [file mmc1.docx]

**Binding affinities for histamine receptors 1-4: systematic comparison of ligands from the PDSP K_i_ database and IUPHAR/BPS Guide to Pharmacology – Supplement**

**Lilly Josephine Bindel and Roland Seifert**

Supplementary Figures

***Supplemental Figure 1:*** *Distribution (%) of ligands per binding affinity (pK_i_) for hH_x_R, retrieved from the PDSP K_i_ database. Data was available for all hH_x_R: a) H_1_R, b) H_2_R, c) H_3_R, d) H_4_R. The visualized pK_i_ values can be found in Table 1 and Supplemental Table 1.*

***Supplemental Figure 2:*** *Heat map of binding affinities for all ligands, sorted in descending order of the highest binding affinity on the hH_2_R (followed by the hH_1_R, hH_3_R and hH_4_R). The according pK_i_ values can be found in Table 1 and Supplemental Table 1, while further heat maps can be found in Figure 5 and Supplemental Figure 3-4.*

*The heat map displays binding affinities (pK_i_ values) of all ligands identified in the PDSP K_i_ database for hH_1_R–hH_4_R. Each row corresponds to a single ligand, and each column represents one H_x_R. The numerical labels shown on the left side of the heat map indicate the sequential number of ligands displayed and do not correspond to the ligand numbering used in the manuscript or tables (corresponding ligand identifiers can be found in Supplemental Table 12). Binding affinity is visualized by colour intensity according to the scale shown on the right, with higher pK_i_ values (stronger binding) indicated by yellow and lower pK_i_ values by blue. Wite areas show missing binding affinity data, and black areas highlight inactive binding (defined by the binding affinity threshold K_i_ > 10,000 nM). Ligands are vertically ordered based on their highest reported binding affinity at h*H_2_*R, with ligands showing the strongest h*H_2_*R binding positioned at the top of the heat map and decreasing affinity towards the bottom. This sorting facilitates direct comparison of ligand binding profiles across receptor subtypes and allows visual identification of ligands with selective, partially overlapping, or broader binding affinity patterns. Differences in colour intensity across columns within the same row reflect variations in binding affinity of individual ligands between histamine receptor subtypes. Continuous colour gradients indicate receptors with extensive h histamine receptor binding coverage, whereas fragmented or sparse patterns highlight incomplete experimental characterisation or selective binding. Overall, the heat map illustrates that a subset of ligands with high h*H_2_*R affinity also displays binding activity at other histamine receptor subtypes, while many ligands show limited or absent binding outside h*H_2_*R, indicating pronounced receptor selectivity.*

***Supplemental Figure 3:*** *Heat map of binding affinities for all ligands, sorted in descending order of the highest binding affinity on the hH_3_R (followed by the hH_1_R, hH_2_R and hH_4_R). The according pK_i_ values can be found in Table 1 and Supplemental Table 1, while further heat maps can be found in Figure 5, Supplemental Figure 2 and 4.*

*The heat map displays binding affinities (pK_i_ values) of all ligands identified in the PDSP K_i_ database for hH_1_R–hH_4_R. Each row corresponds to a single ligand, and each column represents one H_x_R. The numerical labels shown on the left side of the heat map indicate the sequential number of ligands displayed and do not correspond to the ligand numbering used in the manuscript or tables (corresponding ligand identifiers can be found in Supplemental Table 12). Binding affinity is visualized by colour intensity according to the scale shown on the right, with higher pK_i_ values (stronger binding) indicated by yellow and lower pK_i_ values by blue. Wite areas show missing binding affinity data, and black areas highlight inactive binding (defined by the binding affinity threshold K_i_ > 10,000 nM). Ligands are vertically ordered based on their highest reported binding affinity at hH_3_R, with ligands showing the strongest hH_3_R binding positioned at the top of the heat map and decreasing affinity towards the bottom. This sorting facilitates direct comparison of ligand binding profiles across receptor subtypes and allows visual identification of ligands with selective, partially overlapping, or broader binding affinity patterns. Differences in colour intensity across columns within the same row reflect variations in binding affinity of individual ligands between histamine receptor subtypes. Continuous colour gradients indicate receptors with extensive h histamine receptor binding coverage, whereas fragmented or sparse patterns highlight incomplete experimental characterisation or selective binding. Overall, the heat map illustrates that ligands with high hH_3_R affinity frequently show limited binding to hH_1_R and h*H_2_*R, while a subset displays concurrent binding activity at hH_4_R, reflecting both receptor selectivity and broader binding characteristics.*

***Supplemental Figure 4:*** *Heat map of binding affinities for all ligands, sorted in descending order of the highest binding affinity on the hH_4_R (followed by the hH_1_R, hH_2_R and hH_3_R). The according pK_i_ values can be found in Table 1 and Supplemental Table 1, while further heat maps can be found in Figure 5, Supplemental Figure 2 and 3.*

*The heat map displays binding affinities (pK_i_ values) of all ligands identified in the PDSP K_i_ database for hH_1_R–hH_4_R. Each row corresponds to a single ligand, and each column represents one H_x_R. The numerical labels shown on the left side of the heat map indicate the sequential number of ligands displayed and do not correspond to the ligand numbering used in the manuscript or tables (corresponding ligand identifiers can be found in Supplemental Table 12). Binding affinity is visualized by colour intensity according to the scale shown on the right, with higher pK_i_ values (stronger binding) indicated by yellow and lower pK_i_ values by blue. Wite areas show missing binding affinity data, and black areas highlight inactive binding (defined by the binding affinity threshold K_i_ > 10,000 nM). Ligands are vertically ordered based on their highest reported binding affinity at hH_4_R, with ligands showing the strongest hH_4_R binding positioned at the top of the heat map and decreasing affinity towards the bottom. This sorting facilitates direct comparison of ligand binding profiles across receptor subtypes and allows visual identification of ligands with selective, partially overlapping, or broader binding affinity patterns. Differences in colour intensity across columns within the same row reflect variations in binding affinity of individual ligands between histamine receptor subtypes. Continuous colour gradients indicate receptors with extensive h histamine receptor binding coverage, whereas fragmented or sparse patterns highlight incomplete experimental characterisation or selective binding. Overall, the heat map illustrates that ligands with high hH_4_R affinity frequently show limited binding to hH_1_R, h*H_2_*R, and hH_3_R, underscoring the comparatively high degree of receptor selectivity observed for hH_4_R ligands.*

***Supplemental Figure 5:*** *Overview about species-specific binding affinities on H_x_R for non-human species. The y-axis depicts the binding affinity (pK_i_), while on the x-axis available species are listed (in comparison with recombinant hH_x_R). Depicted is the median, as well as minimum and maximum. Data was available for all histamine receptors: a) H_1_R, b) H_2_R, c) H_3_R, d) H_4_R. The according pK_i_ values can be found in Supplemental Table 10.*

Supplementary Tables

***Supplemental Table 1:*** *Overview about binding affinities (p*K_i_*) and ligands for the hH_1_R- hH_4_R. If duplicate values are available, the median is given and the range provided. The label “inactive” is assigned to ligands where available data report a binding affinity (K_i_) above 10,000 nM, while the label “no data” highlights that no binding affinity is available in the PDSP K_i_ database. Ligands are sorted in descending order from the highest binding affinity for the hH_1_R. A visualization can be found in Figure 2 and 3.*

| **Ligand name** | **Ligand no.** | **hH_1_R** | | **hH_2_R** | | **hH_3_R** | | **hH_4_R** | |
| --- | --- | --- | --- | --- | --- | --- | --- | --- | --- |
|  |  | **pK_i_ (single listing or median)** | **pK_i_ range** | **pK_i_ (single listing or median)** | **pK_i_ range** | **pK_i_ (single listing or median)** | **pK_i_ range** | **pK_i_ (single listing or median)** | **pK_i_ range** |
| Cyproheptadine | **154** | 10.2 |  | no data |  | no data |  | 6.7 |  |
| ORG-5222 | **233** | 9.8 |  | 8.6 |  | no data |  | no data |  |
| Doxepin | **164** | 9.8 | 0.6 | no data |  | inactive |  | 7.0 |  |
| Chlorpromazine | **135** | 9.7 | 1.5 | 6.8 |  | inactive |  | 5.6 | 2.0 |
| Promethazine | **242** | 9.6 |  | no data |  | no data |  | 6.8 |  |
| Mianserin | **212** | 9.4 | 0.8 | no data |  | inactive |  | 6.1 |  |
| (2RS,3aRS,12bSR)-2-N,N-Dimethylaminomethyl-11-fluoro-2,3,3a,12b-tetrahydrodibenzo[b,f]furo[2,3-d]tiepin | **21** | 9.3 |  | no data |  | no data |  | no data |  |
| (-)-(2RS,3aRS,12bSR)-2-N,N-Dimethylaminomethyl-11-fluoro-2,3,3a,12b-tetrahydrodibenzo[b,f]furo[2,3-d]tiepin | **1** | 9.2 |  | no data |  | no data |  | no data |  |
| Amitriptylin | **116** | 9.1 | 0.3 | no data |  | inactive |  | 7.5 |  |
| Mizolastine | **215** | 9.1 |  | no data |  | no data |  | no data |  |
| Astemizole | **122** | 9.1 |  | no data |  | no data |  | no data |  |
| Flupenthixol, α | **171** | 9.1 |  | no data |  | no data |  | no data |  |
| Hydroxyzine, (R) | **184** | 9.0 |  | no data |  | no data |  | no data |  |
| Triprolidine | **272** | 9.0 | 0.6 | no data |  | no data |  | no data |  |
| (2SR,3aRS,12bSR)-2-N,N-Dimethylaminomethyl-11-fluoro-2,3,3a,12b-tetrahydrodibenzo[b,f]furo[2,3-d]tiepin | **24** | 8.9 |  | no data |  | no data |  | no data |  |
| (+)-(2RS,3aRS,12bSR)-2-N,N-Dimethylaminomethyl-11-fluoro-2,3,3a,12b-tetrahydrodibenzo[b,f]furo[2,3-d]tiepin | **6** | 8.9 |  | no data |  | no data |  | no data |  |
| Clozapine | **152** | 8.8 | 2.9 | 6.8 |  | inactive |  | 6.3 | 0.6 |
| Mepyramine | **207** | 8.8 | 0.7 | no data |  | no data |  | no data |  |
| Olanzapine | **232** | 8.8 | 0.9 | 7.4 |  | inactive |  | inactive |  |
| Mirtazapine | **213** | 8.8 | 0.5 | no data |  | inactive |  | inactive |  |
| Ketanserin | **196** | 8.7 |  | no data |  | no data |  | no data |  |
| 8-Chloro-11-(4-ethylpiperazin-1-yl)-5H-dibenzo[b,e][1,4]diazepine | **102** | 8.7 |  | no data |  | no data |  | no data |  |
| trans-H_2_-PAT(-) | **267** | 8.7 | 0.2 | no data |  | no data |  | no data |  |
| Chlorpheniramine | **134** | 8.6 | 0.0 | no data |  | no data |  | 5.5 |  |
| Terfenadine | **261** | 8.6 | 0.2 | no data |  | no data |  | no data |  |
| Levocetirizine | **198** | 8.5 |  | no data |  | no data |  | no data |  |
| Zotepine | **304** | 8.5 | 1.0 | 6.3 |  | no data |  | 5.7 |  |
| trans-H_2_-PAT(+/-) | **269** | 8.5 | 0.2 | no data |  | no data |  | no data |  |
| N-desmethylclozapine | **226** | 8.5 |  | 6.5 |  | inactive |  | 6.0 |  |
| N-Desalkylquetiapine | **225** | 8.5 |  | 6.5 |  | no data |  | 5.8 |  |
| d-Chlorpheniramine | **155** | 8.4 | 0.3 | no data |  | no data |  | no data |  |
| (2RS,3aRS,12bSR)-2-N,N-Dimethylaminomethyl-11-chloro-2,3,3a,12b-tetrahydrodibenzo[b,f]furo[2,3-d]oxepin | **19** | 8.4 |  | no data |  | no data |  | no data |  |
| 8-Chloro-11-(4-isopropylpiperazin-1-yl)-5H-dibenzo[b,e][1,4]diazepine | **103** | 8.3 |  | no data |  | no data |  | no data |  |
| 8-Chloro-11-[4-(1,1-dideutrioethyl)piperazin-1-yl]-5H-dibenzo[b,e][1,4]diazepine | **104** | 8.3 |  | no data |  | no data |  | no data |  |
| ucb 29992, (R) | **273** | 8.3 |  | no data |  | no data |  | no data |  |
| Flupenthixol, β | **172** | 8.2 |  | no data |  | no data |  | no data |  |
| 11-(Piperazin-1-yl)-5H-dibenzo[b,e][1,4]diazepine | **69** | 8.2 |  | no data |  | no data |  | no data |  |
| Cetirizine | **130** | 8.2 | 0.2 | no data |  | no data |  | no data |  |
| Loxapine | **201** | 8.2 | 3.2 | 6.7 |  | inactive |  | 5.3 |  |
| (2RS,3aRS,12bSR)-2-N,N-Dimethylaminomethyl-11-fluoro-2,3,3a,12b-tetrahydrodibenzo[b,f]furo[2,3-d]oxepin | **20** | 8.1 |  | no data |  | no data |  | no data |  |
| cis-H_2_-PAT(-) | **144** | 8.1 | 0.1 | no data |  | no data |  | no data |  |
| Thiothixene | **265** | 8.1 | 0.5 | 6.4 |  | no data |  | no data |  |
| Perphenazine | **237** | 8.1 |  | no data |  | inactive |  | inactive |  |
| Quetiapine | **244** | 8.1 | 0.7 | no data |  | no data |  | no data |  |
| GBR-12909 | **175** | 8.0 |  | no data |  | no data |  | no data |  |
| Ebastine | **166** | 8.0 |  | no data |  | no data |  | no data |  |
| Fexofenadine | **170** | 8.0 | 0.0 | no data |  | no data |  | no data |  |
| Diphenhydramine | **162** | 7.9 | 0.1 | no data |  | no data |  | no data |  |
| Fluspirilene | **174** | 7.9 |  | no data |  | no data |  | no data |  |
| (2SR,3aRS,12bSR)-2-N,N-Dimethylaminomethyl-11-chloro-2,3,3a,12b-tetrahydrodibenzo[b,f]furo[2,3-d]oxepin | **22** | 7.9 |  | no data |  | no data |  | no data |  |
| cis-H_2_-PAT(+/-) | **146** | 7.8 | 0.0 | no data |  | no data |  | no data |  |
| Nortryptyline | **229** | 7.8 |  | no data |  | inactive |  | no data |  |
| Thioridazine | **264** | 7.8 | 0.1 | 6.9 |  | inactive |  | 5.6 |  |
| 9-OH-risperidone | **106** | 7.7 | 0.8 | 6.9 |  | no data |  | 5.1 |  |
| Fluphenazine | **173** | 7.7 | 1.0 | 6.3 |  | inactive |  | inactive |  |
| Risperidone | **250** | 7.6 | 0.7 | 6.9 |  | inactive |  | inactive |  |
| Cariprazine | **129** | 7.6 |  | no data |  | no data |  | no data |  |
| Aripiprazole | **120** | 7.5 | 0.1 | no data |  | no data |  | no data |  |
| (2SR,3aRS,12bSR)-2-N,N-Dimethylaminomethyl-11-fluoro-2,3,3a,12b-tetrahydrodibenzo[b,f]furo[2,3-d]oxepin | **23** | 7.5 |  | no data |  | no data |  | no data |  |
| trans-H_2_-PAT(+) | **268** | 7.4 | 0.2 | no data |  | no data |  | no data |  |
| Imipramine | **187** | 7.4 |  | no data |  | inactive |  | inactive |  |
| Desipramine | **156** | 7.4 | 0.2 | no data |  | inactive |  | no data |  |
| Cetirizine (+) isomer | **131** | 7.4 | 1.4 | no data |  | no data |  | no data |  |
| Cimbi-775 (+) | **12** | 7.4 |  | no data |  | no data |  | no data |  |
| (2RS,3aRS,12bSR)-2-N,N-Dimethylaminomethyl-11-bromo-2,3,3a,12b-tetrahydrodibenzo[b,f]furo[2,3-d]oxepin | **18** | 7.4 |  | no data |  | no data |  | no data |  |
| Ziprasidone | **303** | 7.3 | 0.9 | 5.5 |  | no data |  | no data |  |
| Cimbi-772 (-) | **2** | 7.2 |  | no data |  | no data |  | no data |  |
| Triflouperazine | **271** | 7.2 |  | no data |  | no data |  | no data |  |
| ucb 29993, (S) | **274** | 7.2 |  | no data |  | no data |  | no data |  |
| MDL 100907 | **204** | 7.2 | 0.0 | no data |  | no data |  | no data |  |
| pdspTestCompound3 | **236** | 7.2 |  | no data |  | no data |  | no data |  |
| R-Chlopheniramine | **50** | 7.1 |  | no data |  | no data |  | no data |  |
| MRS5979 | **218** | 7.1 |  | no data |  | no data |  | no data |  |
| Terfenadine, R(+) | **262** | 7.0 |  | no data |  | no data |  | no data |  |
| SCH 23390 | **253** | 7.0 |  | no data |  | no data |  | no data |  |
| l-Chlorpheniramine | **197** | 7.0 | 3.1 | no data |  | no data |  | no data |  |
| (+)-7,8-Dimethoxy-3-[4-(2-methyl-3-phenyl-2(E)-propen-1-yl)piperazin-1-ylmethyl]-3a,4-dihydro-3H-[1]benzopyrano[4,3-c]isoxazole | **7** | 6.9 |  | no data |  | no data |  | no data |  |
| cis-H_2_-PAT(+) | **145** | 6.9 | 0.0 | no data |  | no data |  | no data |  |
| Br-6-APT | **125** | 6.9 |  | no data |  | no data |  | no data |  |
| Sertindole | **254** | 6.9 | 0.0 | no data |  | no data |  | no data |  |
| (+)-Cimbi-772 | **11** | 6.9 |  | no data |  | no data |  | no data |  |
| Butaclamol,(+) | **128** | 6.8 |  | no data |  | no data |  | no data |  |
| FECIMBI-36 | **169** | 6.8 |  | 5.7 |  | no data |  | no data |  |
| 2-{4-[4-(7-Methoxy-naphthalen-1-yl)piperazin-1-yl]-butyl}-4-methyl-2H-[1,2,4]triazine-3,5-dione | **78** | 6.8 |  | no data |  | no data |  | no data |  |
| Loratidine | **200** | 6.8 |  | no data |  | no data |  | no data |  |
| 4-(8-Chloro-5H-dibenzo[b,e][1,4]diazepin-11-yl)-1-ethylpiperazine N-oxide | **90** | 6.8 |  | no data |  | no data |  | no data |  |
| Cimbi-717 | **137** | 6.8 |  | no data |  | no data |  | no data |  |
| 2-(Dimethylamino)tetralin | **74** | 6.8 |  | no data |  | no data |  | no data |  |
| (+/-)-BMY-1402 | **14** | 6.7 |  | no data |  | no data |  | no data |  |
| Trazodone | **270** | 6.7 |  | 5.5 |  | no data |  | no data |  |
| N-(9,10-Dihydro-9-anthracenylmethyl)-2-phenylethanamine | **219** | 6.6 |  | no data |  | no data |  | no data |  |
| BU-E 47 | **126** | 6.6 |  | no data |  | no data |  | no data |  |
| (-)-SYA 09 | **5** | 6.6 |  | no data |  | no data |  | no data |  |
| Spiperone | **259** | 6.6 |  | no data |  | no data |  | no data |  |
| Citalopram | **147** | 6.5 |  | no data |  | no data |  | no data |  |
| Cimbi-712 | **136** | 6.5 |  | no data |  | no data |  | no data |  |
| Naphyrone | **224** | 6.5 |  | 5.6 |  | no data |  | no data |  |
| mCPP | **203** | 6.5 |  | no data |  | no data |  | no data |  |
| Desmethyldesipramine | **158** | 6.5 |  | 5.5 |  | no data |  | no data |  |
| RWAY,(-) | **251** | 6.5 |  | 5.5 |  | no data |  | no data |  |
| Arpromidine | **121** | 6.5 |  | no data |  | no data |  | no data |  |
| Methylhistaprodifen | **209** | 6.4 |  | no data |  | no data |  | no data |  |
| Nefazodone | **227** | 6.4 |  | no data |  | no data |  | no data |  |
| Dimethylhistaprodifen | **161** | 6.4 |  | no data |  | no data |  | no data |  |
| (+)-7,8-Dimethoxy-3-[4-(naphthalen-2-ylmethyl)piperazin-1-ylmethyl]-3a,4-dihydro-3H-[1]benzopyrano[4,3-c]isoxazole | **10** | 6.3 |  | no data |  | no data |  | no data |  |
| Cimbi-775 | **139** | 6.3 |  | no data |  | no data |  | no data |  |
| Cimbi-772 | **138** | 6.3 |  | no data |  | no data |  | no data |  |
| 2-methoxy-diphenidine | **80** | 6.3 |  | no data |  | no data |  | no data |  |
| UR-AK57 | **282** | 6.2 | 0.5 | 7.6 |  | no data |  | no data |  |
| Mazindol | **202** | 6.2 |  | no data |  | no data |  | no data |  |
| Desmethylcitalopram | **157** | 6.2 |  | no data |  | no data |  | no data |  |
| Pimozide | **239** | 6.2 |  | no data |  | no data |  | no data |  |
| N-(9,10-Dihydro-9-anthracenylmethyl)-3-phenyl-1-propanamine | **220** | 6.1 |  | no data |  | no data |  | no data |  |
| 2-methylhistamine | **81** | 6.1 |  | no data |  | no data |  | no data |  |
| 2-{4-[4-(3-Methoxyphenyl)-1-piperazinyl]butyl}-4-methyl-1,2,4-triazine-3,5(2H,4H)-dione | **76** | 6.1 |  | no data |  | no data |  | no data |  |
| N-(9,10-Dihydro-9-anthracenylmethyl)-4-phenyl-1-butanamine | **221** | 6.0 |  | no data |  | no data |  | no data |  |
| UR-AK64 | **285** | 6.0 | 0.0 | 7.6 |  | no data |  | no data |  |
| [5-Fluoro-2-methyl-1-(4-methanesulfonyl-benzenesulfonyl)-1H-indol-3-yl]-acetic acid | **51** | 6.0 |  | 6.0 |  | 6.0 |  | no data |  |
| SR 147778 | **260** | 6.0 |  | 6.0 |  | 6.0 |  | no data |  |
| (+)-7,8-Dimethoxy-3-[4-(3-methyl-3-phenyl-2(E)-propen-1-yl)piperazin-1-ylmethyl]-3a,4-dihydro-3H-[1]benzopyrano[4,3-c]isoxazole | **8** | 6.0 |  | no data |  | no data |  | no data |  |
| (+)-7,8-Dimethoxy-3-[4-(3-phenyl-2(E)-propen-1-yl)piperazin-1-ylmethyl]-3a,4-dihydro-3H-[1]benzopyrano[4,3-c]isoxazole | **9** | 6.0 |  | no data |  | no data |  | no data |  |
| A-80426 | **111** | 6.0 |  | no data |  | no data |  | no data |  |
| Atomoxetine | **123** | 6.0 |  | no data |  | no data |  | no data |  |
| S-34324 | **252** | 6.0 |  | no data |  | no data |  | no data |  |
| HIS | **181** | 6.0 | 1.0 | 5.9 |  | no data |  | no data |  |
| Haloperidol | **180** | 5.9 | 1.3 | 6.0 |  | inactive |  | inactive |  |
| OCH3-6-APT | **231** | 5.9 |  | no data |  | no data |  | no data |  |
| ATR | **124** | 5.9 |  | no data |  | no data |  | no data |  |
| Histamine | **182** | 5.9 | 2.6 | 7.8 |  | 6.4 | 0.7 | 7.8 | 3.1 |
| CL-6-APT | **149** | 5.9 |  | no data |  | no data |  | no data |  |
| Diphenidine | **163** | 5.9 |  | no data |  | no data |  | no data |  |
| SYA 09 (+) | **13** | 5.8 |  | no data |  | no data |  | no data |  |
| Dimethylamylamine | **160** | 5.8 |  | no data |  | no data |  | no data |  |
| 7-OH-DPAT | **101** | 5.8 |  | no data |  | no data |  | no data |  |
| MDL 11,939 | **205** | 5.8 |  | no data |  | no data |  | no data |  |
| ABT-239 | **112** | 5.8 | 0.0 | 5.2 | 0.0 | 9.3 | 0.1 | no data |  |
| 2-(3-Iodophenyl)histamine | **73** | 5.8 |  | no data |  | no data |  | no data |  |
| 2-(3-Chlorophenyl)histamine | **72** | 5.7 | 0.3 | no data |  | no data |  | no data |  |
| Ciproxifan | **143** | 5.7 |  | no data |  | 7.2 | 0.0 | 5.7 | 0.0 |
| Escitalopram | **167** | 5.7 |  | no data |  | no data |  | no data |  |
| Loperamide | **199** | 5.7 |  | no data |  | no data |  | no data |  |
| 3-methoxy-diphenidine | **87** | 5.7 |  | no data |  | no data |  | no data |  |
| Molindone | **216** | 5.7 |  | no data |  | no data |  | no data |  |
| Cocaine | **153** | 5.7 |  | no data |  | no data |  | no data |  |
| MDMA | **206** | 5.7 | 0.0 | no data |  | no data |  | no data |  |
| pdspTestCompound2 | **234** | 5.7 |  | no data |  | no data |  | no data |  |
| Duloxetine | **165** | 5.6 | 0.0 | no data |  | no data |  | no data |  |
| A-349821 | **110** | 5.6 | 0.0 | no data |  | 9.4 |  | no data |  |
| Pipamperone | **240** | 5.6 |  | no data |  | no data |  | no data |  |
| Cimbi-775 (-) | **3** | 5.6 |  | no data |  | no data |  | no data |  |
| SKF 38393 | **256** | 5.6 |  | no data |  | no data |  | no data |  |
| 8R-lisuride | **105** | 5.6 | 2.6 | no data |  | no data |  | no data |  |
| GT 2016 | **176** | 5.6 |  | 5.1 |  | no data |  | 6.1 |  |
| 1-(3-Methoxy-7,12-dihydro-12-tetraphenyl)methanamine | **68** | 5.6 |  | no data |  | no data |  | no data |  |
| CL-5-APT | **148** | 5.6 |  | no data |  | no data |  | no data |  |
| CF3-6-APT | **133** | 5.6 |  | no data |  | no data |  | no data |  |
| Clobenpropit | **151** | 5.6 |  | 5.2 |  | 7.2 | 1.4 | 7.9 | 0.7 |
| UR-AK22 | **275** | 5.5 | 0.0 | 7.1 |  | no data |  | no data |  |
| A-331440 | **109** | 5.5 |  | no data |  | no data |  | no data |  |
| Methysergide | **210** | 5.5 |  | no data |  | no data |  | no data |  |
| Haldol | **179** | 5.5 |  | no data |  | no data |  | no data |  |
| 5-APT | **97** | 5.5 |  | no data |  | no data |  | no data |  |
| OCH3-5-APT | **230** | 5.5 |  | no data |  | no data |  | no data |  |
| UR-AK68 | **287** | 5.5 | 0.0 | 7.1 |  | no data |  | no data |  |
| A-317920 | **108** | 5.4 |  | no data |  | 7.0 |  | no data |  |
| UR-AK59 | **283** | 5.4 | 0.4 | 7.0 |  | no data |  | no data |  |
| 4-methoxy-diphenidine | **95** | 5.3 |  | no data |  | no data |  | no data |  |
| 2-(2-Thiazolyl)ethanamine | **71** | 5.3 |  | no data |  | no data |  | no data |  |
| 3-(Dimethylamino)fluoranthene | **84** | 5.3 |  | no data |  | no data |  | no data |  |
| 3-Aminofluoranthene | **86** | 5.3 |  | no data |  | no data |  | no data |  |
| NANM (-) | **4** | 5.3 |  | no data |  | no data |  | no data |  |
| Rimcazole | **249** | 5.3 |  | no data |  | no data |  | no data |  |
| Sertraline | **255** | 5.3 |  | no data |  | no data |  | no data |  |
| Amoxapine | **117** | 5.3 |  | no data |  | inactive |  | inactive |  |
| UR-AK24 | **276** | 5.3 | 0.0 | 7.2 |  | no data |  | no data |  |
| 4-(8-Chloro-5H-dibenzo[b,e][1,4]diazepin-11-yl)-1-isopropylpiperazine N-oxide | **91** | 5.2 |  | no data |  | no data |  | no data |  |
| CF3-5-APT | **132** | 5.2 |  | no data |  | no data |  | no data |  |
| UR-AK62 | **284** | 5.2 | 0.0 | 7.2 |  | no data |  | no data |  |
| pdspTestCompound2 | **235** | 5.1 |  | no data |  | no data |  | no data |  |
| Raclopride | **245** | 5.1 |  | no data |  | no data |  | no data |  |
| 4-[3-(4-Chlorophenyl)-3-hydroxy-8-azabicyclo[3.2.1]oct-8-yl]-1-(4-fluorophenyl)-1-butanone | **92** | 5.1 |  | no data |  | no data |  | no data |  |
| 4-[5-trans-hydroxy-5-cis-(4′-chlorophenyl)-2-azabicyclo[3.2.1]octanyl]-4″-fluorobutyrophenone | **93** | 5.1 |  | no data |  | no data |  | no data |  |
| 6-APT | **99** | 5.0 |  | no data |  | no data |  | no data |  |
| Proxyfan | **243** | no data |  | 7.9 |  | 8.3 |  | 7.3 |  |
| Aminopotentidine | **115** | no data |  | 7.8 |  | no data |  | no data |  |
| Famotidine | **168** | no data |  | 7.8 |  | no data |  | no data |  |
| Tiotidine | **266** | no data |  | 7.8 |  | no data |  | no data |  |
| UR-AK46 | **279** | no data |  | 7.4 |  | no data |  | no data |  |
| UR-AK49 | **280** | no data |  | 7.3 |  | no data |  | no data |  |
| UR-AK67 | **286** | no data |  | 7.2 |  | no data |  | no data |  |
| Ranitidine | **248** | no data |  | 7.1 |  | no data |  | no data |  |
| UR-AK51 | **281** | no data |  | 7.0 |  | no data |  | no data |  |
| UR-AK26 | **277** | no data |  | 6.8 |  | no data |  | no data |  |
| Impromidine | **191** | no data |  | 6.6 | 1.3 | 8.3 | 0.4 | 7.7 | 0.3 |
| 2-{4-[4-(7-Methoxy-naphthalen-1-yl)piperazin-1-yl]-butyl}-4-methyl-2H-[12,4]triazine-3,5-dione | **79** | no data |  | 6.5 |  | no data |  | no data |  |
| Dimaprit | **159** | no data |  | 6.5 |  | 6.1 |  | 6.4 |  |
| cimetidine | **140** | no data |  | 6.2 |  | no data |  | no data |  |
| Nantenin | **223** | no data |  | 6.2 |  | no data |  | no data |  |
| Sopromidine R(-) | **257** | no data |  | 6.1 |  | no data |  | no data |  |
| UR-AK41 | **278** | no data |  | 5.9 |  | no data |  | no data |  |
| VUF4742 | **296** | no data |  | 5.8 |  | no data |  | 6.9 |  |
| Burimamide | **127** | no data |  | 5.7 | 2.0 | 8.3 | 0.8 | 7.0 | 0.7 |
| 2-{4-[4-(3-Methoxyphenyl)-1-piperazinyl]butyl}-4-methyl-124-triazine-35(2H4H)-dione | **77** | no data |  | 5.7 |  | no data |  | no data |  |
| Amselamine | **118** | no data |  | 5.6 |  | no data |  | no data |  |
| Pramipexole | **241** | no data |  | 5.6 |  | no data |  | no data |  |
| (3S4S6aS9S10S12aS)-4-(2-bromophenyl)-10-(4-(3-(dimethylamino)prop-1-ynyl)phenyl)-39-dimethyloctahydrodipyrido[12-a:1'2'-d]pyrazine-612(6aH12aH)-dione | **27** | no data |  | 5.6 |  | no data |  | no data |  |
| Sopromidine S(+) | **258** | no data |  | 5.5 |  | no data |  | no data |  |
| MH.MZ | **211** | no data |  | 5.4 |  | no data |  | no data |  |
| VUF4617 | **290** | no data |  | 5.4 |  | no data |  | 7.9 |  |
| 4-methylhistamine | **96** | no data |  | 5.4 | 2.2 | no data |  | 7.4 | 0.3 |
| GT 2227 | **177** | no data |  | 5.3 |  | no data |  | no data |  |
| (3S4S6aS9S10S12aS)-410-bis(4-(3-(dimethylamino)prop-1-ynyl)phenyl)-39-dimethyloctahydrodipyrido[12-a:1'2'-d]pyrazine-612(6aH12aH)-dione | **28** | no data |  | 5.3 |  | no data |  | no data |  |
| Amthamine | **119** | no data |  | 5.2 | 0.1 | no data |  | no data |  |
| {2-[2-(2-(R)-Methylpyrrolidin-1-yl)ethyl]benzofuran-5-yl}(5-nitropyridin-2-yl)amine | **54** | no data |  | no data |  | 10.3 |  | no data |  |
| {2-[2-(2-(R)-Methylpyrrolidin-1-yl)ethyl]benzofuran-5-yl}pyrimidin-5-ylamine | **58** | no data |  | no data |  | 10.1 |  | no data |  |
| {2-[2-(2-(R)-Methylpyrrolidin-1-yl)ethyl]benzofuran-5-yl}pyrazin-2-ylamine | **56** | no data |  | no data |  | 10.0 |  | no data |  |
| 4-({2-[2-(2-(R)-Methylpyrrolidin-1-yl)ethyl]benzofuran-5-ylmethyl}amino)pyridine-2,6-dicarbonitrile | **88** | no data |  | no data |  | 9.9 |  | no data |  |
| {2-[2-(2-(R)-Methylpyrrolidin-1-yl)ethyl]benzofuran-5-yl}(3-nitropyridin-2-yl)amine | **53** | no data |  | no data |  | 9.9 |  | no data |  |
| {2-[2-(2-(R)-Methylpyrrolidin-1-yl)ethyl]benzofuran-5-ylmethyl}(5-nitrothiazol-2-yl)amine | **62** | no data |  | no data |  | 9.7 |  | no data |  |
| {2-[2-(2-(R)-Methylpyrrolidin-1-yl)ethyl]benzofuran-5-yl}(2-nitrophenyl)amine | **52** | no data |  | no data |  | 9.7 |  | no data |  |
| 4-{2-[2-(2-(R)-Methylpyrrolidin-1-yl)ethyl]benzofuran-5-ylamino}benzonitrile | **94** | no data |  | no data |  | 9.6 |  | no data |  |
| {2-[2-(2-(R)-Methylpyrrolidin-1-yl)ethyl]benzofuran-5-ylmethyl}pyrazin-2-ylamine | **63** | no data |  | no data |  | 9.5 |  | no data |  |
| 3-{2-[2-(2-(R)-Methylpyrrolidin-1-yl)ethyl]benzofuran-5-ylamino}benzonitrile | **85** | no data |  | no data |  | 9.5 |  | no data |  |
| {2-[2-(2-(R)-Methylpyrrolidin-1-yl)ethyl]benzofuran-5-ylmethyl}(5-nitropyridin-2-yl)amine | **61** | no data |  | no data |  | 9.5 |  | no data |  |
| (6-Chloropyridazin-3-yl){2-[2-(2-methylpyrrolidin-1-yl)ethyl]benzofuran-5-ylmethyl}amine | **45** | no data |  | no data |  | 9.5 |  | no data |  |
| {2-[2-(2-(R)-Methylpyrrolidin-1-yl)ethyl]benzofuran-5-yl}pyrimidin-2-ylamine | **57** | no data |  | no data |  | 9.5 |  | no data |  |
| {2-[2-(2-(R)-Methylpyrrolidin-1-yl)ethyl]benzofuran-5-ylmethyl}(2-nitrophenyl)amine | **59** | no data |  | no data |  | 9.5 |  | no data |  |
| (5-Ethylpyrimidin-2-yl){2-[2-(2-(R)-methylpyrrolidin-1-yl)ethyl]benzofuran-5-ylmethyl}amine | **39** | no data |  | no data |  | 9.5 |  | no data |  |
| 6-({2-[2-(2-(R)-Methylpyrrolidin-1-yl)ethyl]benzofuran-5-ylmethyl}amino)nicotinonitrile | **98** | no data |  | no data |  | 9.5 |  | no data |  |
| 3-({2-[2-(2-(R)-Methylpyrrolidin-1-yl)ethyl]benzofuran-5-ylmethyl}amino)benzonitrile | **82** | no data |  | no data |  | 9.4 |  | no data |  |
| {2-[2-(2-(R)-Methylpyrrolidin-1-yl)ethyl]benzofuran-5-ylmethyl}(3-nitropyridin-2-yl)amine | **60** | no data |  | no data |  | 9.4 |  | no data |  |
| {2-[2-(2-(R)-Methylpyrrolidin-1-yl)ethyl]benzofuran-5-yl}(5-trifluoromethylpyridin-2-yl)amine | **55** | no data |  | no data |  | 9.3 |  | no data |  |
| JNJ-5207852 | **195** | no data |  | no data |  | 9.2 |  | no data |  |
| Immepip | **188** | no data |  | no data |  | 9.2 | 0.6 | 7.7 | 0.3 |
| (5-Ethylpyrimidin-2-yl){2-[2-(2-(R)-methylpyrrolidin-1-yl)ethyl]benzofuran-5-yl}amine | **38** | no data |  | no data |  | 9.2 |  | no data |  |
| Iodoproxyfan | **193** | no data |  | no data |  | 9.2 |  | 7.9 |  |
| {2-[2-(2-(R)-Methylpyrrolidin-1-yl)ethyl]benzofuran-5-ylmethyl}pyrimidin-5-ylamine | **65** | no data |  | no data |  | 9.2 |  | no data |  |
| RAMH | **247** | no data |  | no data |  | 9.2 |  | no data |  |
| (3,5-Dinitropyridin-2-yl){2-[2-(2-(R)-methylpyrrolidin-1-yl)ethyl]benzofuran-5-ylmethyl}amine | **25** | no data |  | no data |  | 9.1 |  | no data |  |
| {2-[2-(2-Methylpyrrolidin-1-yl)ethyl]benzofuran-5-ylmethyl}(4-nitrophenyl)amine | **66** | no data |  | no data |  | 9.1 |  | no data |  |
| 2-({2-[2-(2-(R)-Methylpyrrolidin-1-yl)ethyl]benzofuran-5-ylmethyl}amino)nicotinonitrile | **70** | no data |  | no data |  | 9.1 |  | no data |  |
| 2,2,2-Trifluoro-1-[4-({2-[2-(2-methylpyrrolidin-1-yl)ethyl]benzofuran-5-ylmethyl}amino)phenyl]ethanone | **75** | no data |  | no data |  | 9.1 |  | no data |  |
| Immethridine | **189** | no data |  | no data |  | 9.1 |  | 6.6 |  |
| {2-[2-(2-(R)-Methylpyrrolidin-1-yl)ethyl]benzofuran-5-ylmethyl}pyrimidin-2-ylamine | **64** | no data |  | no data |  | 9.1 |  | no data |  |
| Thioperamide | **263** | no data |  | no data |  | 9.0 | 0.5 | 7.3 | 0.7 |
| Methimmepip | **208** | no data |  | no data |  | 9.0 |  | 5.7 |  |
| 3-({2-[2-(2-(R)-Methylpyrrolidin-1-yl)ethyl]benzofuran-5-ylmethyl}amino)pyrazine-2-carbonitrile | **83** | no data |  | no data |  | 9.0 |  | no data |  |
| (5-Bromopyrimidin-2-yl){2-[2-(2-(R)-methylpyrrolidin-1-yl)ethyl]benzofuran-5-ylmethyl}amine | **36** | no data |  | no data |  | 8.9 |  | no data |  |
| N-methylhistamine | **228** | no data |  | no data |  | 8.8 | 0.7 | 7.6 |  |
| VUF5202 | **299** | no data |  | no data |  | 8.6 |  | no data |  |
| R- α -methylhistamine | **246** | no data |  | no data |  | 8.6 | 1.1 | 6.5 | 0.2 |
| GT 2331 | **178** | no data |  | no data |  | 8.6 |  | no data |  |
| 6-Methyl-2-{2-[2-(2-(R)-methylpyrrolidin-1-yl)ethyl]benzofuran-5-ylamino}nicotinonitrile | **100** | no data |  | no data |  | 8.5 |  | no data |  |
| Imbutamine | **185** | no data |  | no data |  | 8.4 |  | 8.0 |  |
| N- α -methylhistamine | **222** | no data |  | no data |  | 8.4 |  | 6.5 |  |
| α -methylhistamine-R | **113** | no data |  | no data |  | 8.4 | 1.0 | 6.8 | 0.2 |
| Impentamine | **190** | no data |  | no data |  | 8.3 | 0.0 | 6.6 |  |
| Cipralisant | **142** | no data |  | no data |  | 8.3 |  | 7.3 | 0.0 |
| Imetit | **186** | no data |  | no data |  | 8.3 | 0.8 | 8.2 | 3.1 |
| VUF5300 | **301** | no data |  | no data |  | 8.1 |  | no data |  |
| VUF4903 | **297** | no data |  | no data |  | 8.0 |  | no data |  |
| VUF8328 | **302** | no data |  | no data |  | 7.9 | 0.0 | 8.0 |  |
| VUF4904 | **298** | no data |  | no data |  | 7.9 |  | no data |  |
| VUF5207 | **300** | no data |  | no data |  | 7.8 |  | no data |  |
| 4-(3-piperidin-1-yl-propoxy)-benzonitrile | **89** | no data |  | no data |  | 7.6 | 0.0 | no data |  |
| Homohistamine | **183** | no data |  | no data |  | 7.3 |  | 7.5 |  |
| S-α -methylhistamine | **114** | no data |  | no data |  | 7.2 |  | 5.4 |  |
| Iodophenpropit | **192** | no data |  | no data |  | 6.9 | 0.1 | 7.8 | 0.2 |
| A-304121 | **107** | no data |  | no data |  | 6.1 |  | no data |  |
| JNJ 7777120 | **194** | no data |  | no data |  | 5.3 |  | 7.8 | 0.0 |
| Mitragynine | **214** | no data |  | no data |  | 5.1 |  | no data |  |
| (5-Chloro-1H-indol-2-yl)-(4-methyl-piperazin-1-yl)-methanone | **37** | no data |  | no data |  | no data |  | 8.4 |  |
| (7-Methyl-1H-indol-2-yl)-(4-methyl-piperazin-1-yl)-methanone | **49** | no data |  | no data |  | no data |  | 8.2 |  |
| VUF4683 | **293** | no data |  | no data |  | no data |  | 8.1 |  |
| (5-Bromo-1H-indol-2-yl)-(4-methyl-piperazin-1-yl)-methanone | **35** | no data |  | no data |  | no data |  | 8.1 |  |
| (7-Amino-1H-indol-2-yl)-(4-methyl-piperazin-1-yl)-methanone | **46** | no data |  | no data |  | no data |  | 8.1 |  |
| VUF4682 | **292** | no data |  | no data |  | no data |  | 8.0 |  |
| (5,7- Dichloro-1H-indol-2-yl)-(4-methyl-piperazin-1-yl)-methanone | **31** | no data |  | no data |  | no data |  | 8.0 |  |
| VUF4616 | **289** | no data |  | no data |  | no data |  | 7.9 |  |
| (5,7-Difluoro-1H-indol-2-yl)-(4-methyl-piperazin-1-yl)-methanone | **32** | no data |  | no data |  | no data |  | 7.9 |  |
| (5-Amino-1H-indol-2-yl)-(4-methyl-piperazin-1-yl)-methanone | **34** | no data |  | no data |  | no data |  | 7.8 |  |
| (5-Fluoro-1H-indol-2-yl)-(4-methyl-piperazin-1-yl)-methanone | **40** | no data |  | no data |  | no data |  | 7.8 |  |
| (1H-Indol-2-yl)-(4-methyl-piperazin-1-yl)-methanone | **15** | no data |  | no data |  | no data |  | 7.8 |  |
| (7-Chloro-1H-indol-2-yl)-(4-methyl-piperazin-1-yl)-methanone | **48** | no data |  | no data |  | no data |  | 7.7 |  |
| (5-Hydroxy-1H-indol-2-yl)-(4-methyl-piperazin-1-yl)-methanone | **41** | no data |  | no data |  | no data |  | 7.6 |  |
| VUF4614 | **288** | no data |  | no data |  | no data |  | 7.6 |  |
| VUF4681 | **291** | no data |  | no data |  | no data |  | 7.6 |  |
| (5,7-Dimethyl-1H-indol-2-yl)-(4-methyl-piperazin-1-yl)-methanone | **33** | no data |  | no data |  | no data |  | 7.5 |  |
| (4-Bromo-1H-indol-2-yl)-(4-methyl-piperazin-1-yl)-methanone | **29** | no data |  | no data |  | no data |  | 7.5 |  |
| (1H-Indol-2-yl)-(piperazin-1-yl)-methanone | **17** | no data |  | no data |  | no data |  | 7.4 |  |
| (5-Methyl-1H-indol-2-yl)-(4-methyl-piperazin-1-yl)-methanone | **43** | no data |  | no data |  | no data |  | 7.3 |  |
| VUF4686 | **294** | no data |  | no data |  | no data |  | 7.3 |  |
| (7-Bromo-1H-indol-2-yl)-(4-methyl-piperazin-1-yl)-methanone | **47** | no data |  | no data |  | no data |  | 7.2 |  |
| VUF4687 | **295** | no data |  | no data |  | no data |  | 7.2 |  |
| Cinnarizine | **141** | no data |  | no data |  | no data |  | 6.8 |  |
| (6-Bromo-1H-indol-2-yl)-(4-methyl-piperazin-1-yl)-methanone | **44** | no data |  | no data |  | no data |  | 6.8 |  |
| +-(1H-Indol-2-yl)-(3-methyl-piperazin-1-yl)-methanone | **67** | no data |  | no data |  | no data |  | 6.7 |  |
| (4-Ethyl-piperazin-1-yl)-(1H-indol-2-yl)-methanone | **30** | no data |  | no data |  | no data |  | 6.6 |  |
| Clemizole | **150** | no data |  | no data |  | no data |  | 6.4 |  |
| (5-Methoxy-1H-indol-2-yl)-(4-methyl-piperazin-1-yl)-methanone | **42** | no data |  | no data |  | no data |  | 5.5 |  |
| (3S,4S,6aS,9S,10S,12aS)-4,10-bis(4-(3-(dimethylamino)prop-1-ynyl)phenyl)-3,9-dimethyloctahydrodipyrido[1,2-a:1',2'-d]pyrazine-6,12(6aH,12aH)-dione | **26** | no data |  | no data |  | no data |  | 5.5 |  |
| Pheniramine | **238** | no data |  | no data |  | no data |  | 5.4 |  |
| (1H-Indol-2-yl)-(4-phenethyl-piperazin-1-yl)-methanone | **16** | no data |  | no data |  | no data |  | 5.2 |  |
| MRS5698 | **217** | no data |  | no data |  | no data |  | 5.0 |  |

^3^H

***Supplemental Table 2:*** *Overview of radioligands or experimental conditions used for the determination of binding affinities at the hH_x_R. For each ligand derived from the PDSP Ki database, the corresponding radioligands used in the underlying experiments are listed separately for the hH_1_R – hH_4_R. If multiple radioligands are listed, multiple experimental studies were available that determined the binding affinity for the respective hH_x_R. Entries labelled as “functional” indicate data derived from functional assays rather than binding experiments, while “undefined” denotes missing or unspecified methodological information. Ligands are sorted in ascending order of their ligand number.*

| **Ligand** | **Ligand no.** | **Radioligand hH_1_R** | **Radioligand hH_2_R** | **Radioligand hH_3_R** | **Radioligand hH_4_R** |
| --- | --- | --- | --- | --- | --- |
| (-)-(2RS,3aRS,12bSR)-2-N,N-Dimethylaminomethyl-11-fluoro-2,3,3a,12b-tetrahydrodibenzo[b,f]furo[2,3-d]tiepin | **1** | [^3^H] -mepyramine |  |  |  |
| Cimbi-772 (-) | **2** | [^3^H]mepyramine |  |  |  |
| Cimbi-775 (-) | **3** | [^3^H]mepyramine |  |  |  |
| NANM (-) | **4** | [^3^H]mepyramine |  |  |  |
| SYA 09 (-) | **5** | [^3^H]mepyramine |  |  |  |
| (+)-(2RS,3aRS,12bSR)-2-N,N-Dimethylaminomethyl-11-fluoro-2,3,3a,12b-tetrahydrodibenzo[b,f]furo[2,3-d]tiepin | **6** | [^3^H]mepyramine |  |  |  |
| (+)-7,8-Dimethoxy-3-[4-(2-methyl-3-phenyl-2(E)-propen-1-yl)piperazin-1-ylmethyl]-3a,4-dihydro-^3^H-[1]benzopyrano[4,3-c]isoxazole | **7** | [^3^H]mepyramine |  |  |  |
| (+)-7,8-Dimethoxy-3-[4-(3-methyl-3-phenyl-2(E)-propen-1-yl)piperazin-1-ylmethyl]-3a,4-dihydro-^3^H-[1]benzopyrano[4,3-c]isoxazole | **8** | [^3^H]mepyramine |  |  |  |
| (+)-7,8-Dimethoxy-3-[4-(3-phenyl-2(E)-propen-1-yl)piperazin-1-ylmethyl]-3a,4-dihydro-^3^H-[1]benzopyrano[4,3-c]isoxazole | **9** | [^3^H]mepyramine |  |  |  |
| (+)-7,8-Dimethoxy-3-[4-(naphthalen-2-ylmethyl)piperazin-1-ylmethyl]-3a,4-dihydro-^3^H-[1]benzopyrano[4,3-c]isoxazole | **10** | [^3^H]mepyramine |  |  |  |
| Cimbi-772 (+) | **11** | [^3^H]mepyramine |  |  |  |
| Cimbi-775 (+) | **12** | [^3^H]mepyramine |  |  |  |
| SYA 09 (+) | **13** | [^3^H]mepyramine |  |  |  |
| BMY-1402 (+/-) | **14** | [^3^H]mepyramine |  |  |  |
| (1H-Indol-2-yl)-(4-methyl-piperazin-1-yl)-methanone | **15** |  |  |  | [^3^H]histamine |
| (1H-Indol-2-yl)-(4-phenethyl-piperazin-1-yl)-methanone | **16** |  |  |  | [^3^H]histamine |
| (1H-Indol-2-yl)-(piperazin-1-yl)-methanone | **17** |  |  |  | [^3^H]histamine |
| (2RS,3aRS,12bSR)-2-N,N-Dimethylaminomethyl-11-bromo-2,3,3a,12b-tetrahydrodibenzo[b,f]furo[2,3-d]oxepin | **18** | [^3^H]mepyramine |  |  |  |
| (2RS,3aRS,12bSR)-2-N,N-Dimethylaminomethyl-11-chloro-2,3,3a,12b-tetrahydrodibenzo[b,f]furo[2,3-d]oxepin | **19** | [^3^H]mepyramine |  |  |  |
| (2RS,3aRS,12bSR)-2-N,N-Dimethylaminomethyl-11-fluoro-2,3,3a,12b-tetrahydrodibenzo[b,f]furo[2,3-d]oxepin | **20** | [^3^H]mepyramine |  |  |  |
| (2RS,3aRS,12bSR)-2-N,N-Dimethylaminomethyl-11-fluoro-2,3,3a,12b-tetrahydrodibenzo[b,f]furo[2,3-d]tiepin | **21** | [^3^H]mepyramine |  |  |  |
| (2SR,3aRS,12bSR)-2-N,N-Dimethylaminomethyl-11-chloro-2,3,3a,12b-tetrahydrodibenzo[b,f]furo[2,3-d]oxepin | **22** | [^3^H]mepyramine |  |  |  |
| (2SR,3aRS,12bSR)-2-N,N-Dimethylaminomethyl-11-fluoro-2,3,3a,12b-tetrahydrodibenzo[b,f]furo[2,3-d]oxepin | **23** | [^3^H]mepyramine |  |  |  |
| ***Ligand*** | ***Ligand no.*** | ***Radioligand hH_1_R*** | ***Radioligand hH_2_R*** | ***Radioligand hH_3_R*** | ***Radioligand hH_4_R*** |
| (2SR,3aRS,12bSR)-2-N,N-Dimethylaminomethyl-11-fluoro-2,3,3a,12b-tetrahydrodibenzo[b,f]furo[2,3-d]tiepin | **24** | [^3^H]mepyramine |  |  |  |
| (3,5-Dinitropyridin-2-yl){2-[2-(2-(R)-methylpyrrolidin-1-yl)ethyl]benzofuran-5-ylmethyl}amine | **25** |  |  | [^3^H]RAMH |  |
| (3S,4S,6aS,9S,10S,12aS)-4,10-bis(4-(3-(dimethylamino)prop-1-ynyl)phenyl)-3,9-dimethyloctahydrodipyrido[1,2-a:1',2'-d]pyrazine-6,12(6aH,12aH)-dione | **26** |  |  |  | [^3^H]histamine |
| (3S4S6aS9S10S12aS)-4-(2-bromophenyl)-10-(4-(3-(dimethylamino)prop-1-ynyl)phenyl)-39-dimethyloctahydrodipyrido[12-a:1'2'-d]pyrazine-612(6aH12aH)-dione | **27** |  | [^3^H]tiotidine |  |  |
| (3S4S6aS9S10S12aS)-410-bis(4-(3-(dimethylamino)prop-1-ynyl)phenyl)-39-dimethyloctahydrodipyrido[12-a:1'2'-d]pyrazine-612(6aH12aH)-dione | **28** |  | [^3^H]tiotidine |  |  |
| (4-Bromo-1H-indol-2-yl)-(4-methyl-piperazin-1-yl)-methanone | **29** |  |  |  | [^3^H]histamine |
| (4-Ethyl-piperazin-1-yl)-(1H-indol-2-yl)-methanone | **30** |  |  |  | [^3^H]histamine |
| (5,7- Dichloro-1H-indol-2-yl)-(4-methyl-piperazin-1-yl)-methanone | **31** |  |  |  | [^3^H]histamine |
| (5,7-Difluoro-1H-indol-2-yl)-(4-methyl-piperazin-1-yl)-methanone | **32** |  |  |  | [^3^H]histamine |
| (5,7-Dimethyl-1H-indol-2-yl)-(4-methyl-piperazin-1-yl)-methanone | **33** |  |  |  | [^3^H]histamine |
| (5-Amino-1H-indol-2-yl)-(4-methyl-piperazin-1-yl)-methanone | **34** |  |  |  | [^3^H]histamine |
| (5-Bromo-1H-indol-2-yl)-(4-methyl-piperazin-1-yl)-methanone | **35** |  |  |  | [^3^H]histamine |
| (5-Bromopyrimidin-2-yl){2-[2-(2-(R)-methylpyrrolidin-1-yl)ethyl]benzofuran-5-ylmethyl}amine | **36** |  |  | [^3^H]RAMH |  |
| (5-Chloro-1H-indol-2-yl)-(4-methyl-piperazin-1-yl)-methanone | **37** |  |  |  | [^3^H]histamine |
| (5-Ethylpyrimidin-2-yl){2-[2-(2-(R)-methylpyrrolidin-1-yl)ethyl]benzofuran-5-yl}amine | **38** |  |  | [^3^H]RAMH |  |
| (5-Ethylpyrimidin-2-yl){2-[2-(2-(R)-methylpyrrolidin-1-yl)ethyl]benzofuran-5-ylmethyl}amine | **39** |  |  | [^3^H]RAMH |  |
| (5-Fluoro-1H-indol-2-yl)-(4-methyl-piperazin-1-yl)-methanone | **40** |  |  |  | [^3^H]histamine |
| (5-Hydroxy-1H-indol-2-yl)-(4-methyl-piperazin-1-yl)-methanone | **41** |  |  |  | [^3^H]histamine |
| (5-Methoxy-1H-indol-2-yl)-(4-methyl-piperazin-1-yl)-methanone | **42** |  |  |  | [^3^H]histamine |
| (5-Methyl-1H-indol-2-yl)-(4-methyl-piperazin-1-yl)-methanone | **43** |  |  |  | [^3^H]histamine |
| (6-Bromo-1H-indol-2-yl)-(4-methyl-piperazin-1-yl)-methanone | **44** |  |  |  | [^3^H]histamine |
| (6-Chloropyridazin-3-yl){2-[2-(2-methylpyrrolidin-1-yl)ethyl]benzofuran-5-ylmethyl}amine | **45** |  |  | [^3^H]RAMH |  |
| (7-Amino-1H-indol-2-yl)-(4-methyl-piperazin-1-yl)-methanone | **46** |  |  |  | [^3^H]histamine |
| (7-Bromo-1H-indol-2-yl)-(4-methyl-piperazin-1-yl)-methanone | **47** |  |  |  | [^3^H]histamine |
| (7-Chloro-1H-indol-2-yl)-(4-methyl-piperazin-1-yl)-methanone | **48** |  |  |  | [^3^H]histamine |
| (7-Methyl-1H-indol-2-yl)-(4-methyl-piperazin-1-yl)-methanone | **49** |  |  |  | [^3^H]histamine |
| R-Chlopheniramine | **50** | [^3^H]mepyramine |  |  |  |
| [5-Fluoro-2-methyl-1-(4-methanesulfonyl-benzenesulfonyl)-1H-indol-3-yl]-acetic acid | **51** | undefined | undefined | undefined |  |
| {2-[2-(2-(R)-Methylpyrrolidin-1-yl)ethyl]benzofuran-5-yl}(2-nitrophenyl)amine | **52** |  |  | [^3^H]RAMH |  |
| {2-[2-(2-(R)-Methylpyrrolidin-1-yl)ethyl]benzofuran-5-yl}(3-nitropyridin-2-yl)amine | **53** |  |  | [^3^H]RAMH |  |
| {2-[2-(2-(R)-Methylpyrrolidin-1-yl)ethyl]benzofuran-5-yl}(5-nitropyridin-2-yl)amine | **54** |  |  | [^3^H]RAMH |  |
| {2-[2-(2-(R)-Methylpyrrolidin-1-yl)ethyl]benzofuran-5-yl}(5-trifluoromethylpyridin-2-yl)amine | **55** |  |  | [^3^H]RAMH |  |
| {2-[2-(2-(R)-Methylpyrrolidin-1-yl)ethyl]benzofuran-5-yl}pyrazin-2-ylamine | **56** |  |  | [^3^H]RAMH |  |
| {2-[2-(2-(R)-Methylpyrrolidin-1-yl)ethyl]benzofuran-5-yl}pyrimidin-2-ylamine | **57** |  |  | [^3^H]RAMH |  |
| {2-[2-(2-(R)-Methylpyrrolidin-1-yl)ethyl]benzofuran-5-yl}pyrimidin-5-ylamine | **58** |  |  | [^3^H]RAMH |  |
| {2-[2-(2-(R)-Methylpyrrolidin-1-yl)ethyl]benzofuran-5-ylmethyl}(2-nitrophenyl)amine | **59** |  |  | [^3^H]RAMH |  |
| {2-[2-(2-(R)-Methylpyrrolidin-1-yl)ethyl]benzofuran-5-ylmethyl}(3-nitropyridin-2-yl)amine | **60** |  |  | [^3^H]RAMH |  |
| {2-[2-(2-(R)-Methylpyrrolidin-1-yl)ethyl]benzofuran-5-ylmethyl}(5-nitropyridin-2-yl)amine | **61** |  |  | [^3^H]RAMH |  |
| {2-[2-(2-(R)-Methylpyrrolidin-1-yl)ethyl]benzofuran-5-ylmethyl}(5-nitrothiazol-2-yl)amine | **62** |  |  | [^3^H]RAMH |  |
| {2-[2-(2-(R)-Methylpyrrolidin-1-yl)ethyl]benzofuran-5-ylmethyl}pyrazin-2-ylamine | **63** |  |  | [^3^H]RAMH |  |
| ***Ligand*** | ***Ligand no.*** | ***Radioligand hH_1_R*** | ***Radioligand hH_2_R*** | ***Radioligand hH_3_R*** | ***Radioligand hH_4_R*** |
| {2-[2-(2-(R)-Methylpyrrolidin-1-yl)ethyl]benzofuran-5-ylmethyl}pyrimidin-2-ylamine | **64** |  |  | [^3^H]RAMH |  |
| {2-[2-(2-(R)-Methylpyrrolidin-1-yl)ethyl]benzofuran-5-ylmethyl}pyrimidin-5-ylamine | **65** |  |  | [^3^H]RAMH |  |
| {2-[2-(2-Methylpyrrolidin-1-yl)ethyl]benzofuran-5-ylmethyl}(4-nitrophenyl)amine | **66** |  |  | [^3^H]RAMH |  |
| (+/-)(1H-Indol-2-yl)-(3-methyl-piperazin-1-yl)-methanone | **67** |  |  |  | [^3^H]histamine |
| 1-(3-Methoxy-7,12-dihydro-12-tetraphenyl)methanamine | **68** | [^3^H]mepyramine |  |  |  |
| 11-(Piperazin-1-yl)-5H-dibenzo[b,e][1,4]diazepine | **69** | [^3^H]mepyramine |  |  |  |
| 2-({2-[2-(2-(R)-Methylpyrrolidin-1-yl)ethyl]benzofuran-5-ylmethyl}amino)nicotinonitrile | **70** |  |  | [^3^H]RAMH |  |
| 2-(2-Thiazolyl)ethanamine | **71** | [^3^H]mepyramine |  |  |  |
| 2-(3-Chlorophenyl)histamine | **72** | [^3^H]mepyramine  , [^3^H]histamine |  |  |  |
| 2-(3-Iodophenyl)histamine | **73** | [^3^H]mepyramine |  |  |  |
| 2-(Dimethylamino)tetralin | **74** | [^3^H]mepyramine |  |  |  |
| 2,2,2-Trifluoro-1-[4-({2-[2-(2-methylpyrrolidin-1-yl)ethyl]benzofuran-5-ylmethyl}amino)phenyl]ethanone | **75** |  |  | [^3^H]RAMH |  |
| 2-{4-[4-(3-Methoxyphenyl)-1-piperazinyl]butyl}-4-methyl-1,2,4-triazine-3,5(2H,4H)-dione | **76** | [^3^H]mepyramine |  |  |  |
| 2-{4-[4-(3-Methoxyphenyl)-1-piperazinyl]butyl}-4-methyl-124-triazine-35(2H4H)-dione | **77** |  | [^3^H]cimetidine |  |  |
| 2-{4-[4-(7-Methoxy-naphthalen-1-yl)piperazin-1-yl]-butyl}-4-methyl-2H-[1,2,4]triazine-3,5-dione | **78** | [^3^H]mepyramine |  |  |  |
| 2-{4-[4-(7-Methoxy-naphthalen-1-yl)piperazin-1-yl]-butyl}-4-methyl-2H-[12,4]triazine-3,5-dione | **79** |  | [^3^H]tiotidine |  |  |
| 2-methoxy-diphenidine | **80** | [^3^H]mepyramine |  |  |  |
| 2-methylhistamine | **81** | [^3^H]histamine |  |  |  |
| 3-({2-[2-(2-(R)-Methylpyrrolidin-1-yl)ethyl]benzofuran-5-ylmethyl}amino)benzonitrile | **82** |  |  | [^3^H]RAMH |  |
| 3-({2-[2-(2-(R)-Methylpyrrolidin-1-yl)ethyl]benzofuran-5-ylmethyl}amino)pyrazine-2-carbonitrile | **83** |  |  | [^3^H]RAMH |  |
| 3-(Dimethylamino)fluoranthene | **84** | [^3^H]mepyramine |  |  |  |
| 3-{2-[2-(2-(R)-Methylpyrrolidin-1-yl)ethyl]benzofuran-5-ylamino}benzonitrile | **85** |  |  | [^3^H]RAMH |  |
| 3-Aminofluoranthene | **86** | [^3^H]mepyramine |  |  |  |
| 3-methoxy-diphenidine | **87** | [^3^H]mepyramine |  |  |  |
| 4-({2-[2-(2-(R)-Methylpyrrolidin-1-yl)ethyl]benzofuran-5-ylmethyl}amino)pyridine-2,6-dicarbonitrile | **88** |  |  | [^3^H]RAMH |  |
| 4-(3-piperidin-1-yl-propoxy)-benzonitrile | **89** |  |  | [^3^H]histamine,  [^3^H]RAMH |  |
| 4-(8-Chloro-5H-dibenzo[b,e][1,4]diazepin-11-yl)-1-ethylpiperazine N-oxide | **90** | [^3^H]mepyramine |  |  |  |
| 4-(8-Chloro-5H-dibenzo[b,e][1,4]diazepin-11-yl)-1-isopropylpiperazine N-oxide | **91** | [^3^H]mepyramine |  |  |  |
| 4-[3-(4-Chlorophenyl)-3-hydroxy-8-azabicyclo[3.2.1]oct-8-yl]-1-(4-fluorophenyl)-1-butanone | **92** | [^3^H]mepyramine |  |  |  |
| 4-[5-trans-hydroxy-5-cis-(4′-chlorophenyl)-2-azabicyclo[3.2.1]octanyl]-4″-fluorobutyrophenone | **93** | [^3^H]mepyramine |  |  |  |
| 4-{2-[2-(2-(R)-Methylpyrrolidin-1-yl)ethyl]benzofuran-5-ylamino}benzonitrile | **94** |  |  | [^3^H]RAMH |  |
| 4-methoxy-diphenidine | **95** | [^3^H]mepyramine |  |  |  |
| 4-methylhistamine | **96** |  | [^3^H]histamine, undefined |  | [^3^H]JNJ 7777120, [^125^I]iodophen-propit, [^3^H]histamine |
| 5-APT | **97** | [^3^H]mepyramine |  |  |  |
| 6-({2-[2-(2-(R)-Methylpyrrolidin-1-yl)ethyl]benzofuran-5-ylmethyl}amino)nicotinonitrile | **98** |  |  | [^3^H]RAMH |  |
| 6-APT | **99** | [^3^H]mepyramine |  |  |  |
| 6-Methyl-2-{2-[2-(2-(R)-methylpyrrolidin-1-yl)ethyl]benzofuran-5-ylamino}nicotinonitrile | **100** |  |  | [^3^H]RAMH |  |
| 7-OH-DPAT | **101** | [^3^H]mepyramine |  |  |  |
| 8-Chloro-11-(4-ethylpiperazin-1-yl)-5H-dibenzo[b,e][1,4]diazepine | **102** | [^3^H]mepyramine |  |  |  |
| ***Ligand*** | ***Ligand no.*** | ***Radioligand hH_1_R*** | ***Radioligand hH_2_R*** | ***Radioligand hH_3_R*** | ***Radioligand hH_4_R*** |
| 8-Chloro-11-(4-isopropylpiperazin-1-yl)-5H-dibenzo[b,e][1,4]diazepine | **103** | [^3^H]mepyramine |  |  |  |
| 8-Chloro-11-[4-(1,1-dideutrioethyl)piperazin-1-yl]-5H-dibenzo[b,e][1,4]diazepine | **104** | [^3^H]mepyramine |  |  |  |
| 8R-lisuride | **105** | [^3^H]mepyramine  , [^3^H]histamine |  |  |  |
| 9-OH-risperidone | **106** | [^3^H]mepyramine | [^3^H]tiotidine |  | [^3^H]histamine |
| A-304121 | **107** |  |  | [^3^H]NAMH |  |
| A-317920 | **108** | [^3^H]mepyramine |  | [^3^H]NAMH |  |
| A-331440 | **109** | [^3^H]mepyramine |  |  |  |
| A-349821 | **110** | [^3^H]mepyramine  , [^3^H]RAMH |  | [^3^H]RAMH |  |
| A-80426 | **111** | [^3^H]mepyramine |  |  |  |
| ABT-239 | **112** | [^3^H]RAMH ,  [^3^H]mepyramine | [^3^H]RAMH ,  [^3^H]tiotidine | [^3^H]RAMH |  |
| R-α-methylhistamine | **113** |  |  | [^3^H]RAMH ,  [^3^H]histamine | [^3^H]NAMH, [^3^H]histamine |
| S-α-methylhistamine | **114** |  |  | [^3^H]histamine | [^3^H]NAMH |
| Aminopotentidine | **115** |  | [^125^I]iodoamino-potentidine |  |  |
| Amitriptylin | **116** | [^3^H]mepyramine, [^3^H]histamine |  |  | [^3^H]mepyramine |
| Amoxapine | **117** | [^3^H]histamine |  |  |  |
| Amselamine | **118** |  | [^3^H]histamine |  |  |
| Amthamine | **119** |  | [^125^I]iodoamino-potentidine,  [^3^H]histamine |  |  |
| Aripiprazole | **120** | [^3^H]mepyramine |  |  |  |
| Arpromidine | **121** | [^3^H]mepyramine |  |  |  |
| Astemizole | **122** | [^3^H]mepyramine |  |  |  |
| Atomoxetine | **123** | [^3^H]mepyramine |  |  |  |
| ATR | **124** | [^3^H]mepyramine |  |  |  |
| Br-6-APT | **125** | [^3^H]mepyramine |  |  |  |
| BU-E 47 | **126** | [^3^H]mepyramine |  |  |  |
| Burimamide | **127** |  | [^125^I]iodoamino-potentidine,  [^3^H]histamine | [^3^H]RAMH ,  [^3^H]histamine | [^3^H]NAMH, [^3^H]histamine |
| Butaclamol,(+) | **128** | [^3^H]mepyramine |  |  |  |
| Cariprazine | **129** | [^3^H]mepyramine |  |  |  |
| Cetirizine | **130** | [^3^H]mepyramine |  |  |  |
| Cetirizine (+) isomer | **131** | [^3^H]mepyramine |  |  |  |
| CF3-5-APT | **132** | [^3^H]mepyramine |  |  |  |
| CF3-6-APT | **133** | [^3^H]mepyramine |  |  |  |
| Chlorpheniramine | **134** | [^3^H]mepyramine |  |  | [^3^H]mepyramine |
| Chlorpromazine | **135** | [^3^H]mepyramine | [^3^H]tiotidine |  | [^3^H]mepyramine, [^3^H]histamine |
| Cimbi-712 | **136** | [^3^H]mepyramine |  |  |  |
| ***Ligand*** | ***Ligand no.*** | ***Radioligand hH_1_R*** | ***Radioligand hH_2_R*** | ***Radioligand hH_3_R*** | ***Radioligand hH_4_R*** |
| Cimbi-717 | **137** | [^3^H]mepyramine |  |  |  |
| Cimbi-772 | **138** | [^3^H]mepyramine |  |  |  |
| Cimbi-775 | **139** | [^3^H]mepyramine |  |  |  |
| cimetidine | **140** |  | [^125^I]iodoamino-potentidine |  |  |
| Cinnarizine | **141** |  |  |  | [^3^H]mepyramine |
| Cipralisant | **142** |  |  | [^3^H]RAMH | [^3^H]RAMH, [^3^H]histamine |
| Ciproxifan | **143** | [^3^H]RAMH |  | [^3^H]RAMH ,  [^3^H]NAMH | [^3^H]histamine |
| cis-H_2_-PAT(-) | **144** | [^3^H]trans-H_2_-PAT,(-) ;  [^3^H]mepyramine |  |  |  |
| cis-H_2_-PAT(+) | **145** | [^3^H]trans-H_2_-PAT,(-) ;  [^3^H]mepyramine |  |  |  |
| cis-H_2_-PAT(+/-) | **146** | [^3^H]trans-H_2_-PAT,(-) ;  [^3^H]mepyramine |  |  |  |
| Citalopram | **147** | [^3^H]mepyramine |  |  |  |
| CL-5-APT | **148** | [^3^H]mepyramine |  |  |  |
| CL-6-APT | **149** | [^3^H]mepyramine |  |  |  |
| Clemizole | **150** |  |  |  | [^3^H]mepyramine |
| Clobenpropit | **151** | [^3^H]mepyramine | [^3^H]tiotidine | [^3^H]histamine,  [^3^H]RAMH,  [^3^H]NAMH | [^3^H]NAMH, [^3^H]JNJ 7777120, [^3^H]histamine, [^125^I]iodophen-propit |
| Clozapine | **152** | [^3^H]mepyramine  , [^3^H]histamine | [^3^H]tiotidine |  | [^3^H]mepyramine  , [^3^H]JNJ 7777120, [^3^H]histamine |
| Cocaine | **153** | [^3^H]mepyramine |  |  |  |
| Cyproheptadine | **154** | [^3^H]mepyramine |  |  | [^3^H]mepyramine |
| d-Chlorpheniramine | **155** | [^3^H]trans-H_2_-PAT,(-) ;  [^3^H]mepyramine |  |  |  |
| Desipramine | **156** | [^3^H]mepyramine |  |  |  |
| Desmethylcitalopram | **157** | [^3^H]mepyramine |  |  |  |
| Desmethyldesipramine | **158** | [^3^H]mepyramine | [^3^H]cimetidine |  |  |
| Dimaprit | **159** |  | [^3^H]histamine | [^3^H]RAMH | [^3^H]histamine |
| Dimethylamylamine | **160** | [^3^H]mepyramine |  |  |  |
| Dimethylhistaprodifen | **161** | [^3^H]mepyramine |  |  |  |
| Diphenhydramine | **162** | [^3^H]trans-H_2_-PAT,(-) ;  [^3^H]mepyramine |  |  |  |
| ***Ligand*** | ***Ligand no.*** | ***Radioligand hH_1_R*** | ***Radioligand hH_2_R*** | ***Radioligand hH_3_R*** | ***Radioligand hH_4_R*** |
| Diphenidine | **163** | [^3^H]mepyramine |  |  |  |
| Doxepin | **164** | [^3^H]trans-H_2_-PAT,(-) ;  [^3^H]mepyramine |  |  | [^3^H]mepyramine |
| Duloxetine | **165** | [^3^H]mepyramine |  |  |  |
| Ebastine | **166** | [^3^H]mepyramine |  |  |  |
| Escitalopram | **167** | [^3^H]mepyramine |  |  |  |
| Famotidine | **168** |  | [^125^I]iodoamino-potentidine |  |  |
| FECIMBI-36 | **169** | [^3^H]mepyramine | [^3^H]tiotidine |  |  |
| Fexofenadine | **170** | [^3^H]mepyramine |  |  |  |
| Flupenthixol, α | **171** | [^3^H]mepyramine |  |  |  |
| Flupenthixol, β | **172** | [^3^H]mepyramine |  |  |  |
| Fluphenazine | **173** | [^3^H]mepyramine | [^3^H]tiotidine |  |  |
| Fluspirilene | **174** | [^3^H]mepyramine |  |  |  |
| GBR-12909 | **175** | [^3^H]mepyramine |  |  |  |
| GT 2016 | **176** | [^3^H]mepyramine | [^3^H]tiotidine |  | [^3^H]histamine |
| GT 2227 | **177** |  | [^3^H]NAMH |  |  |
| GT 2331 | **178** |  |  | [^3^H]RAMH |  |
| Haldol | **179** | [^3^H]mepyramine |  |  |  |
| Haloperidol | **180** | [^3^H]mepyramine  , | [^3^H]tiotidine |  |  |
| HIS | **181** | [^3^H]mepyramine  ; functional | functional |  |  |
| histamine | **182** | [^3^H]mepyramine  , [^3^H]histamine, functional | [^3^H]histamine | [^3^H]histamine,  [^3^H]-RAHMA, [^3^H]-methylhistamine | [^3^H]NAMH, [^3^H]JNJ 7777120, [^125^I]iodophen-propit, [^3^H]mepyramine |
| Homohistamine | **183** |  |  | [^3^H]histamine | [^3^H]NAMH |
| Hydroxyzine, (R) | **184** | [^3^H]mepyramine |  |  |  |
| Imbutamine | **185** |  |  | [^3^H]histamine | [^3^H]NAMH |
| Imetit | **186** |  |  | [^3^H]RAMH ,  [^3^H]histamine, | [^3^H]NAMH, [^3^H]histamine,  [^3^H]mepyramine |
| Imipramine | **187** | [^3^H]mepyramine |  |  |  |
| Immepip | **188** |  |  | [^3^H]histamine,  [^3^H]-RAHM | [^3^H]NAMH, [^3^H]JNJ 7777120, [^3^H]histamine, [^125^I]iodophen-propit |
| Immethridine | **189** |  |  | [^3^H]histamine | [^3^H]NAMH |
| Impentamine | **190** |  |  | [^3^H]RAMH ,  [^3^H]histamine | [^3^H]NAMH |
| ***Ligand*** | ***Ligand no.*** | ***Radioligand hH_1_R*** | ***Radioligand hH_2_R*** | ***Radioligand hH_3_R*** | ***Radioligand hH_4_R*** |
| Impromidine | **191** |  | [^125^I]iodoamino-potentidine,  [^3^H]histamine | [^3^H]histamine,  [^3^H]RAMH | [^3^H]NAMH, [^3^H]histamine |
| Iodophenpropit | **192** |  |  | [^3^H]RAMH ,  [^3^H]histamine | [^3^H]NAMH, [^3^H]JNJ 7777120, [^3^H]histamine, [^125^I]iodophen-propit |
| Iodoproxyfan | **193** |  |  | [^3^H]histamine | [^3^H]NAMH |
| JNJ 7777120 | **194** |  |  | [^3^H]histamine | [^3^H]NAMH, [^3^H]JNJ 7777120, [^3^H]histamine, [^125^I]iodophen-propit |
| JNJ-5207852 | **195** |  |  | [^3^H]JNJ-5207852 |  |
| Ketanserin | **196** | [^3^H]mepyramine |  |  |  |
| l-Chlorpheniramine | **197** | [^3^H]trans-H_2_-PAT,(-) ;  [^3^H]mepyramine |  |  |  |
| Levocetirizine | **198** | [^3^H]mepyramine |  |  |  |
| Loperamide | **199** | [^3^H]mepyramine |  |  |  |
| Loratidine | **200** | [^3^H]mepyramine |  |  |  |
| Loxapine | **201** | [^3^H]mepyramine  , [^3^H]histamine | [^3^H]tiotidine |  | [^3^H]histamine |
| Mazindol | **202** | [^3^H]mepyramine |  |  |  |
| mCPP | **203** | [^3^H]mepyramine |  |  |  |
| MDL 100907 | **204** | [^3^H]mepyramine |  |  |  |
| MDL 11,939 | **205** | [^3^H]mepyramine |  |  |  |
| MDMA | **206** | [^3^H]mepyramine |  |  |  |
| Mepyramine | **207** | [^3^H]trans-H_2_-PAT,(-) ,  [^3^H]mepyramine |  |  |  |
| Methimmepip | **208** |  |  | [^3^H]histamine | [^3^H]NAMH |
| Methylhistaprodifen | **209** | [^3^H]mepyramine |  |  |  |
| Methysergide | **210** | [^3^H]mepyramine |  |  |  |
| MH.MZ | **211** |  | [^3^H]cimetidine |  |  |
| Mianserin | **212** | [^3^H]mepyramine |  |  | [^3^H]mepyramine |
| Mirtazapine | **213** | [^11^C]-R107474,  [^3^H]mepyramine |  |  |  |
| Mitragynine | **214** |  |  | [^3^H]RAMH |  |
| Mizolastine | **215** | [^3^H]mepyramine |  |  |  |
| Molindone | **216** | [^3^H]mepyramine |  |  |  |
| MRS5698 | **217** |  |  |  | [^3^H]histamine |
| MRS5979 | **218** | [^3^H]mepyramine |  |  |  |
| N-(9,10-Dihydro-9-anthracenylmethyl)-2-phenylethanamine | **219** | [^3^H]mepyramine |  |  |  |
| ***Ligand*** | ***Ligand no.*** | ***Radioligand hH_1_R*** | ***Radioligand hH_2_R*** | ***Radioligand hH_3_R*** | ***Radioligand hH_4_R*** |
| N-(9,10-Dihydro-9-anthracenylmethyl)-3-phenyl-1-propanamine | **220** | [^3^H]mepyramine |  |  |  |
| N-(9,10-Dihydro-9-anthracenylmethyl)-4-phenyl-1-butanamine | **221** | [^3^H]mepyramine |  |  |  |
| N-α-methylhistamine | **222** |  |  | [^3^H]histamine | [^3^H]NAMH |
| Nantenin | **223** |  | [^3^H]cimetidine |  |  |
| Naphyrone | **224** | [^3^H]mepyramine | [^3^H]cimetidine |  |  |
| N-Desalkylquetiapine | **225** | [^3^H]mepyramine | [^3^H]cimetidine |  | [^3^H]NMSP |
| N-desmethylclozapine | **226** | [^3^H]mepyramine | [^3^H]tiotidine |  | [^3^H]histamine |
| Nefazodone | **227** | [^3^H]mepyramine |  |  |  |
| N-methylhistamine | **228** |  |  | [^3^H]RAMH | [^3^H]histamine |
| Nortryptyline | **229** | [^3^H]mepyramine |  |  |  |
| OCH3-5-APT | **230** | [^3^H]mepyramine |  |  |  |
| OCH3-6-APT | **231** | [^3^H]mepyramine |  |  |  |
| Olanzapine | **232** | [^3^H]mepyramine  , [^3^H]mepyramine | [^3^H]tiotidine |  |  |
| ORG-5222 | **233** | [^3^H]mepyramine | [^3^H]tiotidine |  |  |
| pdspTestCompound2 | **234** | [^3^H]mepyramine |  |  |  |
| pdspTestCompound2 | **235** | [^3^H]mepyramine |  |  |  |
| pdspTestCompound3 | **236** | [^3^H]mepyramine |  |  |  |
| Perphenazine | **237** | [^3^H]mepyramine |  |  |  |
| Pheniramine | **238** |  |  |  | [^3^H]mepyramine |
| Pimozide | **239** | [^3^H]mepyramine |  |  |  |
| Pipamperone | **240** | [^3^H]mepyramine |  |  |  |
| Pramipexole | **241** |  | [^3^H]tiotidine |  |  |
| Promethazine | **242** | [^3^H]mepyramine |  |  | [^3^H]mepyramine |
| Proxyfan | **243** |  | [^3^H]histamine | [^3^H]RAMH | [^3^H]NAMH |
| Quetiapine | **244** | [^3^H]mepyramine |  |  |  |
| Raclopride | **245** | [^3^H]mepyramine |  |  |  |
| R-α-methylhistamine | **246** |  |  | [^3^H]RAMH | [^3^H]histamine |
| RAMH | **247** |  |  | [^3^H]NAMH |  |
| Ranitidine | **248** |  | [^125^I]iodoamino-potentidine |  |  |
| Rimcazole | **249** | [^3^H]mepyramine |  |  |  |
| Risperidone | **250** | [^3^H]mepyramine | [^3^H]tiotidine |  |  |
| RWAY,(-) | **251** | [^3^H]mepyramine | [^3^H]tiotidine |  |  |
| S-34324 | **252** | [^3^H]mepyramine |  |  |  |
| SCH 23390 | **253** | [^3^H]mepyramine |  |  |  |
| Sertindole | **254** | [^3^H]mepyramine |  |  |  |
| Sertraline | **255** | [^3^H]mepyramine |  |  |  |
| SKF 38393 | **256** | [^3^H]mepyramine |  |  |  |
| Sopromidine R(-) | **257** |  | [^3^H]histamine |  |  |
| Sopromidine S(+) | **258** |  | [^3^H]histamine |  |  |
| Spiperone | **259** | [^3^H]mepyramine |  |  |  |
| SR 147778 | **260** | undefined | undefined | undefined |  |
| Terfenadine | **261** | [^3^H]mepyramine |  |  |  |
| ***Ligand*** | ***Ligand no.*** | ***Radioligand hH_1_R*** | ***Radioligand hH_2_R*** | ***Radioligand hH_3_R*** | ***Radioligand hH_4_R*** |
| Terfenadine, R(+) | **262** | [^3^H]mepyramine |  |  |  |
| Thioperamide | **263** |  |  | [^3^H]JNJ 5207852, [^3^H]RAMH ,  [^3^H]histamine, [^3^H]NAMH  , | [^3^H]NAMH, [^125^I]iodophen-propit,  [^3^H]histamine,  [^3^H]JNJ 7777120, [^3^H] JNJ 5207852 |
| Thioridazine | **264** | [^3^H]mepyramine | [^3^H]tiotidine |  | [^3^H]histamine |
| Thiothixene | **265** | [^3^H]mepyramine | [^3^H]tiotidine |  |  |
| Tiotidine | **266** |  | [^125^I]iodoamino-potentidine |  |  |
| trans-H_2_-PAT(-) | **267** | [^3^H]trans-H_2_-PAT,(-) ;  [^3^H]mepyramine |  |  |  |
| trans-H_2_-PAT(+) | **268** | [^3^H]trans-H_2_-PAT,(-) ;  [^3^H]mepyramine |  |  |  |
| trans-H_2_-PAT(+/-) | **269** | [^3^H]trans-H_2_-PAT,(-) ;  [^3^H]mepyramine |  |  |  |
| Trazodone | **270** | [^3^H]mepyramine | [^3^H]tiotidine |  |  |
| Triflouperazine | **271** | [^3^H]mepyramine |  |  |  |
| Triprolidine | **272** | [^3^H]trans-H_2_-PAT,(-) ;  [^3^H]mepyramine |  |  |  |
| ucb 29992, (R) | **273** | [^3^H]mepyramine |  |  |  |
| ucb 29993, (S) | **274** | [^3^H]mepyramine |  |  |  |
| UR-AK22 | **275** | [^3^H]mepyramine | functional |  |  |
| UR-AK24 | **276** | [^3^H]mepyramine | functional |  |  |
| UR-AK26 | **277** |  | functional |  |  |
| UR-AK41 | **278** |  | functional |  |  |
| UR-AK46 | **279** |  | functional |  |  |
| UR-AK49 | **280** |  | functional |  |  |
| UR-AK51 | **281** |  | functional |  |  |
| UR-AK57 | **282** | [^3^H]mepyramine  , functional | functional |  |  |
| UR-AK59 | **283** | [^3^H]mepyramine  , functional | functional |  |  |
| UR-AK62 | **284** | [^3^H]mepyramine | functional |  |  |
| UR-AK64 | **285** | [^3^H]mepyramine |  |  |  |
| UR-AK67 | **286** |  | functional |  |  |
| UR-AK68 | **287** | functional | functional |  |  |
| VUF4614 | **288** |  |  |  | [^3^H]histamine |
| VUF4616 | **289** |  |  |  | [^3^H]histamine |
| ***Ligand*** | ***Ligand no.*** | ***Radioligand hH_1_R*** | ***Radioligand hH_2_R*** | ***Radioligand hH_3_R*** | ***Radioligand hH_4_R*** |
| VUF4617 | **290** |  | [^125^I]iodoamino-potentidine |  | [^3^H]histamine |
| VUF4681 | **291** |  |  |  | [^3^H]histamine |
| VUF4682 | **292** |  |  |  | [^3^H]histamine |
| VUF4683 | **293** |  |  |  | [^3^H]histamine |
| VUF4686 | **294** |  |  |  | [^3^H]histamine |
| VUF4687 | **295** |  |  |  | [^3^H]histamine |
| VUF4742 | **296** |  | [^125^I]iodoamino-potentidine |  | [^3^H]histamine |
| VUF4903 | **297** |  |  | [^3^H]RAMH |  |
| VUF4904 | **298** |  |  | [^3^H]RAMH |  |
| VUF5202 | **299** |  |  | [^3^H]RAMH |  |
| VUF5207 | **300** |  |  | [^3^H]RAMH |  |
| VUF5300 | **301** |  |  | [^3^H]RAMH |  |
| VUF8328 | **302** |  |  | [^3^H]RAMH ,  [^3^H]histamine | [^3^H]NAMH |
| Ziprasidone | **303** | [^3^H]mepyramine | [^3^H]tiotidine |  |  |
| Zotepine | **304** | [^3^H]mepyramine | [^3^H]tiotidine |  | [^3^H]histamine |

***Supplemental Table 3:*** *Overview of pK_d_ values for radioligands frequently used at recombinant hH_x_R (see Supplemental Table 2). Values were retrieved from the IUPHAR/BPS Guide to Pharmacology [34] and are given as the negative decadic logarithm of the equilibrium dissociation constant determined in radioligand binding assays [11]. Radioligands are further characterised by their functional behaviour for the H_x_R provided with pK_d_ values [34]. Empty fields indicate that no corresponding pK_d_ value was listed for the respective radioligand–receptor combination.*

| **Radioligand** | **Functional label** | **pK_d_** | | | |
| --- | --- | --- | --- | --- | --- |
|  |  | **hH_1_R** | **hH_2_R** | **hH_3_R** | **hH_4_R** |
| [^3^H]mepyramine | Antagonist/ inverse agonist (hH_1_R), antagonist (hH_4_R) | 8.4-9.1 |  |  | 7.5 |
| [^125^I]iodoaminopotentidine | Antagonist |  | 8.7 |  |  |
| [^3^H]tiotidine | Antagonist |  | 7.7-8.7 |  |  |
| [^3^H]RAMH | Agonist |  |  | 9.2 | 7.2 |
| [^3^H]NAMH | Agonist |  |  | 9.2 | 7.2 |
| [^3^H]histamine | Agonist |  |  | 8 | 7.4-8.4 |
| [^3^H]JNJ7777120 | Antagonist |  |  |  | 8.4 |

***Supplemental Table 4:*** *Overview about the number and share of ligands, as well as the binding affinity (pK_i_) for all four hH_x_R. The according pK_i_ values for the analysed ligands can be found in Table 1 and Supplemental Table 1.*

| **hH_x_R** | **Number of ligands** | **Share of ligands** | **pK_i_ (median)** | **pK_i_ (range)** |
| --- | --- | --- | --- | --- |
| hH₁R | 185 | 46.8% | 6.6 | 5.0-10.2 |
| hH₂R | 64 | 16.2% | 6.85 | 5.1-8.6 |
| hH₃R | 70 | 17.7% | 7.7 | 5.1-10.3 |
| hH₄R | 76 | 19.2% | 6.7 | 5.0-8.4 |
| Sum/median | 395 | 18.6% | 6.775 | 5.0-10.3 |

***Supplemental Table 5:*** *Comparison of listed ligands for hH_x_R between the PDSP K_i_ database and the IUPHAR/BPS Guide to Pharmacology.*

| **All histamine receptors with active binding affinities listed as a target in IUPHAR (n=48)** | **Part of the histamine receptors with active binding affinity listed in IUPHAR (n=31)** | **Ligand listed, but histamine receptor omitted as target (n=40)** | **Ligand is not listed in IUPHAR (n=183)** |
| --- | --- | --- | --- |
| Arpromidine | 2-methylhistamine | CF3-5-APT | (-)-(2RS,3aRS,12bSR)-2-N,N-Dimethylaminomethyl-11-fluoro-2,3,3a,12b-tetrahydrodibenzo[b,f]furo[2,3-d]tiepin |
| Ciproxifan | Histamine | CF3-6-APT | (-)-Cimbi-772 |
| Impromidine | Clozapine | (-)-SYA 09 | (-)-Cimbi-775 |
| Clobenpropit | Dimaprit | 7-OH-DPAT | (-)-NANM |
| ABT-239 | 4-methylhistamine | Amoxapine | (+)-(2RS,3aRS,12bSR)-2-N,N-Dimethylaminomethyl-11-fluoro-2,3,3a,12b-tetrahydrodibenzo[b,f]furo[2,3-d]tiepin |
| Burimamide | Amitriptyline | Atomoxetine | (+)-7,8-Dimethoxy-3-[4-(2-methyl-3-phenyl-2(E)-propen-1-yl)piperazin-1-ylmethyl]-3a,4-dihydro-^3^H-[1]benzopyrano[4,3-c]isoxazole |
| A-317920 | Chlorpheniramine | Butaclamol, (+) | (+)-7,8-Dimethoxy-3-[4-(3-methyl-3-phenyl-2(E)-propen-1-yl)piperazin-1-ylmethyl]-3a,4-dihydro-^3^H-[1]benzopyrano[4,3-c]isoxazole |
| A-349821 | Cipralisant | Cariprazine | (+)-7,8-Dimethoxy-3-[4-(3-phenyl-2(E)-propen-1-yl)piperazin-1-ylmethyl]-3a,4-dihydro-^3^H-[1]benzopyrano[4,3-c]isoxazole |
| Imetit | Cyproheptadine | Cinnarizine | (+)-7,8-Dimethoxy-3-[4-(naphthalen-2-ylmethyl)piperazin-1-ylmethyl]-3a,4-dihydro-^3^H-[1]benzopyrano[4,3-c]isoxazole |
| Immepip | Doxepin | cis-H_2_-PAT(+/-) | Cimbi-772 (+) |
| Iodophenpropit | Fluphenazine | Citalopram | Cimbi-775 (+) |
| N-methylhistamine | Haloperidol | CL-6-APT | SYA 09 (+) |
| R-α-methylhistamine | Imbutamine | Clemizole | BMY-1402 (+/-) |
| Thioperamide | Immethridine | Cocaine | (1H-Indol-2-yl)-(4-methyl-piperazin-1-yl)-methanone |
| A-304121 | Impentamine | Desipramine | (1H-Indol-2-yl)-(4-phenethyl-piperazin-1-yl)-methanone |
| amthamine | Iodoproxyfan | Duloxetine | (1H-Indol-2-yl)-(piperazin-1-yl)-methanone |
| Aripiprazole | JNJ 7777120 | Flupenthixol, α | (2RS,3aRS,12bSR)-2-N,N-Dimethylaminomethyl-11-bromo-2,3,3a,12b-tetrahydrodibenzo[b,f]furo[2,3-d]oxepin |
| Astemizole | N-α-methylhistamine | Flupenthixol, β | (2RS,3aRS,12bSR)-2-N,N-Dimethylaminomethyl-11-chloro-2,3,3a,12b-tetrahydrodibenzo[b,f]furo[2,3-d]oxepin |
| BU-E 47 | Olanzapine | GBR-12909 (Vanoxerine) | (2RS,3aRS,12bSR)-2-N,N-Dimethylaminomethyl-11-fluoro-2,3,3a,12b-tetrahydrodibenzo[b,f]furo[2,3-d]oxepin |
| Cetirizine | ORG-5222 (Asenapine) | Imipramine | (2RS,3aRS,12bSR)-2-N,N-Dimethylaminomethyl-11-fluoro-2,3,3a,12b-tetrahydrodibenzo[b,f]furo[2,3-d]tiepin |
| Cimetidine | Promethazine | Ketanserin | (2SR,3aRS,12bSR)-2-N,N-Dimethylaminomethyl-11-chloro-2,3,3a,12b-tetrahydrodibenzo[b,f]furo[2,3-d]oxepin |
| cis-H_2_-PAT(+) | Risperidone | Loperamide | (2SR,3aRS,12bSR)-2-N,N-Dimethylaminomethyl-11-fluoro-2,3,3a,12b-tetrahydrodibenzo[b,f]furo[2,3-d]oxepin |
| ***All histamine receptors with active binding affinities listed as a target in IUPHAR (n=48)*** | ***Part of the histamine receptors with active binding affinity listed in IUPHAR (n=31)*** | ***Ligand listed, but histamine receptor omitted as target (n=40)*** | ***Ligand is not listed in IUPHAR (n=183)*** |
| Dimethylhistaprodifen | Thiothixene | Mazindole | (2SR,3aRS,12bSR)-2-N,N-Dimethylaminomethyl-11-fluoro-2,3,3a,12b-tetrahydrodibenzo[b,f]furo[2,3-d]tiepin |
| Diphenhydramine | Ziprasidone | MDMA | (3,5-Dinitropyridin-2-yl){2-[2-(2-(R)-methylpyrrolidin-1-yl)ethyl]benzofuran-5-ylmethyl}amine |
| Famotidine | 9-OH-risperidone | Methysergine | (3S,4S,6aS,9S,10S,12aS)-4,10-bis(4-(3-(dimethylamino)prop-1-ynyl)phenyl)-3,9-dimethyloctahydrodipyrido[1,2-a:1',2'-d]pyrazine-6,12(6aH,12aH)-dione |
| Fexofenadine | Chlorpromazine | Mianserin | (3S4S6aS9S10S12aS)-4-(2-bromophenyl)-10-(4-(3-(dimethylamino)prop-1-ynyl)phenyl)-39-dimethyloctahydrodipyrido[12-a:1'2'-d]pyrazine-612(6aH12aH)-dione |
| Fluspirilene | Loxapine | Mirtazapine | (3S4S6aS9S10S12aS)-410-bis(4-(3-(dimethylamino)prop-1-ynyl)phenyl)-39-dimethyloctahydrodipyrido[12-a:1'2'-d]pyrazine-612(6aH12aH)-dione |
| Haldol | Proxyfan | Mitragynine | (4-Bromo-1H-indol-2-yl)-(4-methyl-piperazin-1-yl)-methanone |
| JNJ-5207852 | SR 147778 | MRS5698 | (4-Ethyl-piperazin-1-yl)-(1H-indol-2-yl)-methanone |
| Levocetirizine | Thioridazine | N-desalkylquetiapine | (5,7- Dichloro-1H-indol-2-yl)-(4-methyl-piperazin-1-yl)-methanone |
| Loratidine | A-331440 | N-desmethylclozapine | (5,7-Difluoro-1H-indol-2-yl)-(4-methyl-piperazin-1-yl)-methanone |
| Mepyramine |  | Nefazodone | (5,7-Dimethyl-1H-indol-2-yl)-(4-methyl-piperazin-1-yl)-methanone |
| Methylhistaprodifen |  | Pheniramine | (5-Amino-1H-indol-2-yl)-(4-methyl-piperazin-1-yl)-methanone |
| Mizolastine |  | Pramipexole | (5-Bromo-1H-indol-2-yl)-(4-methyl-piperazin-1-yl)-methanone |
| Molindone |  | Raclopride | (5-Bromopyrimidin-2-yl){2-[2-(2-(R)-methylpyrrolidin-1-yl)ethyl]benzofuran-5-ylmethyl}amine |
| Perphenazine |  | Sertraline | (5-Chloro-1H-indol-2-yl)-(4-methyl-piperazin-1-yl)-methanone |
| Pimozide |  | SKF 38393 | (5-Ethylpyrimidin-2-yl){2-[2-(2-(R)-methylpyrrolidin-1-yl)ethyl]benzofuran-5-yl}amine |
| Pipamerone |  | Spiperone | (5-Ethylpyrimidin-2-yl){2-[2-(2-(R)-methylpyrrolidin-1-yl)ethyl]benzofuran-5-ylmethyl}amine |
| Quetiapine |  | Trazodone | (5-Fluoro-1H-indol-2-yl)-(4-methyl-piperazin-1-yl)-methanone |
| Ranitidine |  | Zotepine | (5-Hydroxy-1H-indol-2-yl)-(4-methyl-piperazin-1-yl)-methanone |
| Sertindole |  |  | (5-Methoxy-1H-indol-2-yl)-(4-methyl-piperazin-1-yl)-methanone |
| Terfenadine |  |  | (5-Methyl-1H-indol-2-yl)-(4-methyl-piperazin-1-yl)-methanone |
| Tiotidine |  |  | (6-Bromo-1H-indol-2-yl)-(4-methyl-piperazin-1-yl)-methanone |
| trans-H_2_-PAT(-) |  |  | (6-Chloropyridazin-3-yl){2-[2-(2-methylpyrrolidin-1-yl)ethyl]benzofuran-5-ylmethyl}amine |
| trans-H_2_-PAT(+) |  |  | (7-Amino-1H-indol-2-yl)-(4-methyl-piperazin-1-yl)-methanone |
| trans-H_2_-PAT(+/-) |  |  | (7-Bromo-1H-indol-2-yl)-(4-methyl-piperazin-1-yl)-methanone |
| Trifluoperazine |  |  | (7-Chloro-1H-indol-2-yl)-(4-methyl-piperazin-1-yl)-methanone |
| Triprolidine |  |  | (7-Methyl-1H-indol-2-yl)-(4-methyl-piperazin-1-yl)-methanone |
|  |  |  | ***Ligand is not listed in IUPHAR (n=183)*** |
|  |  |  | R-chlopheniramine |
|  |  |  | [5-Fluoro-2-methyl-1-(4-methanesulfonyl-benzenesulfonyl)-1H-indol-3-yl]-acetic acid |
|  |  |  | {2-[2-(2-(R)-Methylpyrrolidin-1-yl)ethyl]benzofuran-5-yl}(2-nitrophenyl)amine |
|  |  |  | {2-[2-(2-(R)-Methylpyrrolidin-1-yl)ethyl]benzofuran-5-yl}(3-nitropyridin-2-yl)amine |
|  |  |  | {2-[2-(2-(R)-Methylpyrrolidin-1-yl)ethyl]benzofuran-5-yl}(5-nitropyridin-2-yl)amine |
|  |  |  | {2-[2-(2-(R)-Methylpyrrolidin-1-yl)ethyl]benzofuran-5-yl}(5-trifluoromethylpyridin-2-yl)amine |
|  |  |  | {2-[2-(2-(R)-Methylpyrrolidin-1-yl)ethyl]benzofuran-5-yl}pyrazin-2-ylamine |
|  |  |  | {2-[2-(2-(R)-Methylpyrrolidin-1-yl)ethyl]benzofuran-5-yl}pyrimidin-2-ylamine |
|  |  |  | {2-[2-(2-(R)-Methylpyrrolidin-1-yl)ethyl]benzofuran-5-yl}pyrimidin-5-ylamine |
|  |  |  | {2-[2-(2-(R)-Methylpyrrolidin-1-yl)ethyl]benzofuran-5-ylmethyl}(2-nitrophenyl)amine |
|  |  |  | {2-[2-(2-(R)-Methylpyrrolidin-1-yl)ethyl]benzofuran-5-ylmethyl}(3-nitropyridin-2-yl)amine |
|  |  |  | {2-[2-(2-(R)-Methylpyrrolidin-1-yl)ethyl]benzofuran-5-ylmethyl}(5-nitropyridin-2-yl)amine |
|  |  |  | {2-[2-(2-(R)-Methylpyrrolidin-1-yl)ethyl]benzofuran-5-ylmethyl}(5-nitrothiazol-2-yl)amine |
|  |  |  | {2-[2-(2-(R)-Methylpyrrolidin-1-yl)ethyl]benzofuran-5-ylmethyl}pyrazin-2-ylamine |
|  |  |  | {2-[2-(2-(R)-Methylpyrrolidin-1-yl)ethyl]benzofuran-5-ylmethyl}pyrimidin-2-ylamine |
|  |  |  | {2-[2-(2-(R)-Methylpyrrolidin-1-yl)ethyl]benzofuran-5-ylmethyl}pyrimidin-5-ylamine |
|  |  |  | {2-[2-(2-Methylpyrrolidin-1-yl)ethyl]benzofuran-5-ylmethyl}(4-nitrophenyl)amine |
|  |  |  | (+/-)-(1H-Indol-2-yl)-(3-methyl-piperazin-1-yl)-methanone |
|  |  |  | 1-(3-Methoxy-7,12-dihydro-12-tetraphenyl)methanamine |
|  |  |  | 11-(Piperazin-1-yl)-5H-dibenzo[b,e][1,4]diazepine |
|  |  |  | 2-({2-[2-(2-(R)-Methylpyrrolidin-1-yl)ethyl]benzofuran-5-ylmethyl}amino)nicotinonitrile |
|  |  |  | 2-(2-Thiazolyl)ethanamine |
|  |  |  | 2-(3-Chlorophenyl)histamine |
|  |  |  | 2-(3-Iodophenyl)histamine |
|  |  |  | 2-(Dimethylamino)tetralin |
|  |  |  | 2,2,2-Trifluoro-1-[4-({2-[2-(2-methylpyrrolidin-1-yl)ethyl]benzofuran-5-ylmethyl}amino)phenyl]ethanone |
|  |  |  | 2-{4-[4-(3-Methoxyphenyl)-1-piperazinyl]butyl}-4-methyl-1,2,4-triazine-3,5(2H,4H)-dione |
|  |  |  | 2-{4-[4-(7-Methoxy-naphthalen-1-yl)piperazin-1-yl]-butyl}-4-methyl-2H-[1,2,4]triazine-3,5-dione |
|  |  |  | ***Ligand is not listed in IUPHAR (n=183)*** |
|  |  |  | 2-methoxy-diphenidine |
|  |  |  | 3-({2-[2-(2-(R)-Methylpyrrolidin-1-yl)ethyl]benzofuran-5-ylmethyl}amino)benzonitrile |
|  |  |  | 3-({2-[2-(2-(R)-Methylpyrrolidin-1-yl)ethyl]benzofuran-5-ylmethyl}amino)pyrazine-2-carbonitrile |
|  |  |  | 3-(Dimethylamino)fluoranthene |
|  |  |  | 3-{2-[2-(2-(R)-Methylpyrrolidin-1-yl)ethyl]benzofuran-5-ylamino}benzonitrile |
|  |  |  | 3-Aminofluoranthene |
|  |  |  | 3-methoxy-diphenidine |
|  |  |  | 4-({2-[2-(2-(R)-Methylpyrrolidin-1-yl)ethyl]benzofuran-5-ylmethyl}amino)pyridine-2,6-dicarbonitrile |
|  |  |  | 4-(3-piperidin-1-yl-propoxy)-benzonitrile |
|  |  |  | 4-(8-Chloro-5H-dibenzo[b,e][1,4]diazepin-11-yl)-1-ethylpiperazine N-oxide |
|  |  |  | 4-(8-Chloro-5H-dibenzo[b,e][1,4]diazepin-11-yl)-1-isopropylpiperazine N-oxide |
|  |  |  | 4-[3-(4-Chlorophenyl)-3-hydroxy-8-azabicyclo[3.2.1]oct-8-yl]-1-(4-fluorophenyl)-1-butanone |
|  |  |  | 4-[5-trans-hydroxy-5-cis-(4′-chlorophenyl)-2-azabicyclo[3.2.1]octanyl]-4″-fluorobutyrophenone |
|  |  |  | 4-{2-[2-(2-(R)-Methylpyrrolidin-1-yl)ethyl]benzofuran-5-ylamino}benzonitrile |
|  |  |  | 4-methoxy-diphenidine |
|  |  |  | 5-APT |
|  |  |  | 6-({2-[2-(2-(R)-Methylpyrrolidin-1-yl)ethyl]benzofuran-5-ylmethyl}amino)nicotinonitrile |
|  |  |  | 6-APT |
|  |  |  | 6-Methyl-2-{2-[2-(2-(R)-methylpyrrolidin-1-yl)ethyl]benzofuran-5-ylamino}nicotinonitrile |
|  |  |  | 8-Chloro-11-(4-ethylpiperazin-1-yl)-5H-dibenzo[b,e][1,4]diazepine |
|  |  |  | 8-Chloro-11-(4-isopropylpiperazin-1-yl)-5H-dibenzo[b,e][1,4]diazepine |
|  |  |  | 8-Chloro-11-[4-(1,1-dideutrioethyl)piperazin-1-yl]-5H-dibenzo[b,e][1,4]diazepine |
|  |  |  | 8R-lisuride |
|  |  |  | A-80426 |
|  |  |  | R-α-methylhistamine |
|  |  |  | S-α-methylhistamine |
|  |  |  | Aminopotentidine |
|  |  |  | Amselamine |
|  |  |  | ***Ligand is not listed in IUPHAR (n=183)*** |
|  |  |  | ATR |
|  |  |  | Br-6-APT |
|  |  |  | Cetirizine (+) isomer |
|  |  |  | Cimbi-712 |
|  |  |  | Cimbi-717 |
|  |  |  | Cimbi-772 |
|  |  |  | Cimbi-775 |
|  |  |  | cis-H_2_-PAT(-) |
|  |  |  | CL-5-APT |
|  |  |  | D-chlorpheniramine |
|  |  |  | Desmethylcitalopram |
|  |  |  | Desmethyldesipramine |
|  |  |  | Dimethylamylamine |
|  |  |  | Diphenidine |
|  |  |  | Ebastine |
|  |  |  | Escitalopram |
|  |  |  | FECIMBI-36 |
|  |  |  | GT 2016 |
|  |  |  | GT 2227 |
|  |  |  | GT 2331 |
|  |  |  | HIS |
|  |  |  | Homohistamine |
|  |  |  | Hydroxyzine, (R) |
|  |  |  | l-Chlorpheniramine |
|  |  |  | mCPP |
|  |  |  | MDL 100907 |
|  |  |  | MDL 11,939 |
|  |  |  | Methimmepip |
|  |  |  | MH.MZ |
|  |  |  | ***Ligand is not listed in IUPHAR (n=183)*** |
|  |  |  | MRS5979 |
|  |  |  | N-(9,10-Dihydro-9-anthracenylmethyl)-2-phenylethanamine |
|  |  |  | N-(9,10-Dihydro-9-anthracenylmethyl)-3-phenyl-1-propanamine |
|  |  |  | N-(9,10-Dihydro-9-anthracenylmethyl)-4-phenyl-1-butanamine |
|  |  |  | Nantenin |
|  |  |  | Naphyrone |
|  |  |  | Nortryptyline |
|  |  |  | OCH3-5-APT |
|  |  |  | OCH3-6-APT |
|  |  |  | pdspTestCompound2 |
|  |  |  | pdspTestCompound2 |
|  |  |  | pdspTestCompound3 |
|  |  |  | RAMH (R-α-methylhistamine) |
|  |  |  | Rimcazole |
|  |  |  | RWAY,(-) |
|  |  |  | S-34324 |
|  |  |  | SCH 23390 |
|  |  |  | Sopromidine R(-) |
|  |  |  | Sopromidine S(+) |
|  |  |  | Terfenadine, R(+) |
|  |  |  | ucb 29992, (R) |
|  |  |  | ucb 29993, (S) |
|  |  |  | UR-AK22 |
|  |  |  | UR-AK24 |
|  |  |  | UR-AK26 |
|  |  |  | UR-AK41 |
|  |  |  | UR-AK46 |
|  |  |  | UR-AK49 |
|  |  |  | UR-AK51 |
|  |  |  | ***Ligand is not listed in IUPHAR (n=183)*** |
|  |  |  | UR-AK57 |
|  |  |  | UR-AK59 |
|  |  |  | UR-AK62 |
|  |  |  | UR-AK64 |
|  |  |  | UR-AK67 |
|  |  |  | UR-AK68 |
|  |  |  | VUF4614 |
|  |  |  | VUF4616 |
|  |  |  | VUF4617 |
|  |  |  | VUF4681 |
|  |  |  | VUF4682 |
|  |  |  | VUF4683 |
|  |  |  | VUF4686 |
|  |  |  | VUF4687 |
|  |  |  | VUF4742 |
|  |  |  | VUF4903 |
|  |  |  | VUF4904 |
|  |  |  | VUF5202 |
|  |  |  | VUF5207 |
|  |  |  | VUF5300 |
|  |  |  | VUF8328 |

***Supplemental Table 6:*** *Examination of primary literature regarding binding affinities of selected ligands in the PDSP K_i_ database and the IUPHAR/BPS Guide to Pharmacology. For each reference, the number of binding affinities per hH_x_R is provided. References that are frequently cited in the IUPHAR/BPS Guide to Pharmacology are highlighted in bold. References are color-coded according to whether they are cited in both databases (green), only present in the PDSP K_i_ database (brown), or only included in the IUPHAR/BPS Guide to Pharmacology (blue). References are sorted by the hH_1_R of the most frequent listings for the IUPHAR/BPS Guide to Pharmacology.*

| **Reference** | **Reference no.** | **hH_1_R**  **PDSP K_i_** | **hH_1_R UPHAR/BPS** | **h**H_2_**R**  **PDSP K_i_** | **h**H_2_**R IUPHAR/BPS** | **hH_3_R**  **PDSP K_i_** | **hH_3_R IUPHAR/BPS** | **hH_4_R**  **PDSP K_i_** | **hH_4_R IUPHAR/BPS** |
| --- | --- | --- | --- | --- | --- | --- | --- | --- | --- |
| **Kroeze et al. 2003** | [21] | 17 | 16 |  |  |  |  |  |  |
| **Booth et al., 2002** | [10] | 26 | 10 |  |  |  |  |  |  |
| Schotte et al., 1996 | [46] | 13 | 10 |  |  |  |  |  |  |
| Seifert et al., 2003 | [44] | 10 | 5 |  |  |  |  |  |  |
| **Moguilevsky et al., 1994** | [91] | 6 | 4 |  |  |  |  |  |  |
| **Xie et al., 2006** | [43] | 22 | 3 | 14 | 2 |  |  |  |  |
| Ratnala et al., 2004 | [109] |  | 3 |  |  |  |  |  |  |
| Esbenshade et al., 2004 | [93] | 5 | 2 | 2 |  | 12 | 5 | 3 | 3 |
| Esbenshade et al., 2003 | [91] | 2 | 2 | 1 | 1 | 10 | 5 | 3 | 4 |
| Ghoneim et al., 2006 | [54] | 40 | 2 |  |  |  |  |  |  |
| Aslanian et al., 2009 | [110] |  | 2 |  |  |  |  |  |  |
| Panula et al., 2015 | [1] |  | 1 |  | 1 |  | 1 |  | 1 |
| Esbenshade et al., 2005 | [69] |  | 1 |  | 1 |  | 1 |  |  |
| Coon et al., 2009 | [111] |  | 1 |  |  |  |  |  |  |
| Janssens et al., 2005 | [112] |  | 1 |  |  |  |  |  |  |
| Pearlstein et al., 2003 | [113] |  | 1 |  |  |  |  |  |  |
| Procopiou et al., 2011 | [114] |  | 1 |  |  |  |  |  |  |
| **Lim et al., 2005** | [29] |  |  | 21 |  | 19 |  | 54 | 9 |
| PDSP Certified Data | [96] | 24 |  | 17 |  | 1 |  | 7 |  |
| Cusack et al., 1994 | [52] |  |  | 13 |  |  |  |  |  |
| Chen et al., 2015 | [76] |  |  | 7 |  |  |  |  |  |
| Dandapani et al., 2006 | [86] |  |  | 2 |  |  |  | 1 |  |
| Hancock, et al., 2004 | [78] | 3 |  | 1 |  | 5 | 1 | 4 |  |
| Deupree et. al., 2007 | [77] | 7 |  | 1 |  |  |  |  |  |
| Jensen et al., 2008 | [45] | 2 |  | 1 |  |  |  | 1 |  |
| Iversen et al., 2012 | [79] | 2 |  | 1 |  |  |  |  |  |
| Rinaldi-Carmona et al., 2004 | [57] | 1 |  | 1 |  | 1 |  |  |  |
| Armer et al., 2005 | [75] | 1 |  | 1 |  | 1 |  |  |  |
| Kumar JS, et al., 2006 | [97] | 1 |  | 1 |  |  |  |  |  |
| Citrome, 2013 | [51] |  |  | 1 |  |  |  |  |  |
| Prabhakaran et al., 2006 | [82] |  |  | 1 |  |  |  |  |  |
| Yates et al., 1999 | [88] |  |  | 1 |  | 1 |  |  |  |
| Herth et. al., 2009 | [89] |  |  | 1 |  |  |  |  |  |
| LeGendre et. al., 2009 | [90] |  |  | 1 |  |  |  |  |  |
| Prabhakaran et. al., 2015 | [98] |  |  | 1 |  |  |  |  |  |
| Alewijnse et al., 1998 | [125] |  |  |  | 1 |  |  |  |  |
| Beukers et al., 1997 | [126] |  |  |  | 1 |  |  |  |  |
| Cappelli et al., 2013 | [127] |  |  |  | 1 |  |  |  |  |
| Kraus et al., 2009 | [128] |  |  |  | 1 |  |  |  |  |
| Leurs et al., 1994 | [129] |  |  |  | 1 |  |  |  |  |
| unpublished results  (no reference available) |  |  |  |  | 1 |  |  |  |  |
| Wulff et al., 2002 | [121] |  |  |  |  |  | 10 |  |  |
| **Liu et al., 2001** | [70] | 17 |  |  |  | 11 | 9 | 11 | 11 |
| Cogé et al., 2001 | [122] |  |  |  |  |  | 7 |  |  |
| Wieland et al., 2001 | [6] |  |  |  |  | 13 | 6 |  |  |
| Lovenberg et al., 2000 | [95] |  |  |  |  | 7 | 6 |  |  |
| Kitbunnadaj et al., 2003 | [117] |  |  |  |  |  | 5 |  | 1 |
| Ligneau et al., 2000 | [123] |  |  |  |  |  | 5 |  |  |
| Apodaca et al., 2003 | [124] |  |  |  |  |  | 1 |  |  |
| **Zhu et al., 2001** | [94] |  |  |  |  | 2 |  | 4 | 8 |
| Morse et al., 2001 | [115] |  |  |  |  |  |  |  | 7 |
| Liu et al., 2001 | [42] |  |  |  |  |  |  |  | 7 |
| Gbahou et al., 2006 | [116] |  |  |  |  |  |  |  | 4 |
| Smits et al., 2006 | [118] |  |  |  |  |  |  |  | 1 |
| Terzioglu et al., 2004 | [119] |  |  |  |  |  |  |  | 1 |
| Thurmond et al., 2004 | [120] |  |  |  |  |  |  |  | 1 |
| Chang, R et. al, 1978  (reference not found) |  | 28 |  |  |  |  |  |  |  |
| Kanba and Richelson, 1984 | [99] | 24 |  |  |  |  |  |  |  |
| Richelson, 1988 | [100] | 15 |  |  |  |  |  |  |  |
| Richelson and Nelson, 1984 | [61] | 15 |  |  |  |  |  |  |  |
| Fernández et al., 2005 | [69] | 15 |  |  |  |  |  |  |  |
| Richelson and Souder, 2000 | [101] | 14 |  |  |  |  |  |  |  |
| Gillard et al., 2002 | [68] | 11 |  |  |  |  |  |  |  |
| Moguilevsky et al., 1995 | [102] | 8 |  |  |  |  |  |  |  |
| Herth et. al., 2015 | [58] | 8 |  |  |  |  |  |  |  |
| Maheux et al., 2005 | [59] | 8 |  |  |  |  |  |  |  |
| Roth and Kroeze, 2006 | [62] | 8 |  |  |  |  |  |  |  |
| Schmidt et al., 2001 | [103] | 6 |  |  |  |  |  |  |  |
| Ablordeppey S, et al., 2006 | [48] | 6 |  |  |  |  |  |  |  |
| Andrés et al., 2005 | [74] | 6 |  |  |  |  |  |  |  |
| Arias-Montano and Young, 1993 | [104] | 4 |  |  |  |  |  |  |  |
| Wallach et al., 2016 | [85] | 4 |  |  |  |  |  |  |  |
| Shah et al., 2010 | [87] | 4 |  |  |  |  |  |  |  |
| Gregori-Puigjane et al., 2012 | [105] | 1 |  |  |  | 1 |  |  |  |
| Shapiro et.al., 2003 | [106] | 1 |  |  |  |  |  |  |  |
| Bymaster et. al., 2001 | [107] | 1 |  |  |  |  |  |  |  |
| Bymaster et. al., 2002 | [50] | 1 |  |  |  |  |  |  |  |
| Van der Mey et al., 2006 | [66] | 1 |  |  |  |  |  |  |  |
| Setola et al., 2003 | [83] | 1 |  |  |  |  |  |  |  |
| Tosh et al., 2014 | [84] | 1 |  |  |  |  |  | 1 |  |
| Barbier et al., 2004 | [108] |  |  |  |  | 2 |  | 1 |  |
| Nguyen et al., 2001 | [55] |  |  |  |  |  |  | 13 |  |
| Krueger et al. 2005 | [64] |  |  |  |  | 13 |  |  |  |
| Sun et al., 2005 | [65] |  |  |  |  | 30 |  |  |  |
| Jablonowski et al., 2003 | [71] |  |  |  |  | 5 |  | 25 |  |

***Supplemental Table 7****: Comparison of database completeness by a literature search for representative ligands in PubMed (last accessed April 15 2026, search term “[ligand] binding affinity H[x]”). Included are literature-derived binding affinities (pK_i_) and comparison with database coverage. Examined ligands include loratadine (****200****) for hH_1_R, ranitidine (****248****) for hH_2_R, thioperamide (****263****) for hH_3_R, and JNJ 7777120 (****194****) for hH_4_R.*

*Only studies reporting binding affinities in human cloned receptor systems were included, while reviews and non-human species (including “undefined”) were excluded. For each publication, listing in the IUPHAR/BPS Guide to Pharmacology and the PDSP K_i_ database is reported. If binding affinities were initially reported in K_i_, they were transformed to pK_i_ values. Publications that are included in both databases are green-colored, literature that is only found in one of the two databases are yellow-colored, and publications that are missing in both databases are orange-colored.*

| **Reference** | **Reference no.** | **IUPHAR/BPS listing** | **PDSP K_i_ listing** | **Ligand** | **hH_x_R** | **pK_i_** | **notes** |
| --- | --- | --- | --- | --- | --- | --- | --- |
| Bakker et al., 2001 | [130] | no | no | loratadine/ loratidine | hH_1_R | 6.8 +/-0.1 |  |
| Lim et al., 2005 | [29] | no | yes | loratadine/ loratidine | hH_1_R | 6.8 | refers to Janssens et al., 2005 |
| Janssens et al., 2005 | [112] | yes | no | loratadine/ loratidine | hH_1_R | 7.4 |  |
| Leurs et al., 1994 | [129] | yes | no | ranitidine | hH_2_R | 7.1 |  |
| Lim et al., 2005 | [29] | no | yes | ranitidine | hH_2_R | 7.1 | refers to Leurs et al., 1994 |
| Gespach et al., 1982 | [23] | no | no | ranitidine | hH_2_R | 6.8 |  |
| Wieland et al., 2001 | [6] | yes | yes | thioperamide | hH_3_R | 7.2 |  |
| Esbenshade et al., 2003 | [91] | yes | yes | thioperamide | hH_3_R | 7.1 |  |
| Lovenberg et al., 2000 | [95] | yes | yes | thioperamide | hH_3_R | 7.2 |  |
| Bertoni et al., 2007 | [131] | no | no | thioperamide | hH_3_R | 7.2 |  |
| Reiner and Stark, 2019 | [132] | no | no | thioperamide | hH_3_R | 5.32 +/- 0.09 | functional assay instead of radioligand binding assay |
| Schnell et al., 2010 | [133] | no | no | thioperamide | hH_3_R | 7.3 4+/- 0.04 |  |
| Barbier et al., 2004 | [108] | no | yes | thioperamide | hH_3_R | 7.4 |  |
| Wulff et al., 2002 | [121] | yes | no | thioperamide | hH_3_R | 7.25 +/- 0.12 |  |
| Cogé et al., 2001 | [122] | yes | no | thioperamide | hH_3_R | 7.7 +/- 0.07 |  |
| Ligneau et al., 2000 | [123] | yes | no | thioperamide | hH_3_R | 7.2 +/- 0.09 |  |
| Liu et al., 2001 | [70] | no | yes | thioperamide | hH_3_R | 7.6 |  |
| Lim et al., 2005 | [29] | no | yes | thioperamide | hH_3_R | 7.3 |  |
| Jablonowski et al., 2003 | [71] | no | yes | thioperamide | hH_3_R | 7.6 |  |
| Esbenshade et al., 2004 | [93] | yes | no | thioperamide | hH_3_R | 7.14 +/- 0.06 |  |
| Lim et al., 2005 | [29] | yes | yes | JNJ 7777120 | hH_4_R | 7.8 +/- 0.1 |  |
| Lim et al., 2010 | [134] | no | no | JNJ 7777120 | hH_4_R | 8.3 +/- 0.1 |  |
| Schneider et al., 2010 | [135] | no | no | JNJ 7777120 | hH_4_R | 7.73 +/- 0.04 |  |
| Schnell et al., 2011 | [136] | no | no | JNJ 7777120 | hH_4_R | 7.54 +/- 0.13 |  |
| Wagner et al., 2011 | [137] | no | no | JNJ 7777120 | hH_4_R | 7.73 +/- 0.04 |  |
| Wagner et al., 2013 | [138] | no | no | JNJ 7777120 | hH_4_R | 7.45 +/- 0.1 |  |
| Smits et al., 2006 | [118] | yes | no | JNJ 7777120 | hH_4_R | 7.8 +/- 0.1 |  |
| Thurmond et al., 2004 | [120] | yes | no | JNJ 7777120 | hH_4_R | 8.39 +/- 0.03 |  |

***Table S8:*** *Comparison of the binding affinities (pK_i_) presented in the PDSP K_i_ database and the IUPHAR/BPS Guide to Pharmacology for hH_x_R.*

|  |  | **PDSP database: binding affinities (pK_i_)** | | | | **IUPHAR/BPS Guide to Pharmacology: binding affinities (pK_i_)** | | | | **Comparison pK_i_ values PDSP database vs. IUPHAR/BPS Guide to Pharmacology: deviation** | | | |
| --- | --- | --- | --- | --- | --- | --- | --- | --- | --- | --- | --- | --- | --- |
| **Ligand name** | **Ligand no.** | **hH₁R** | **hH₂R** | **hH₃R** | **hH₄R** | **hH₁R** | **hH₂R** | **hH₃R** | **hH₄R** | **hH₁R variation** | **hH₂R variation** | **hH₃R variation** | **hH₄R variation** |
| Clobenpropit | **153** | 5.6 | 5.2 | 7.2 | 7.9 | 5.6 | 5.2 | 8.9 | 7.85 | 0.0 | 0.0 | 1.7 | 0.0 |
| Impromidine | **193** |  | 6.6 | 8.3 | 7.7 | 5.2 | 7.2 | 7.2 | 7.75 |  | 0.6 | 1.1 | 0.0 |
| ABT-239 | **112** | 5.8 | 5.2 | 9.3 |  | 5.8 | 5.2 | 9.4 |  | 0.0 | 0.0 | 0.1 |  |
| Burimamide | **127** |  | 5.7 | 8.3 | 7.0 |  | 5.65 | 7.1 | 7.05 |  | 0.0 | 1.2 | 0.1 |
| Ciproxifan | **146** | 5.7 |  | 7.2 | 5.7 | <5.0 | <5.0 | 7 | 5.7 |  |  | 0.2 | 0.0 |
| Histamine | **182** | 5.9 | 7.8 | 8.3 | 7.8 | 5.3 |  | 8.05 | 8.05 | 0.6 |  | 0.2 | 0.3 |
| A-317920 | **108** | 5.4 |  | 7.0 |  | 5.4 |  | 7 |  | 0.0 |  | 0.0 |  |
| A-349821 | **110** | 5.6 |  | 9.4 |  | 5.6 |  | 9.4 |  | 0.0 |  | 0.0 |  |
| Arpromidine | **121** | 6.5 |  |  |  | 6.5 | 7.15 |  |  | 0.0 |  |  |  |
| Clozapine | **154** | 8.8 | 6.8 |  | 6.3 | 9.2 |  |  | 6.45 | 0.4 |  |  | 0.1 |
| Dimaprit | **162** |  | 6.5 | 6.1 | 6.4 |  |  | 6.1 | 5.7 |  |  | 0.0 | 0.7 |
| Imetit | **188** |  |  | 8.3 | 8.2 |  |  | 9.25 | 8.55 |  |  | 1.0 | 0.3 |
| Immepip | **190** |  |  | 9.2 | 7.7 |  |  | 9.25 | 6.3 |  |  | 0.0 | 1.4 |
| Iodophenpropit | **194** |  |  | 6.9 | 7.8 |  |  |  | 8.3 |  |  |  | 0.5 |
| N-methylhistamine | **233** |  |  | 8.8 | 7.6 |  |  | 8.95 | 7.45 |  |  | 0.1 | 0.2 |
| R-α-methylhistamine | **250** |  |  | 8.6 | 6.5 |  |  | 9.2 | 7.2 |  |  | 0.6 | 0.7 |
| Thioperamide | **265** |  |  | 9.0 | 7.3 |  |  | 7.4 | 6.95 |  |  | 1.6 | 0.4 |
| 2-methylhistamine | **81** | 6.1 |  |  |  |  |  |  | 6.35 |  |  |  |  |
| 4-methylhistamine | **96** |  | 5.4 |  | 7.4 |  |  |  | 7.75 |  |  |  | 0.4 |
| 9-OH-risperidone | **106** | 7.7 | 6.9 |  | 5.1 | 7.5 |  |  |  | 0.2 |  |  |  |
| A-304121 | **107** |  |  | 6.1 |  |  |  | 6.1 |  |  |  | 0.0 |  |
| A-331440 | **109** | 5.5 |  |  |  |  |  | 8.5 |  |  |  |  |  |
| Amitriptyline | **116** | 9.1 |  |  | 7.5 | 9.3 |  |  |  | 0.2 |  |  |  |
| Amthamine | **119** |  | 5.2 |  |  |  | 6.4 |  |  |  | 1.2 |  |  |
| ***Ligand name*** | ***Ligand no.*** | ***hH₁R*** | ***hH₂R*** | ***hH₃R*** | ***hH₄R*** | ***hH₁R*** | ***hH₂R*** | ***hH₃R*** | ***hH₄R*** | ***hH₁R variation*** | ***hH₂R variation*** | ***hH₃R variation*** | ***hH₄R variation*** |
| Aripiprazole | **120** | 7.5 |  |  |  | 7.5 |  |  |  | 0.0 |  |  |  |
| Astemizole | **122** | 9.1 |  |  |  | 8.5 |  |  |  | 0.6 |  |  |  |
| BU-E 47 | **126** | 6.6 |  |  |  | 6.6 |  |  |  | 0.0 |  |  |  |
| Cetirizine | **130** | 8.2 |  |  |  | 8.2 |  |  |  | 0.0 |  |  |  |
| Chlorpheniramine | **140** | 8.6 |  |  | 5.5 | 6.55 |  |  |  | 2.1 |  |  |  |
| Chlorpromazine | **141** | 9.7 | 6.8 |  | 5.6 | 8.2 |  |  |  | 1.5 |  |  |  |
| Cimetidine | **142** |  | 6.2 |  |  |  | 5.9 |  |  |  | 0.3 |  |  |
| Cipralisant | **145** |  |  | 8.3 | 7.3 |  |  | 8.6 |  |  |  | 0.3 |  |
| cis-H_2_-PAT(+) | **147** | 6.9 |  |  |  | 6.9 |  |  |  | 0.0 |  |  |  |
| CL-6-APT | **151** | 5.9 |  |  |  |  |  |  |  |  |  |  |  |
| Cyproheptadine | **159** | 10.2 |  |  | 6.7 | 10.2 |  |  |  | 0.0 |  |  |  |
| Dimethylhistaprodifen | **164** | 6.4 |  |  |  | 6.4 |  |  |  | 0.0 |  |  |  |
| Diphenhydramine | **165** | 7.9 |  |  |  | 7.9 |  |  |  | 0.0 |  |  |  |
| Doxepin | **168** | 9.8 |  |  | 7.0 | 9.75 |  |  |  | 0.0 |  |  |  |
| Famotidine | **171** |  | 7.8 |  |  |  | - |  |  |  |  |  |  |
| Fexofenadine | **172** | 8.0 |  |  |  | 7.6 |  |  |  | 0.4 |  |  |  |
| Fluphenazine | **175** | 7.7 | 6.3 |  |  | 7.7 |  |  |  | 0.0 |  |  |  |
| Fluspirilene | **179** | 7.9 |  |  |  | 7.9 |  |  |  | 0.0 |  |  |  |
| Haldol | **182** | 5.5 |  |  |  | 5.85 |  |  |  | 0.3 |  |  |  |
| Haloperidol | **185** | 5.9 | 6.0 |  |  | 5.85 |  |  |  | 0.1 |  |  |  |
| Imbutamine | **187** |  |  | 8.4 | 8.0 |  |  | 8.4 |  |  |  | 0.0 |  |
| Immethridine | **191** |  |  | 9.1 | 6.6 |  |  | 9.1 |  |  |  | 0.0 |  |
| Impentamine | **192** |  |  | 8.3 | 6.6 |  |  |  | 8.3 |  |  |  | 1.7 |
| JNJ 7777120 | **196** |  |  | 5.3 | 7.8 |  |  |  | 8.05 |  |  |  | 0.3 |
| JNJ-5207852 | **198** |  |  | 9.2 |  |  |  | 9.2 |  |  |  | 0.0 |  |
| Levocetirizine | **200** | 8.5 |  |  |  | 8.5 |  |  |  | 0.0 |  |  |  |
| Loratidine | **202** | 6.8 |  |  |  | 7.4 |  |  |  | 0.6 |  |  |  |
| Loxapine | **206** | 8.2 | 6.7 |  | 5.3 | 8.2 |  |  |  | 0.0 |  |  |  |
| ***Ligand name*** | ***Ligand no.*** | ***hH₁R*** | ***hH₂R*** | ***hH₃R*** | ***hH₄R*** | ***hH₁R*** | ***hH₂R*** | ***hH₃R*** | ***hH₄R*** | ***hH₁R variation*** | ***hH₂R variation*** | ***hH₃R variation*** | ***hH₄R variation*** |
| Mepyramine | **210** | 8.8 |  |  |  | 8.85 |  |  |  | 0.1 |  |  |  |
| Methylhistaprodifen | **212** | 6.4 |  |  |  | 6.4 |  |  |  | 0.0 |  |  |  |
| Mizolastine | **217** | 9.1 |  |  |  | 5.7 |  |  |  | 3.4 |  |  |  |
| Molindone | **222** | 5.7 |  |  |  |  |  |  |  |  |  |  |  |
| N-α-methylhistamine | **226** |  |  | 8.4 | 6.5 |  |  |  | 7 |  |  |  | 0.5 |
| Olanzapine | **237** | 8.8 | 7.4 |  |  | 8.95 |  |  |  | 0.2 |  |  |  |
| ORG-5222 (Asenapine) | **238** | 9.8 | 8.6 |  |  | 9.8 |  |  |  | 0.0 |  |  |  |
| Perphenazine | **239** | 8.1 |  |  |  | 8.1 |  |  |  | 0.0 |  |  |  |
| Pheniramine | **240** |  |  |  | 5.4 |  |  |  |  |  |  |  |  |
| Pimozide | **241** | 6.2 |  |  |  | 6.2 |  |  |  | 0.0 |  |  |  |
| Pipamerone | **242** | 5.6 |  |  |  | 5.6 |  |  |  | 0.0 |  |  |  |
| Promethazine | **244** | 9.6 |  |  | 6.8 | 9.6 |  |  |  | 0.0 |  |  |  |
| Proxyfan | **245** |  | 7.9 | 8.3 | 7.3 |  |  |  | 8.45 |  |  |  | 1.1 |
| Quetiapine | **246** | 8.1 |  |  |  | 8.35 |  |  |  | 0.3 |  |  |  |
| Ranitidine | **254** |  | 7.1 |  |  |  | 7.1 |  |  |  | 0.0 |  |  |
| Risperidone | **255** | 7.6 | 6.9 |  |  | 7.7 |  |  |  | 0.1 |  |  |  |
| Sertindole | **256** | 6.9 |  |  |  | 5.7 |  |  |  | 1.2 |  |  |  |
| SR 147778 | **263** | 6.0 | 6.0 | 6.0 |  | 7.7 |  |  |  | 1.7 |  |  |  |
| Terfenadine | **264** | 8.6 |  |  |  | 7.4 |  |  |  | 1.2 |  |  |  |
| Thioridazine | **266** | 7.8 | 6.9 |  | 5.6 | 9.2 |  |  |  | 1.4 |  |  |  |
| Thiothixene | **267** | 8.1 | 6.4 |  |  | 8.4 |  |  |  | 0.3 |  |  |  |
| Tiotidine | **268** |  | 7.8 |  |  |  |  |  |  |  |  |  |  |
| trans-H_2_-PAT(-) | **269** | 8.7 |  |  |  | 8.7 |  |  |  | 0.0 |  |  |  |
| trans-H_2_-PAT(+) | **270** | 7.4 |  |  |  | 7.45 |  |  |  | 0.0 |  |  |  |
| trans-H_2_-PAT(+/-) | **271** | 8.5 |  |  |  | 8.5 |  |  |  | 0.0 |  |  |  |
| Trifluoperazine | **303** | 7.2 |  |  |  | 7.2 |  |  |  | 0.0 |  |  |  |
| Triprolidine | **304** | 9.0 |  |  |  | 8.75 |  |  |  | 0.2 |  |  |  |
| Ziprasidone |  | 7.3 | 5.5 |  |  | 7.6 |  |  |  | 0.3 |  |  |  |
| ***Ligand name*** | ***Ligand no.*** | ***hH₁R*** | ***hH₂R*** | ***hH₃R*** | ***hH₄R*** | ***hH₁R*** | ***hH₂R*** | ***hH₃R*** | ***hH₄R*** | ***hH₁R variation*** | ***hH₂R variation*** | ***hH₃R variation*** | ***hH₄R variation*** |
| Amoxapine | **117** | 5.3 |  |  |  |  |  |  |  |  |  |  |  |
| CF3-5-APT | **134** | 5.2 |  |  |  |  |  |  |  |  |  |  |  |
| CF3-6-APT | **135** | 5.6 |  |  |  |  |  |  |  |  |  |  |  |
| Zotepine |  | 8.5 | 6.3 |  | 5.7 |  |  |  |  |  |  |  |  |
| SYA 09 (-) | **5** | 6.6 |  |  |  |  |  |  |  |  |  |  |  |
| 7-OH-DPAT | **101** | 5.8 |  |  |  |  |  |  |  |  |  |  |  |
| Atomoxetine | **123** | 6.0 |  |  |  |  |  |  |  |  |  |  |  |
| Butaclamol,(+) | **128** | 6.8 |  |  |  |  |  |  |  |  |  |  |  |
| Cariprazine | **129** | 7.6 |  |  |  |  |  |  |  |  |  |  |  |
| Cinnarizine | **143** |  |  |  | 6.8 |  |  |  |  |  |  |  |  |
| cis-H_2_-PAT(+/-) | **149** | 7.8 |  |  |  |  |  |  |  |  |  |  |  |
| Citalopram | **150** | 6.5 |  |  |  |  |  |  |  |  |  |  |  |
| Clemizole | **152** |  |  |  | 6.4 |  |  |  |  |  |  |  |  |
| Cocaine | **156** | 5.7 |  |  |  |  |  |  |  |  |  |  |  |
| Desipramine | **161** | 7.4 |  |  |  |  |  |  |  |  |  |  |  |
| Duloxetine | **170** | 5.6 |  |  |  |  |  |  |  |  |  |  |  |
| Flupenthixol, α | **173** | 9.1 |  |  |  |  |  |  |  |  |  |  |  |
| Flupenthixol, β | **174** | 8.2 |  |  |  |  |  |  |  |  |  |  |  |
| GBR-12909 (Vanoxerine) | **180** | 8.0 |  |  |  |  |  |  |  |  |  |  |  |
| Imipramine | **189** | 7.4 |  |  |  |  |  |  |  |  |  |  |  |
| Iodoproxyfan | **195** |  |  | 9.2 | 7.9 |  |  | 10 |  |  |  | 0.8 |  |
| Ketanserin | **199** | 8.7 |  |  |  |  |  |  |  |  |  |  |  |
| Loperamide | **201** | 5.7 |  |  |  |  |  |  |  |  |  |  |  |
| Mazindol | **207** | 6.2 |  |  |  |  |  |  |  |  |  |  |  |
| MDMA | **209** | 5.7 |  |  |  |  |  |  |  |  |  |  |  |
| Methysergide | **213** | 5.5 |  |  |  |  |  |  |  |  |  |  |  |
| Mianserin | **214** | 9.4 |  |  | 6.1 |  |  |  |  |  |  |  |  |
| Mirtazapine | **215** | 8.8 |  |  |  |  |  |  |  |  |  |  |  |
| ***Ligand name*** | ***Ligand no.*** | ***hH₁R*** | ***hH₂R*** | ***hH₃R*** | ***hH₄R*** | ***hH₁R*** | ***hH₂R*** | ***hH₃R*** | ***hH₄R*** | ***hH₁R variation*** | ***hH₂R variation*** | ***hH₃R variation*** | ***hH₄R variation*** |
| Mitragynine | **216** |  |  | 5.1 |  |  |  |  |  |  |  |  |  |
| MRS5698 | **225** |  |  |  | 5.0 |  |  |  |  |  |  |  |  |
| N-desalkylquetiapine | **227** | 8.5 | 6.5 |  | 5.8 |  |  |  |  |  |  |  |  |
| N-desmethylclozapine | **228** | 8.5 | 6.5 |  | 6.0 |  |  |  |  |  |  |  |  |
| Nefazodone | **232** | 6.4 |  |  |  |  |  |  |  |  |  |  |  |
| Pramipexole | **243** |  | 5.6 |  |  |  |  |  |  |  |  |  |  |
| Raclopride | **248** | 5.1 |  |  |  |  |  |  |  |  |  |  |  |
| Sertraline | **259** | >5.301 |  |  |  |  |  |  |  |  |  |  |  |
| SKF 38393 | **260** | 5.6 |  |  |  |  |  |  |  |  |  |  |  |
| Spiperone | **261** | 6.6 |  |  |  |  |  |  |  |  |  |  |  |
| Trazodone | **272** | 6.7 | 5.5 |  |  |  |  |  |  |  |  |  |  |

***Supplemental Table 9****: Organ-specific binding affinities (pK_i_) for hH_x_R. Reported is the ligand, the receptor, the human native tissue, the binding affinity (pK_i_) of the native tissue and of recombinant hH_x_R, as well as for each substance the range of pK_i_ values in the available tissues, and the deviation between. Ligands are sorted in ascending order of their ligand number. A visualization can be found in Figure 7.*

| **Ligand name** | **Ligand no.** | **hH_x_R** | **Human native tissue** | **pK_i_ hH_x_R native tissue** | **pK_i_ range duplicates native tissue** | **deviation native tissues (pK_i_)** | **pK_i_ recombinant hH_x_R** | **Deviation recombinant hH_x_R vs. native tissue (pK_i_)** |
| --- | --- | --- | --- | --- | --- | --- | --- | --- |
| 9-OH-risperidone | **106** | hH₁R | brain | 8.5 |  | - | 7.7 | 0.77 |
| A-304121 | **107** | hH₃R | cortex | 6.1 |  | - | 6.1 | 0.01 |
| A-317920 | **108** | hH₃R | cortex | 6.9 |  | - | 7 | 0.07 |
| A-331440 | **109** | hH₃R | cerebral cortex | 7.6 |  | - | <5 | >2.6 |
| A-349821 | **110** | hH₃R | cortex | 9.4 |  | - | 9.4 | 0.03 |
| ABT-239 | **112** | hH₃R | cortex | 8.3 |  | - | 9.3 | 0.96 |
| Amitriptyline | **116** | hH₁R | cortical membranes | 9.2 |  | 0.2 | 9.1 | 0.07 |
| Amitriptyline | **116** | hH₁R | brain | 9 |  |  | 9.1 | 0.08 |
| Amitriptyline | **116** | hH₁R | frontal cortex | 9 |  |  | 9.1 | 0.14 |
| ***Ligand name*** | ***Ligand no.*** | ***hH_x_R*** | ***Human native tissue*** | ***pK_i_ hH_x_R native tissue*** | ***pK_i_ range duplicates native tissue*** | ***deviation native tissues (pK_i_)*** | ***pK_i_ recombinant hH_x_R*** | ***Deviation recombinant hH_x_R vs. native tissue (pK_i_)*** |
| Chlorpheniramine | **134** | hH₁R | frontal cortex | 8.5 |  | - | 8.6 | 0.08 |
| Chlorpromazine | **135** | hH₁R | cortical membranes | 8.5 |  | 0.5 | 9.7 | 1.18 |
| Cimetidine | **140** | hH₁R | cortical membranes | <5 |  | - | no value | - |
| Cipralisant | **142** | hH₃R | cortex | 7.9 |  | - | 8.3 | 0.39 |
| Ciproxifan | **143** | hH₃R | cortex | 7.1 |  | - | 7.2 | 0.15 |
| Clobenpropit | **151** | hH₃R | cortex | 9.1 |  | - | 7.2 | 1.91 |
| Clozapine | **152** | hH₁R | cortical membranes | 8.9 |  | 0.1-0.4 | 8.8 | 0.12 |
| Clozapine | **152** | hH₁R | brain | 8.6 |  |  | 8.8 | 0.24 |
| Clozapine | **152** | hH₁R | frontal cortex | 8.5-8.7 | 0.17 |  | 8.8 | 0.08-0.25 |
| Diphenhydramine | **162** | hH₁R | frontal cortex | 7.8 |  | 0.6 | 7.9 | 0.08 |
| Diphenhydramine | **162** | hH₁R | cortical membranes | 7.2 |  |  | 7.9 | 0.69 |
| Doxepine | **164** | hH₁R | brain | 9.8 |  | 0.1-0.5 | 9.8 | 0.03 |
| Doxepine | **164** | hH₁R | frontal cortex | 9.4 |  |  | 9.8 | 0.4 |
| Doxepine | **164** | hH₁R | cortical membranes | 9.3 |  |  | 9.8 | 0.49 |
| Fluphenazine | **173** | hH₁R | frontal cortex | 7.2-7.7 | 0.52 |  | 7.7 | 0.02-0.55 |
| Haloperidol | **180** | hH₁R | frontal cortex | 5.6 |  | 0.1-1.0 | 5.9 | 0.3 |
| Haloperidol | **180** | hH₁R | brain | 5.7-6.5 | 0.86 |  | 5.9 | 0.18-0.69 |
| Histamine | **182** | hH₁R | frontal cortex | 4.65-<5 | >0.34 | 0.0-3.95 | 5.9 | 1.25 |
| Histamine | **182** | hH₁R | cortical membranes | <5 |  |  | 5.9 | >0.9 |
| Histamine | **182** | hH₃R | cerebral cortex | 8.6 |  |  | 6.4 | 2.23 |
| Loxapine | **201** | hH₁R | brain | 8.3 |  | 0 | 8.2 | 0.1 |
| Loxapine | **201** | hH₁R | frontal cortex | 8.3 |  |  | 8.2 | 0.11 |
| Mepyramide | **207** | hH₁R | frontal cortex | 8.3-9.0 | 0.67 |  | 8.8 | 0.2-0.47 |
| ***Ligand name*** | ***Ligand no.*** | ***hH_x_R*** | ***Human native tissue*** | ***pK_i_ hH_x_R native tissue*** | ***pK_i_ range duplicates native tissue*** | ***deviation native tissues (pK_i_)*** | ***pK_i_ recombinant hH_x_R*** | ***Deviation recombinant hH_x_R vs. native tissue (pK_i_)*** |
| Molindone | **216** | hH₁R | cortical membranes | <5 |  | ? | 5.7 | >0.7 |
| Molindone | **216** | hH₁R | frontal cortex | <5 |  |  | 5.7 | >0.7 |
| Olanzapine | **232** | hH₁R | brain | 10.1 |  | - | 5.9 | 4.16 |
| Perphenazine | **237** | hH₁R | frontal cortex | 8.1 |  | 0 | 8.1 | 0 |
| Perphenazine | **237** | hH₁R | brain | 8.1 |  |  | 8.1 | 0.02 |
| Pimozide | **239** | hH₁R | brain | 7.6 |  | - | 6.2 | 1.4 |
| Promethazine | **242** | hH₁R | frontal cortex | 8.6 |  | - | 8.8 | 0.21 |
| Proxyfan | **243** | hH₃R | cortex | 8.4 |  | - | 8.3 | 0.08 |
| Quetiapine | **244** | hH₁R | brain | 7.7 |  | - | 8.1 | 0.38 |
| Ranitidine | **248** | hH₁R | cortical membranes | <5 |  | - | <5 | - |
| Sertindole | **254** | hH₁R | brain | 6.5 |  | - | 6.9 | 0.41 |
| Thioperamide | **263** | hH₃R | cerebral cortex | 7.2 |  | 0 | 9 | 1.79 |
| Thioperamide | **263** | hH₃R | cortex | 7.2 |  | - | 9 | 1.82 |
| Thioridazine | **264** | hH₁R | frontal cortex | 7.7-7.8 | 0.14 | - | 7.8 | 0.004-0.14 |
| Thiothixidene | **265** | hH₁R | cortical membranes | 8.4 |  | - | 8.1 | 0.26 |
| Tiotidine | **267** | hH₁R | cortical membranes | <5 |  | - | no value | - |
| Trifluoperazine | **271** | hH₁R | frontal cortex | 7.2 |  | - | 7.2 | 0.01 |
| Triprolidine | **272** | hH₁R | frontal cortex | 8.4 |  | - | 9 | 0.57 |
| Ziprasidone | **303** | hH₁R | brain | 8.3 |  | - | 7.3 | 1.04 |

***Supplemental Table 10****: Species-specific binding affinities (pK_i_) for H_x_R. Reported is the ligand, the respective H_x_R of the non-human species and its examined native tissue, the binding affinity (pK_i_) of the species and of recombinant hH_x_R, as well as for each substance the range of pK_i_ values in the available tissues, and the deviation of recombinant hH_x_R vs native tissues. Ligands are sorted in ascending order of their ligand number. A visualization can be found in Figure 8.*

| **Ligand name** | **Ligand no.** | **H_x_R** | **Species** | **Native tissue** | **pK_i_ H_x_R native tissue** | **pK_i_ range duplicates species** | **pK_i_ recom-binant hH_x_R** | ***Deviation recombinant hH_x_R vs. native tissue (pK_i_)*** |
| --- | --- | --- | --- | --- | --- | --- | --- | --- |
| (3S,4S,6aS,9S,10S,12aS)-4,10-bis(4-(3-(dimethylamino)prop-1-ynyl)phenyl)-3,9-dimethyloctahydrodipyrido[1,2-a:1',2'-d]pyrazine-6,12(6aH,12aH)-dione | **26** | H₃R | guinea pig | cloned | 5.3 |  | not listed | - |
| 2-(2-Thiazolyl)ethanamine | **71** | H₁R | guinea pig | cloned | 5.1 |  | 5.3 | 0.23 |
| 2-{4-[4-(7-Methoxy-naphthalen-1-yl)piperazin-1-yl]-butyl}-4-methyl-2H-[1,2,4]triazine-3,5-dione | **78** | H₃R | guinea pig | cloned | <5 |  | not listed | - |
| 2-methoxy-diphenidine | **80** | H₃R | guinea pig | cloned | <5 |  | not listed | - |
| 3-methoxy-diphenidine | **87** | H₃R | guinea pig | cloned | 5.8 |  | not listed | - |
| 4-methoxy-diphenidine | **95** | H₃R | guinea pig | cloned | 6.3 |  | not listed | - |
| 9-OH-risperidone | **106** | H₁R | guinea pig | cerebellum | 8 |  | 7.7 | 0.3 |
| 9-OH-risperidone | **106** | H₃R | guinea pig | cloned | <5 |  | <5 | - |
| A-304121 | **107** | H₃R | dog | brain cortex | 7.3 |  | 6.1 | 1.23 |
| A-304121 | **107** | H₃R | guinea pig | brain cortex | 7.8 |  | 6.1 | 1.66 |
| A-317920 | **108** | H₃R | dog | brain cortex | 8.4 |  | 7 | 1.37 |
| A-317920 | **108** | H₃R | guinea pig | brain cortex | 8.6 |  | 7 | 1.62 |
| A-349821 | **110** | H₃R | dog | brain cortex | 8.9 |  | 9.4 | 0.48 |
| A-349821 | **110** | H₃R | guinea pig | brain cortex | 9.3 |  | 9.4 | 0.14 |
| ABT-239 | **112** | H₃R | dog | brain cortex | 8.4 |  | 9.3 | 0.89 |
| ABT-239 | **112** | H₃R | guinea pig | brain cortex | 8.6 |  | 9.3 | 0.71 |
| R-α-methylhistamine | **113** | H₃R | guinea pig | lung | 6.1 |  | 8.4 | 2.31 |
| R-α-methylhistamine | **113** | H₄R | guinea pig | cortex | 10 |  | 6.8 | 3.2 |
| ***Ligand name*** | ***Ligand no.*** | ***H_x_R*** | ***Species*** | ***Native tissue*** | ***pK_i_ H_x_R native tissue*** | ***pK_i_ range duplicates species*** | ***pK_i_ recom-binant hH_x_R*** | ***Deviation recombinant hH_x_R vs. native tissue (pK_i_)*** |
| R-α-methylhistamine | **113** | H₄R | guinea pig | myenteric plexus | 9.8 |  | 6.8 | 3.02 |
| S-α-methylhistamine | **114** | H₃R | guinea pig | lung | 7.3 |  | 7.2 | 0.08 |
| Amitriptyline | **116** | H₁R | guinea pig | brain | 8.8-9.8 | 0.97 | 9.1 | 0.3-0.67 |
| Aripiprazole | **120** | H₃R | guinea pig | cloned | 6.6 |  | not listed | - |
| Arpromidine | **121** | H₁R | guinea pig | cloned | 7.5 |  | 6.5 | 0.98 |
| Astemizole | **122** | H₁R | guinea pig | cerebellum | 9.1 |  | 9.1 | 0 |
| ATR | **124** | H₁R | guinea pig | brain | 5.7 |  | 5.9 | 0.16 |
| BU-E 47 | **126** | H₁R | guinea pig | cloned | 7.3 |  | 6.6 | 0.67 |
| Burimamide | **127** | H₂R | guinea pig | cerebral cortex | 5.5 |  | 5.7 | 0.18 |
| Butaclamol, (+) | **128** | H₁R | guinea pig | cerebellum | 5.5 |  | 6.8 | 1.29 |
| Cetirizine | **130** | H₁R | guinea pig | cerebellum | 7.9 |  | 8.2 | 0.3 |
| Chlorpheniramine | **134** | H₁R | guinea pig | brain | 9.4 |  | 8.6 | 0.76 |
| Chlorpheniramine | **134** | H₁R | guinea pig | cerebellum | 9.3 |  | 8.6 | 0.7 |
| Chlorpromazine | **135** | H₁R | guinea pig | brain | 8.3 |  | 7 | 1.35 |
| Chlorpromazine | **135** | H₃R | guinea pig | cloned | <5 |  | not listed | - |
| cimetidine | **140** | H₂R | guinea pig | cerebral cortex | 5.3 |  | 6.8 | 1.48 |
| Cipralisant | **142** | H₃R | dog | brain cortex | 8.9 |  | 8.3 | 0.58 |
| Cipralisant | **142** | H₃R | guinea pig | brain cortex | 9.2 |  | 8.3 | 0.9 |
| Ciproxifan | **143** | H₃R | dog | brain cortex | 8.2 |  | 7.2 | 1.04 |
| Ciproxifan | **143** | H₃R | guinea pig | cortex | 8.8 |  | 7.2 | 1.56 |
| cis-H_2_-PAT(-) | **144** | H₁R | guinea pig | cloned | 8.3-8.4 | 0.1 | 8.1 | 0.21-0.31 |
| cis-H_2_-PAT(+) | **145** | H₁R | guinea pig | cloned | 7.1-7.3 | 0.16 | 6.9 | 0.21-0.37 |
| cis-H_2_-PAT(+/-) | **146** | H₁R | guinea pig | cloned | 8.0-8.1 | 0.14 | 7.8 | 0.16-0.3 |
| Citalopram | **147** | H₁R | guinea pig | brain | 6.5 |  | 6.5 | 0.05 |
| ***Ligand name*** | ***Ligand no.*** | ***H_x_R*** | ***Species*** | ***Native tissue*** | ***pK_i_ H_x_R native tissue*** | ***pK_i_ range duplicates species*** | ***pK_i_ recom-binant hH_x_R*** | ***Deviation recombinant hH_x_R vs. native tissue (pK_i_)*** |
| Clobenpropit | **151** | H₃R | dog | brain cortex | 10.1 |  | 7.2 | 2.89 |
| Clobenpropit | **151** | H₃R | guinea pig | brain cortex | 9.6 |  | 7.2 | 2.45 |
| Clozapine | **152** | H₁R | guinea pig | cerebellum | 7.6-8.7 | 1.04 | 8.8 | 0.12-1.16 |
| Clozapine | **152** | H₁R | guinea pig | cloned | 8.7-8.9 | 0.23 | 8.8 | 0.11-0.16 |
| Clozapine | **152** | H₁R | guinea pig | brain | 8.4 |  | 8.8 | 0.4 |
| Clozapine | **152** | H₃R | guinea pig | cloned | 5.2 |  | <5 | >0.2 |
| Cocaine | **153** | H₃R | guinea pig | cloned | <5 |  | <5 | - |
| Cyproheptadine | **154** | H₁R | guinea pig | cerebellum | 10 |  | 10.2 | 0.2 |
| D-chlorpheniramine | **155** | H₁R | guinea pig | cloned | 8.7-9.2 | 0.46 | 8.4 | 0.3-0.76 |
| D-chlorpheniramine | **155** | H₁R | guinea pig | brain | 8.9 |  | 8.4 | 0.45 |
| D-chlorpheniramine | **155** | H₁R | mouse | brain | 8 |  | 8.4 | 0.36 |
| D-chlorpheniramine | **155** | H₁R | rabbit | brain | 7.7 |  | 8.4 | 0.72 |
| Desipramine | **156** | H₁R | guinea pig | brain | 6.6-7.5 | 0.91 | 7.4 | 0.11-0.21 |
| Dimaprit | **159** | H₂R | guinea pig | cerebral cortex | <5 |  | 6.5 | >1.5 |
| Diphenhydramine | **162** | H₁R | guinea pig | cloned | 8.0-8.0 | 0.02 | 7.9 | 0.12-0.14 |
| Diphenhydramine | **162** | H₁R | guinea pig | brain | 7.9 |  | 7.9 | 0.04 |
| Diphenidine | **163** | H₃R | guinea pig | cloned | 6 |  | not listed | - |
| Doxepine | **164** | H₁R | guinea pig | cloned | 9.6-9.8 | 0.24 | 9.8 | 0.004-0.247 |
| Doxepine | **164** | H₁R | guinea pig | brain | 9.3 |  | 9.8 | 0.5 |
| Duloxetine | **165** | H₂R | guinea pig | cerebral cortex | 6 |  | not listed | - |
| Escitalopram | **167** | H₁R | guinea pig | brain | 5.7 |  | 5.7 | 0 |
| Fluphenazine | **173** | H₁R | guinea pig | brain | 7.7 |  | 7.7 | 0.02 |
| Fluphenazine | **173** | H₃R | guinea pig | cloned | <5 |  | not listed | - |
| Fluspirilene | **174** | H₁R | guinea pig | cerebellum | 6.3 |  | 7.9 | 1.63 |
| Haloperidol | **180** | H₁R | guinea pig | cerebellum | 5.7-6.1 | 0.39 | 5.9 | 0.155-0.24 |
| ***Ligand name*** | ***Ligand no.*** | ***H_x_R*** | ***Species*** | ***Native tissue*** | ***pK_i_ H_x_R native tissue*** | ***pK_i_ range duplicates species*** | ***pK_i_ recom-binant hH_x_R*** | ***Deviation recombinant hH_x_R vs. native tissue (pK_i_)*** |
| Haloperidol | **180** | H₁R | guinea pig | brain | 6.1 |  | 5.9 | 0.2 |
| HIS | **181** | H₁R | guinea pig | Sf9 | 5.3 |  | 6 | 0.66 |
| HIS | **181** | H₂R | guinea pig | Sf9 | 5.9 |  | 5.9 | 0.02 |
| Histamine | **182** | H₁R | guinea pig | brain | 7.0-7.6 | 0.57 | 5.9 | 1.1-1.7 |
| Histamine | **182** | H₁R | guinea pig | cloned | 5.0-<5 | >0.0 | 5.9 | >0.9 |
| Histamine | **182** | H₁R | guinea pig | Sf9 | 5.3 |  | 5.9 | 0.56 |
| Histamine | **182** | H₁R | guinea pig | cerebellum | 5.5 |  | 5.9 | 0.4 |
| Histamine | **182** | H₂R | guinea pig | cerebral cortex | <5 |  | 7.8 | >2.8 |
| Histamine | **182** | H₂R | guinea pig | cortical membranes | 7.0 |  | 7.8 | 0.8 |
| Histamine | **182** | H₃R | guinea pig | cerebral cortex | 7.0 |  | 8.3 | 1.3 |
| Histamine | **182** | H₃R | guinea pig | lung | 7.0 |  | 8.3 | 1.3 |
| Histamine | **182** | H₄R | guinea pig | cortex | 9.9 |  | 7.8 | 2.05 |
| Histamine | **182** | H₄R | guinea pig | myenteric plexus | 7.0 |  | 7.8 | 0.82 |
| Impromidine | **191** | H₂R | guinea pig | cerebral cortex | 7.2 |  | 6.6 | 0.6 |
| Iodophenpropit | **192** | H₁R | guinea pig | cloned | 5.8 |  | not listed | - |
| Iodophenpropit | **192** | H₂R | guinea pig | cloned | 5.6 |  | not listed | - |
| I-chlorpheniramine | **197** | H₁R | guinea pig | cloned | 7.0-7.1 | 0.09 | 7 | 0.009-0.09 |
| I-chlorpheniramine | **197** | H₁R | mouse | brain | 6.1 |  | 7 | 0.86 |
| I-chlorpheniramine | **197** | H₁R | rabbit | brain | 5.7 |  | 7 | 1.32 |
| mCPP | **203** | H₁R | guinea pig | brain | 6.3 |  | 6.5 | 0.15 |
| Mepyramine | **207** | H₁R | guinea pig | cloned | 9.1-9.2 | 0.11 | 8.8 | 0.33-0.44 |
| Mepyramine | **207** | H₁R | guinea pig | brain | 9.3 |  | 8.8 | 0.5 |
| Mepyramine | **207** | H₁R | guinea pig | cerebellum | 8.9 |  | 8.8 | 0.1 |
| ***Ligand name*** | ***Ligand no.*** | ***H_x_R*** | ***Species*** | ***Native tissue*** | ***pK_i_ H_x_R native tissue*** | ***pK_i_ range duplicates species*** | ***pK_i_ recom-binant hH_x_R*** | ***Deviation recombinant hH_x_R vs. native tissue (pK_i_)*** |
| Mepyramine | **207** | H₁R | mouse | brain | 8.5 |  | 8.8 | 0.31 |
| Mepyramine | **207** | H₁R | rabbit | brain | 8.4 |  | 8.8 | 0.38 |
| Methysergide | **210** | H₁R | guinea pig | brain | 5.5 |  | 5.5 | 0.02 |
| Mianserin | **212** | H₁R | guinea pig | lung | 8.4 |  | 9.4 | 1.02 |
| Mirtazapine | **213** | H₁R | guinea pig | lung | 8.3 |  | 8.8 | 0.51 |
| N-desmethylclozapine | **226** | H₃R | guinea pig | cloned | <5 |  | not listed | - |
| Nefazodone | **227** | H₁R | guinea pig | brain | 7.5 |  | 6.4 | 1.12 |
| Nortryptyline | **229** | H₁R | guinea pig | brain | 7.5 |  | 7.8 | 0.28 |
| Olanzapine | **232** | H₁R | guinea pig | cerebellum | 8.5 |  | 8.8 | 0.34 |
| Olanzapine | **232** | H₃R | guinea pig | cloned | 5.4 |  | not listed | - |
| ORG-5222 | **233** | H₁R | guinea pig | cerebellum | 9 |  | 9.8 | 0.75 |
| Pipamperone | **240** | H₁R | guinea pig | cerebellum | 5.3 |  | 5.6 | 0.3 |
| Pramipexol | **241** | H₃R | guinea pig | cloned | <5 |  | not listed | - |
| Promethazine | **242** | H₁R | guinea pig | brain | 8.5 |  | 9.6 | 1.11 |
| Quetiapine | **244** | H₁R | guinea pig | cerebellum | 7.7 |  | 8.1 | 0.38 |
| Quetiapine | **244** | H₃R | guinea pig | cloned | <5 |  | not listed | - |
| Ranitidine | **248** | H₂R | guinea pig | cerebral cortex | 6.4 |  | 7.1 | 0.69 |
| Risperidone | **250** | H₁R | guinea pig | cerebellum | 8.6 |  | 7.6 | 0.99 |
| Risperidone | **250** | H₃R | guinea pig | cloned | <5 |  | not listed | - |
| Sertindole | **254** | H₁R | guinea pig | cerebellum | 6.2 |  | 6.9 | 0.66 |
| Sertraline | **255** | H₁R | guinea pig | brain | 5.2-5.3 | 0.12 | <5.301 | >0.12 |
| Thioperamide | **263** | H₁R | guinea pig | cloned | <5 |  | <5 | - |
| Thioperamide | **263** | H₂R | guinea pig | cloned | <5 |  | <5 | - |
| Thioperamide | **263** | H₃R | dog | brain cortex | 8.2 |  | 9 | 0.83 |
| Thioperamide | **263** | H₃R | guinea pig | brain cortex | 8.3 |  | 9 | 0.66 |
| Thioperamide | **263** | H₃R | guinea pig | lung | 8.7 |  | 9 | 0.3 |
| ***Ligand name*** | ***Ligand no.*** | ***H_x_R*** | ***Species*** | ***Native tissue*** | ***pK_i_ H_x_R native tissue*** | ***pK_i_ range duplicates species*** | ***pK_i_ recom-binant hH_x_R*** | ***Deviation recombinant hH_x_R vs. native tissue (pK_i_)*** |
| Thioridazine | **264** | H₁R | guinea pig | brain | 7.5 |  | 7.8 | 0.33 |
| Thioridazine | **264** | H₃R | guinea pig | cloned | <5 |  | not listed | - |
| Tiotidine | **266** | H₂R | guinea pig | cerebral cortex | 7.5 |  | 7.8 | 0.33 |
| trans-H_2_-PAT(-) | **267** | H₁R | guinea pig | cloned | 8.9-9.2 | 0.3 | 7.4 | 1.54-1.84 |
| trans-H_2_-PAT(+) | **268** | H₁R | guinea pig | cloned | 7.6-7.9 | 0.3 | 8.7 | 0.76-1.06 |
| trans-H_2_-PAT(+/-) | **269** | H₁R | guinea pig | cloned | 8.8-8.9 | 0.05 | 8.5 | 0.32-0.36 |
| Trazodone | **270** | H₁R | guinea pig | brain | 7.5 |  | 6.7 | 0.84 |
| Trazodone | **270** | H₃R | guinea pig | cloned | <5 |  | not listed | - |
| Triprolidine | **272** | H₁R | guinea pig | cloned | 9.0-9.2 | 0.13 | 9 | 0.03-0.16 |
| Triprolidine | **272** | H₁R | guinea pig | cerebellum | 9.5 |  | 9 | 0.51 |
| Triprolidine | **272** | H₁R | guinea pig | brain | 9.2 |  | 9 | 0.22 |
| Triprolidine | **272** | H₁R | mouse | brain | 8 |  | 9 | 0.96 |
| Triprolidine | **272** | H₁R | rabbit | brain | 7.7 |  | 9 | 1.3 |
| UR-AK22 | **275** | H₁R | guinea pig | Sf9 | 6.4 |  | 5.5 | 0.86 |
| UR-AK22 | **275** | H₂R | guinea pig | Sf9 | 7.3 |  | 7.1 | 0.15 |
| UR-AK24 | **276** | H₁R | guinea pig | Sf9 | 5.6 |  | 5.3 | 0.29 |
| UR-AK24 | **276** | H₂R | guinea pig | Sf9 | 7.9 |  | 7.2 | 0.72 |
| UR-AK26 | **277** | H₂R | guinea pig | Sf9 | 7.1 |  | 6.823908741 | 0.3 |
| UR-AK41 | **278** | H₂R | guinea pig | Sf9 | 6.2 |  | 5.853871964 | 0.34 |
| UR-AK46 | **279** | H₂R | guinea pig | Sf9 | 7.7 |  | 7.420216403 | 0.26 |
| UR-AK49 | **280** | H₂R | guinea pig | Sf9 | 7.7 |  | 7.337242168 | 0.32 |
| UR-AK51 | **281** | H₂R | guinea pig | Sf9 | 7.6 |  | 7 | 0.64 |
| UR-AK57 | **282** | H₁R | guinea pig | Sf9 | 5.8 |  | 6.2 | 0.4 |
| ***Ligand name*** | ***Ligand no.*** | ***H_x_R*** | ***Species*** | ***Native tissue*** | ***pK_i_ H_x_R native tissue*** | ***pK_i_ range duplicates species*** | ***pK_i_ recom-binant hH_x_R*** | ***Deviation recombinant hH_x_R vs. native tissue (pK_i_)*** |
| UR-AK57 | **282** | H₂R | guinea pig | Sf9 | 8 |  | 7.6 | 0.45 |
| UR-AK59 | **283** | H₁R | guinea pig | Sf9 | 5 |  | 5.4 | 0.44 |
| UR-AK59 | **283** | H₂R | guinea pig | Sf9 | 7.4 |  | 7 | 0.43 |
| UR-AK62 | **284** | H₁R | guinea pig | Sf9 | 5.3 |  | 5.2 | 0.13 |
| UR-AK62 | **284** | H₂R | guinea pig | Sf9 | 7.6 |  | 7.2 | 0.44 |
| UR-AK64 | **285** | H₁R | guinea pig | Sf9 | 6.5 |  | 6 | 0.46 |
| UR-AK64 | **285** | H₂R | guinea pig | Sf9 | 7.8 |  | 7.6 | 0.2 |
| UR-AK67 | **286** | H₁R | guinea pig | Sf9 | 5.7 |  | <5 | >0.7 |
| UR-AK67 | **286** | H₂R | guinea pig | Sf9 | 7.7 |  | 7.2 | 0.48 |
| UR-AK68 | **287** | H₂R | guinea pig | Sf9 | 7.5 |  | 7.1 | 0.44 |
| Ziprasidone | **303** | H₁R | guinea pig | cerebellum | 8.3 |  | 7.3 | 0.98 |
| Ziprasidone | **303** | H₃R | guinea pig | cloned | <5 |  | not listed | - |
| Zotepine | **304** | H₁R | guinea pig | cerebellum | 8.5 |  | 8.5 | 0.03 |
| Zotepine | **304** | H₃R | guinea pig | cloned | <5 |  | not listed | - |

***Supplemental Table 11:*** *Overview of species-dependent differences in H_x_R, summarized from key literature. Information is provided about the topic of the primary literature, the considered species, and their most important findings.*

| **Reference** | **Topic** | **Receptor** | **Species** | **Key information** |
| --- | --- | --- | --- | --- |
| [44] | Pharmacological differences | H_1_R | human, guinea pig | differences in agonist and antagonist pharmacology, encompassing diverse classes of bulky ligands, possibly explainable by higher conformational flexibility of the guinea pig ortholog relative to the human ortholog |
| [1] | H_x_R overview and species variability | H_1_R- H_4_R | several | histamine receptor subtypes exhibit species-dependent differences in ligand affinity, efficacy, constitutive activity, receptor isoforms, and signal transduction; particularly pronounced for H_3_R and H_4_R, complicating comparison between animal models and human receptors |
| [4] | Species-dependent activities | H_1_R- H_4_R | several | all receptors exhibit pronounced differences in agonist and antagonist pharmacology with diverse ligands; role of ligand-binding sites, extracellular domains, receptor activation, constitutive receptor activity, thermodynamics, activation mechanisms, and ligand-specific receptor conformations |
| [133] | Pharmacological properties | H_3_R | human, rat | species-dependent ligand pharmacology with variation in potency and efficacy; significant role of amino acids in transmembrane domain 3, with differences in electrostatic surfaces causing differential orientation |
| [123] | Role of amino acids in transmembrane domain 3 | H_3_R | human, rat | receptors differ in five amino acid residues in transmembrane domains; distinct affinities of ligands; discrimination patterns were partially changed by mutation of single amino acids |
| [136] | Expression and functional properties | H_4_R | canine, rat, murine | differences in ligand affinity, potency in high-affinity GTPase studies, constitutive activity, efficacy, and functional behavior of ligands (namely JNJ7777120); strong variation in amino acid sequence position 341; differing regulation by NaCl |
| [134] | Molecular determinants of ligand binding | H_4_R | human, mouse, rat, guinea pig, monkey, pig, dog | strong differences in affinities of evaluated ligands (VUF 8430, VUF 6002, JNJ 7777120); variations in amino acid sequences |
| [42] | Pharmacological species variation | H_4_R | human, mouse, rat, guinea pig | strong differences in the human H_4_R sequence in comparison with non-human H_4_R; differences in binding affinities and signal transduction responses |

***Supplemental Table 12:*** *Identification of ligands in the heat maps (Figure 7, Supplemental Figure 3-5). The first column indicates the ligand numbering as shown on the y-axis of each heat map (graphical order). The corresponding original ligand identifiers are provided for Figure 6 (sorted by hH₁R), Supplemental Figure 2 (sorted by hH₂R), Supplemental Figure 3 (sorted by hH₃R), and Supplemental Figure 4 (sorted by hH₄R).*

| **Numbering of heat map (y-axis)** | **Figure 6 Ligand no. (heat map, sorted by hH₁R)** | **Supplemental Figure 2 Ligand no. (heat map, sorted by hH₂R)** | **Supplemental Figure 3 Ligand no. (heat map, sorted by hH₃R)** | **Supplemental Figure 4 Ligand no. (heat map, sorted by hH₄R)** |
| --- | --- | --- | --- | --- |
| 1 | **154** | **233** | **54** | **37** |
| 2 | **233** | **243** | **58** | **186** |
| 3 | **164** | **182** | **56** | **49** |
| 4 | **135** | **115** | **88** | **293** |
| 5 | **242** | **168** | **53** | **35** |
| 6 | **212** | **266** | **62** | **46** |
| 7 | **21** | **282** | **52** | **185** |
| 8 | **1** | **285** | **94** | **302** |
| 9 | **116** | **279** | **63** | **292** |
| 10 | **215** | **232** | **85** | **31** |
| 11 | **122** | **280** | **61** | **193** |
| 12 | **171** | **284** | **45** | **290** |
| 13 | **184** | **276** | **57** | **289** |
| 14 | **272** | **286** | **59** | **151** |
| 15 | **24** | **275** | **39** | **32** |
| 16 | **6** | **287** | **98** | **34** |
| 17 | **152** | **248** | **82** | **40** |
| 18 | **207** | **283** | **110** | **194** |
| 19 | **232** | **281** | **60** | **192** |
| 20 | **213** | **250** | **112** | **182** |
| 21 | **196** | **106** | **55** | **15** |
| 22 | **102** | **264** | **195** | **191** |
| 23 | **267** | **277** | **188** | **48** |
| 24 | **134** | **152** | **38** | **188** |
| 25 | **261** | **135** | **193** | **228** |
| 26 | **198** | **201** | **65** | **41** |
| 27 | **304** | **191** | **247** | **288** |
| 28 | **269** | **225** | **25** | **291** |
| 29 | **226** | **79** | **66** | **33** |
| 30 | **225** | **159** | **70** | **183** |
| 31 | **155** | **226** | **75** | **29** |
| 32 | **19** | **265** | **189** | **116** |
| 33 | **103** | **304** | **64** | **17** |
| 34 | **104** | **173** | **263** | **96** |
| 35 | **273** | **140** | **208** | **43** |
| 36 | **172** | **223** | **83** | **263** |
| 37 | **69** | **257** | **36** | **243** |
| 38 | **130** | **51** | **228** | **294** |
| 39 | **201** | **260** | **299** | **142** |
| 40 | **20** | **180** | **246** | **47** |
| 41 | **144** | **181** | **178** | **295** |
| 42 | **265** | **278** | **100** | **164** |
| 43 | **237** | **296** | **185** | **127** |
| 44 | **244** | **169** | **222** | **296** |
| 45 | **175** | **127** | **113** | **141** |
| 46 | **166** | **77** | **243** | **113** |
| 47 | **170** | **224** | **190** | **44** |
| 48 | **162** | **118** | **191** | **242** |
| 49 | **174** | **241** | **142** | **154** |
| 50 | **22** | **27** | **127** | **67** |
| 51 | **146** | **158** | **186** | **189** |
| 52 | **229** | **258** | **301** | **190** |
| 53 | **264** | **270** | **297** | **30** |
| 54 | **106** | **251** | **302** | **246** |
| 55 | **173** | **303** | **298** | **222** |
| 56 | **250** | **211** | **300** | **159** |
| 57 | **129** | **290** | **89** | **150** |
| 58 | **120** | **96** | **183** | **152** |
| 59 | **23** | **177** | **151** | **176** |
| 60 | **268** | **28** | **143** | **212** |
| 61 | **187** | **119** | **114** | **226** |
| 62 | **156** | **151** | **108** | **225** |
| 63 | **131** | **112** | **192** | **143** |
| 64 | **12** | **176** | **182** | **304** |
| 65 | **18** | **154** | **107** | **208** |
| 66 | **303** | **164** | **159** | **264** |
| 67 | **2** | **242** | **51** | **135** |
| 68 | **271** | **212** | **260** | **134** |
| 69 | **274** | **21** | **194** | **42** |
| 70 | **204** | **1** | **214** | **26** |
| 71 | **236** | **116** | **232** | **114** |
| 72 | **50** | **215** | **250** | **238** |
| 73 | **218** | **122** | **264** | **201** |
| 74 | **262** | **171** | **152** | **16** |
| 75 | **253** | **184** | **135** | **106** |
| 76 | **197** | **272** | **201** | **217** |
| 77 | **7** | **24** | **226** | **232** |
| 78 | **145** | **6** | **173** | **250** |
| 79 | **125** | **207** | **180** | **173** |
| 80 | **254** | **213** | **164** | **180** |
| 81 | **11** | **196** | **212** | **213** |
| 82 | **128** | **102** | **116** | **237** |
| 83 | **169** | **267** | **213** | **187** |
| 84 | **78** | **134** | **237** | **117** |
| 85 | **200** | **261** | **229** | **54** |
| 86 | **90** | **198** | **187** | **58** |
| 87 | **137** | **269** | **156** | **56** |
| 88 | **74** | **155** | **117** | **88** |
| 89 | **14** | **19** | **233** | **53** |
| 90 | **270** | **103** | **115** | **62** |
| 91 | **219** | **104** | **168** | **52** |
| 92 | **126** | **273** | **266** | **94** |
| 93 | **5** | **172** | **282** | **63** |
| 94 | **259** | **69** | **285** | **85** |
| 95 | **147** | **130** | **279** | **61** |
| 96 | **136** | **20** | **280** | **45** |
| 97 | **224** | **144** | **284** | **57** |
| 98 | **203** | **237** | **276** | **59** |
| 99 | **158** | **244** | **286** | **39** |
| 100 | **251** | **175** | **275** | **98** |
| 101 | **121** | **166** | **287** | **82** |
| 102 | **209** | **170** | **248** | **110** |
| 103 | **227** | **162** | **283** | **60** |
| 104 | **161** | **174** | **281** | **112** |
| 105 | **10** | **22** | **106** | **55** |
| 106 | **139** | **146** | **277** | **195** |
| 107 | **138** | **229** | **225** | **38** |
| 108 | **80** | **129** | **79** | **65** |
| 109 | **282** | **120** | **265** | **247** |
| 110 | **202** | **23** | **304** | **25** |
| 111 | **157** | **268** | **140** | **66** |
| 112 | **239** | **187** | **223** | **70** |
| 113 | **220** | **156** | **257** | **75** |
| 114 | **81** | **131** | **181** | **64** |
| 115 | **76** | **12** | **278** | **83** |
| 116 | **221** | **18** | **296** | **36** |
| 117 | **285** | **2** | **169** | **299** |
| 118 | **51** | **271** | **77** | **178** |
| 119 | **260** | **274** | **224** | **100** |
| 120 | **8** | **204** | **118** | **301** |
| 121 | **9** | **236** | **241** | **297** |
| 122 | **111** | **50** | **27** | **298** |
| 123 | **123** | **218** | **158** | **300** |
| 124 | **252** | **262** | **258** | **89** |
| 125 | **181** | **253** | **270** | **108** |
| 126 | **180** | **197** | **251** | **107** |
| 127 | **231** | **7** | **303** | **51** |
| 128 | **124** | **145** | **211** | **260** |
| 129 | **182** | **125** | **290** | **214** |
| 130 | **149** | **254** | **96** | **229** |
| 131 | **163** | **11** | **177** | **156** |
| 132 | **13** | **128** | **28** | **233** |
| 133 | **160** | **78** | **119** | **115** |
| 134 | **101** | **200** | **176** | **168** |
| 135 | **205** | **90** | **154** | **266** |
| 136 | **112** | **137** | **242** | **282** |
| 137 | **73** | **74** | **21** | **285** |
| 138 | **72** | **14** | **1** | **279** |
| 139 | **143** | **219** | **215** | **280** |
| 140 | **167** | **126** | **122** | **284** |
| 141 | **199** | **5** | **171** | **276** |
| 142 | **87** | **259** | **184** | **286** |
| 143 | **216** | **147** | **272** | **275** |
| 144 | **153** | **136** | **24** | **287** |
| 145 | **206** | **203** | **6** | **248** |
| 146 | **234** | **121** | **207** | **283** |
| 147 | **165** | **209** | **196** | **281** |
| 148 | **110** | **227** | **102** | **277** |
| 149 | **240** | **161** | **267** | **79** |
| 150 | **3** | **10** | **134** | **265** |
| 151 | **256** | **139** | **261** | **140** |
| 152 | **105** | **138** | **198** | **223** |
| 153 | **176** | **80** | **269** | **257** |
| 154 | **68** | **202** | **155** | **181** |
| 155 | **148** | **157** | **19** | **278** |
| 156 | **133** | **239** | **103** | **169** |
| 157 | **151** | **220** | **104** | **77** |
| 158 | **275** | **81** | **273** | **224** |
| 159 | **109** | **76** | **172** | **118** |
| 160 | **210** | **221** | **69** | **241** |
| 161 | **179** | **8** | **130** | **27** |
| 162 | **97** | **9** | **20** | **158** |
| 163 | **230** | **111** | **144** | **258** |
| 164 | **287** | **123** | **244** | **270** |
| 165 | **108** | **252** | **175** | **251** |
| 166 | **283** | **231** | **166** | **303** |
| 167 | **95** | **124** | **170** | **211** |
| 168 | **71** | **149** | **162** | **177** |
| 169 | **84** | **163** | **174** | **28** |
| 170 | **86** | **13** | **22** | **119** |
| 171 | **4** | **160** | **146** | **21** |
| 172 | **249** | **101** | **129** | **1** |
| 173 | **255** | **205** | **120** | **215** |
| 174 | **117** | **73** | **23** | **122** |
| 175 | **276** | **72** | **268** | **171** |
| 176 | **91** | **143** | **131** | **184** |
| 177 | **132** | **167** | **12** | **272** |
| 178 | **284** | **199** | **18** | **24** |
| 179 | **235** | **87** | **2** | **6** |
| 180 | **245** | **216** | **271** | **207** |
| 181 | **92** | **153** | **274** | **196** |
| 182 | **93** | **206** | **204** | **102** |
| 183 | **99** | **234** | **236** | **267** |
| 184 | **243** | **165** | **50** | **261** |
| 185 | **115** | **110** | **218** | **198** |
| 186 | **168** | **240** | **262** | **269** |
| 187 | **266** | **3** | **253** | **155** |
| 188 | **279** | **256** | **197** | **19** |
| 189 | **280** | **105** | **7** | **103** |
| 190 | **286** | **68** | **145** | **104** |
| 191 | **248** | **148** | **125** | **273** |
| 192 | **281** | **133** | **254** | **172** |
| 193 | **277** | **109** | **11** | **69** |
| 194 | **191** | **210** | **128** | **130** |
| 195 | **79** | **179** | **78** | **20** |
| 196 | **159** | **97** | **200** | **144** |
| 197 | **140** | **230** | **90** | **244** |
| 198 | **223** | **108** | **137** | **175** |
| 199 | **257** | **95** | **74** | **166** |
| 200 | **278** | **71** | **14** | **170** |
| 201 | **296** | **84** | **219** | **162** |
| 202 | **127** | **86** | **126** | **174** |
| 203 | **77** | **4** | **5** | **22** |
| 204 | **118** | **249** | **259** | **146** |
| 205 | **241** | **255** | **147** | **129** |
| 206 | **27** | **117** | **136** | **120** |
| 207 | **258** | **91** | **203** | **23** |
| 208 | **211** | **132** | **121** | **268** |
| 209 | **290** | **235** | **209** | **131** |
| 210 | **96** | **245** | **227** | **12** |
| 211 | **177** | **92** | **161** | **18** |
| 212 | **28** | **93** | **10** | **2** |
| 213 | **119** | **99** | **139** | **271** |
| 214 | **54** | **54** | **138** | **274** |
| 215 | **58** | **58** | **80** | **204** |
| 216 | **56** | **56** | **202** | **236** |
| 217 | **88** | **88** | **157** | **50** |
| 218 | **53** | **53** | **239** | **218** |
| 219 | **62** | **62** | **220** | **262** |
| 220 | **52** | **52** | **81** | **253** |
| 221 | **94** | **94** | **76** | **197** |
| 222 | **63** | **63** | **221** | **7** |
| 223 | **85** | **85** | **8** | **145** |
| 224 | **61** | **61** | **9** | **125** |
| 225 | **45** | **45** | **111** | **254** |
| 226 | **57** | **57** | **123** | **11** |
| 227 | **59** | **59** | **252** | **128** |
| 228 | **39** | **39** | **231** | **78** |
| 229 | **98** | **98** | **124** | **200** |
| 230 | **82** | **82** | **149** | **90** |
| 231 | **60** | **60** | **163** | **137** |
| 232 | **55** | **55** | **13** | **74** |
| 233 | **195** | **195** | **160** | **14** |
| 234 | **188** | **188** | **101** | **219** |
| 235 | **38** | **38** | **205** | **126** |
| 236 | **193** | **193** | **73** | **5** |
| 237 | **65** | **65** | **72** | **259** |
| 238 | **247** | **247** | **167** | **147** |
| 239 | **25** | **25** | **199** | **136** |
| 240 | **66** | **66** | **87** | **203** |
| 241 | **70** | **70** | **216** | **121** |
| 242 | **75** | **75** | **153** | **209** |
| 243 | **189** | **189** | **206** | **227** |
| 244 | **64** | **64** | **234** | **161** |
| 245 | **263** | **263** | **165** | **10** |
| 246 | **208** | **208** | **240** | **139** |
| 247 | **83** | **83** | **3** | **138** |
| 248 | **36** | **36** | **256** | **80** |
| 249 | **228** | **228** | **105** | **202** |
| 250 | **299** | **299** | **68** | **157** |
| 251 | **246** | **246** | **148** | **239** |
| 252 | **178** | **178** | **133** | **220** |
| 253 | **100** | **100** | **109** | **81** |
| 254 | **185** | **185** | **210** | **76** |
| 255 | **222** | **222** | **179** | **221** |
| 256 | **113** | **113** | **97** | **8** |
| 257 | **190** | **190** | **230** | **9** |
| 258 | **142** | **142** | **95** | **111** |
| 259 | **186** | **186** | **71** | **123** |
| 260 | **301** | **301** | **84** | **252** |
| 261 | **297** | **297** | **86** | **231** |
| 262 | **302** | **302** | **4** | **124** |
| 263 | **298** | **298** | **249** | **149** |
| 264 | **300** | **300** | **255** | **163** |
| 265 | **89** | **89** | **91** | **13** |
| 266 | **183** | **183** | **132** | **160** |
| 267 | **114** | **114** | **235** | **101** |
| 268 | **192** | **192** | **245** | **205** |
| 269 | **107** | **107** | **92** | **73** |
| 270 | **194** | **194** | **93** | **72** |
| 271 | **214** | **214** | **99** | **167** |
| 272 | **37** | **37** | **37** | **199** |
| 273 | **49** | **49** | **49** | **87** |
| 274 | **293** | **293** | **293** | **216** |
| 275 | **35** | **35** | **35** | **153** |
| 276 | **46** | **46** | **46** | **206** |
| 277 | **292** | **292** | **292** | **234** |
| 278 | **31** | **31** | **31** | **165** |
| 279 | **289** | **289** | **289** | **240** |
| 280 | **32** | **32** | **32** | **3** |
| 281 | **34** | **34** | **34** | **256** |
| 282 | **40** | **40** | **40** | **105** |
| 283 | **15** | **15** | **15** | **68** |
| 284 | **48** | **48** | **48** | **148** |
| 285 | **41** | **41** | **41** | **133** |
| 286 | **288** | **288** | **288** | **109** |
| 287 | **291** | **291** | **291** | **210** |
| 288 | **33** | **33** | **33** | **179** |
| 289 | **29** | **29** | **29** | **97** |
| 290 | **17** | **17** | **17** | **230** |
| 291 | **43** | **43** | **43** | **95** |
| 292 | **294** | **294** | **294** | **71** |
| 293 | **47** | **47** | **47** | **84** |
| 294 | **295** | **295** | **295** | **86** |
| 295 | **141** | **141** | **141** | **4** |
| 296 | **44** | **44** | **44** | **249** |
| 297 | **67** | **67** | **67** | **255** |
| 298 | **30** | **30** | **30** | **91** |
| 299 | **150** | **150** | **150** | **132** |
| 300 | **42** | **42** | **42** | **235** |
| 301 | **26** | **26** | **26** | **245** |
| 302 | **238** | **238** | **238** | **92** |
| 303 | **16** | **16** | **16** | **93** |
| 304 | **217** | **217** | **217** | **99** |
